# Superoxide chemistry revisited: synthesis of tetrachloro-substituted methylenenortricyclenes

**DOI:** 10.3762/bjoc.10.264

**Published:** 2014-10-30

**Authors:** Basavaraj M Budanur, Faiz Ahmed Khan

**Affiliations:** 1 Department of Chemistry, Indian Institute of Technology Kanpur, Kanpur-208016, India; 2 Department of Chemistry, Indian Institute of Technology Hyderabad, Ordnance Factory Estate, Yeddumailaram-502205, India

**Keywords:** BHT, dehydrohalogenation/rearrangement, Friedel–Crafts acylation, methylenenortricyclene, superoxide ion

## Abstract

An unexpected reactivity of the superoxide ion leading to the synthesis of tetrachloroaryl/vinyl-substituted nortricyclenes through its dual mode of action has been reported. KO_2_ was found to be superior and the only reagent to perform this kind of reaction over other conventional bases. Addition of the antioxidant BHT (2,6-di-*tert*-butyl-4-methylphenol) improved the yields of methylenenortricyclenes. A complete deuterium incorporation was observed in the superoxide-mediated reaction in DMSO-*d*_6_. Friedel–Crafts acylation reactions of 3-methylenenorticyclenes yielded 2-propanone-substituted pentachloronorbornenes.

## Introduction

The chemistry of the superoxide ion (O_2_**^−·^**) has been a subject of growing interest because of its presence in all aerobic organisms as a respiratory intermediate [1–6]. The study of the reactivity of the superoxide ion with organic substrates can facilitate understanding its role in metabolic processes [5]. Despite its biochemical importance, in organic synthesis, the superoxide ion has mainly been used for electron transfer reactions [7–9], oxidation [10–15], hydrolysis and substitution reactions [16–18]. Filippo and co-workers [19–22] have extensively studied the substitution reactions of alkyl halides and tosylates; oxidative cleavage reactions and hydrolysis of esters. Frimer and co-workers [23–24] have studied its reactivity with different functionalities. Jiang and co-workers [25] used KO_2_ as an alternative oxidation reagent in a Winterfeldt reaction instead of O_2_/KO*t-*Bu and many others reported reactions of superoxide which acts as oxidant, reductant, oxygen nucleophile, or Brønsted base. However, the full potential of the superoxide ion as a reagent in organic synthesis is still underexplored.

**Scheme 1:**
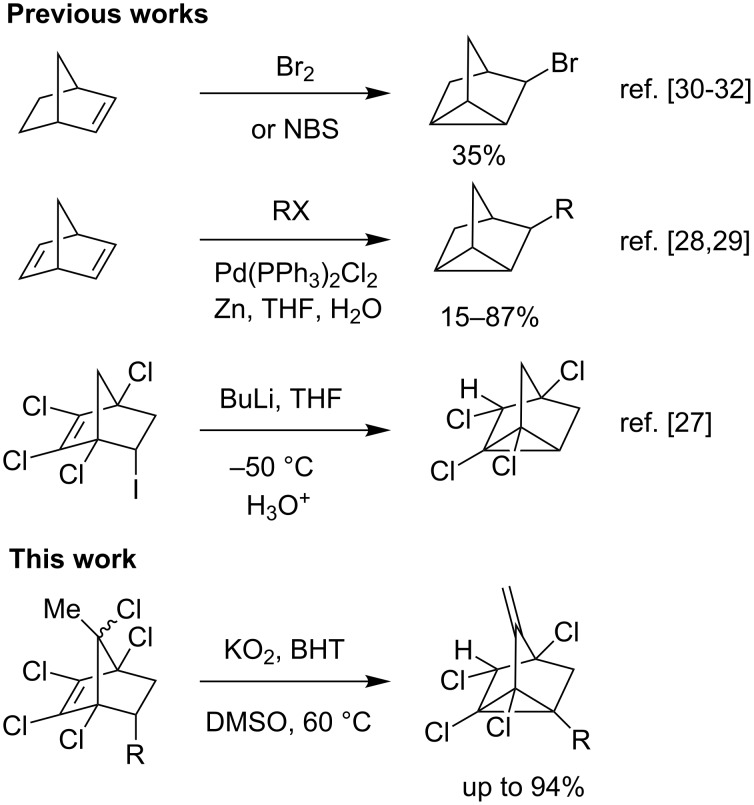
Synthesis of Nortricyclenes from Norbornenes.

A very few and scattered reports appeared in the literature for the conversion of bicyclic norbornene to substituted nortricyclenes (Scheme 1). In 1966 Kempter and co-workers [26] have synthesized nortricyclenes from norcamphor using *o*-amino acetophenone hydrochloride. Ladenberger and co-worker [27] observed the rearrangement of a lithiated-tetrachloronorbornyl intermediate to a tricyclene derivative in a study about stability of vinyl carbanions. Li and co-workers [28–29] have synthesized aryl/benzyl substituted nortricyclenes using palladium and zinc powder from norbornadiene. The bromination of norbornene [30–32] is another route to the nortricyclenes. Here, in this study we report an efficient method for the synthesis of aryl- and vinyl-substituted tetrachloromethylenenortricyclenes from the Diels–Alder (DA) adducts of pentachloro-5-methylcyclopentadiene **1** and styrenes, by concurrent dehydrohalogenation and rearrangement reactions, starting from the commercially available inexpensive reagent potassium superoxide.

## Results and Discussion

We have successfully utilized polyhalogenated bicyclic compounds for the synthesis of natural and aesthetically pleasing unnatural products. During our endeavour, we were selectively removing the halogens, utilizing them as transformation tools [33–36]. Our continued interest in this direction led us to explore the chemistry of alkylpentachlorocyclopentadienes as they have not been studied systematically even though discovered more than half a century ago. Alkylpentachlorocyclopentadienes can be easily prepared from hexachlorocyclopentadiene by treatment with the corresponding phosphite esters by a known protocol [37]. 5-Alkylpentachlorocyclopentadienes were rarely used as dienes in DA reactions; this may be because of their inverse-electron demand thus restricting them to DA reactions with electron-rich dienophiles. During our studies we noticed that pentachloro-5-methylcyclopentadiene (**1**) underwent a smooth DA reaction with styrenes (Table 1).

**Table 1:** Preparation of pentachloro-7-methyl substituted norbornenes^a,b^.

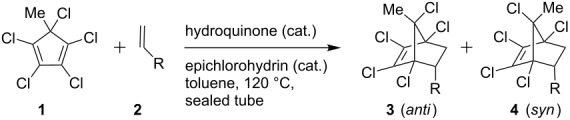				

Entry	R	Product(s)^a^	Time(h)	Yield(%)^c^

1	Ph	**3a**/**4a**	6	83
2	4-OMeC_6_H_4_	**3b**/**4b**	8	82
3	3,4,5-(OMe)_3_C_6_H_2_	**3c**/**4c**	14	82
4	2,5-(OMe)_2_C_6_H_3_	**3d**/**4d**	12	87
5^d^	4-NO_2_C_6_H_4_	**3e**	8	75
6	4-biphenyl	**3f**/**4f**	15	94
7	1-naphthyl	**3g**/**4g**	16	92
8	2-MeC_6_H_4_	**3h**/**4h**	15	86
9^e^	4-iPrC_6_H_4_	**3i**/**4i**	23	89
10^d^	4-Py	**3j**	15	77
11	2-BrC_6_H_4_	**3k**/**4k**	30	77
12^d^	2,4-(Cl)_2_C_6_H_3_	**3l**	20	70
13	-COMe	**3m**/**4m**	10	95
14^d^	CN	**3n**	17	92
15	-(CH_2_)_6_-	**3o**/**4o**	15	81
16	α-Me-vinyl	**3p**^f^	–	83

^a^Unless otherwise mentioned *anti*:*syn* ratio was ~4:1 and was calculated based on ^1^H NMR of the crude product; ^b^dienophiles were prepared according to the known procedures [38–40]; ^c^isolated yields; ^d^only anti (**3**) isomers were isolated in these cases; ^e^*anti*:*syn* ratio was 93:7; ^f^**3p** was prepared by Wittig reaction on **3m**.

We then prepared the series of DA adducts **3**/**4** from pentachloro-5-methylcyclopentadiene **1** and corresponding dienophiles **2** [38–40] by heating at 120–130 °C in a sealed tube. Styrene derivatives containing groups like methoxy, alkyl, naphthyl, and biphenyl (Table 1, entries 1–4, 6–9 ), methyl vinyl ketone (Table 1, entry 13) and cyclooctene (Table 1, entry 15) have furnished DA adducts in excellent yields and good diastereoselectivity ~4:1 (*anti*:*syn*) in 6–15 h. Some dienophiles such as *p*-nitrostyrene (Table 1, entry 5), 4-vinylpyridine (Table 1, entry 10), chlorinated styrene (Table 1, entry 12) and acrylonitrile (Table 1, entry 14) gave corresponding adducts in good yield and single *anti*-diastereomer only. The stereostructures of the DA adducts **3** and **4** were deduced from the literature established [41] ^1^H NMR pattern of the downfield shift (δ = ~1.8) for the *syn* methyl protons in the pentachloro DA adducts **4** and upfield shift (δ = ~1.6) for the *anti*-methyl protons in the pentachloro DA adducts **3**.

Having adduct **3a** in hand, we turned our attention to a dehydrochlorination to create a double bond at the C-7 position. Unfortunately the use of conventional bases like KO*t-*Bu, DBU, 2,6-lutidine, NaOH and NaOMe did not yield the desired product even at elevated temperatures. Then we looked for a hydrodechlorination at the C-7 position and found from the literature that zinc/AcOH [42–43] can be used for the reduction of chlorine at the C-7 position of a vinylacetate DA adduct of hexachlorocyclopentadiene, but only 6% of the bis-reduced product was obtained. We have slightly modified these conditions using an excess of zinc on substrate **3a**, but failed to get the reduced product.

**Figure 1:**
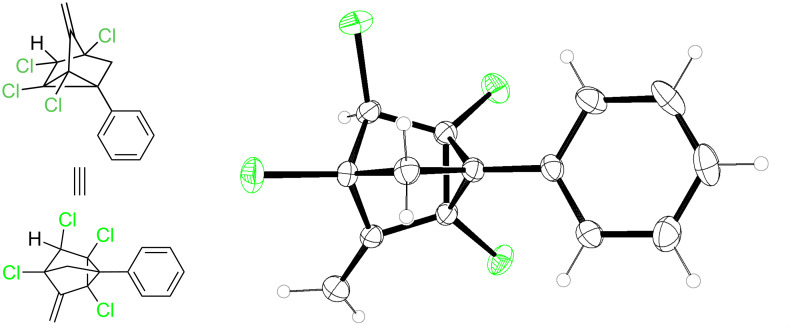
X-ray crystal structure of **5a** with 30% thermal ellipsoids.

A literature search also revealed that potassium superoxide has been used for selective monodehalogenation and elimination reactions in polyhalogenated compounds [44–45]. Treatment of adduct **3a** with potassium superoxide at 60 °C under argon atmosphere gave a colourless compound. Initially, we expected it to be the elimination product because of the two olefinic peaks in the ^1^H NMR spectrum but the presence of a singlet at δ 4.2 and only two olefinic peaks in the ^13^C NMR are not in accordance with our expectations. The single crystal X-ray analysis of the product (Figure 1) sorted out all the ambiguity associated with structure. To our pleasant surprise the structure of the product was methylenenortricyclene **5a** which seems to have been formed from a concurrent elimination as well as a rearrangement reaction.

In superoxide chemistry many aprotic solvents have been used. We prefer DMSO because the solubility of potassium superoxide is better in DMSO compared to other solvents [16]. As the phase-transfer catalyst can enhance the extent of solubility of the KO_2_ [16], we tried to use 18-crown-6 and to change the temperature to effect the rate of the reaction. We did not notice any significant difference in the rate under these conditions.

**Scheme 2:**
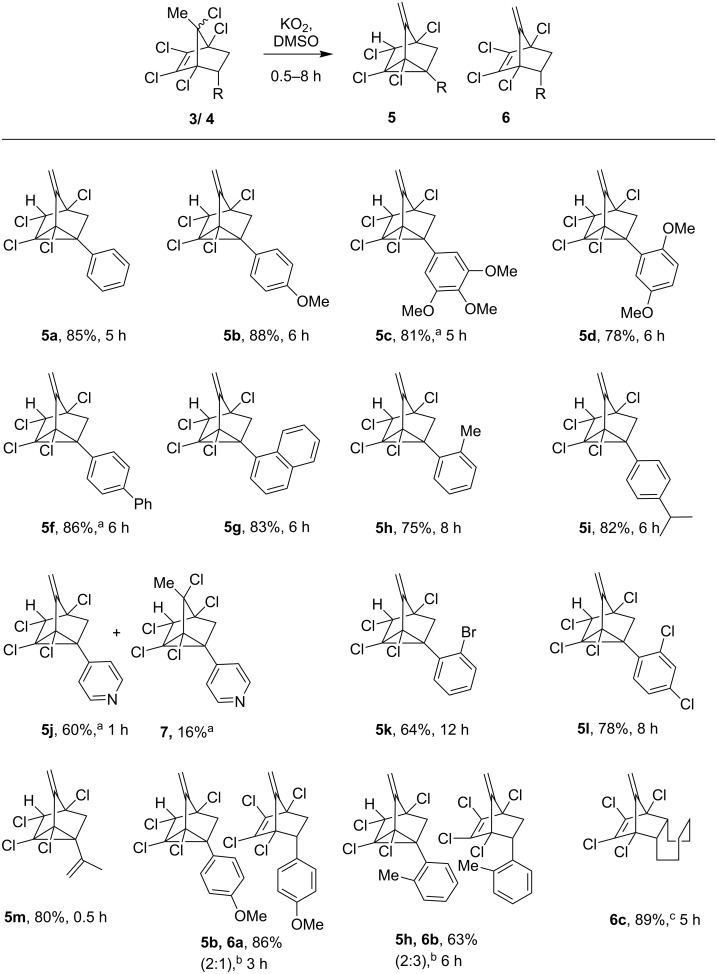
KO_2_-mediated synthesis of tetrachloro-substituted 3-methylenenortricyclenes. Reaction conditions: All the substrates are a mixture of *syn* and *anti* with KO_2_ (3 equiv)/DMSO at 60 °C, isolated yields are given. ^a^Reaction performed at rt, ^b^*syn*-isomers **4b** and **4h** gave mixtures of **5** and **6**. ^c^Reaction performed at 100 °C.

To study the scope and limitations and to probe the electronic and steric effects of this reaction, substituted styrene adducts were subjected to the reaction with KO_2_ (3 equiv) in DMSO and the results are summarized in Scheme 2. The substrates endowed with electron donating groups, for example *anti*-adduct **3b** gave methylenenortricyclene **5b**, whereas its *syn*-isomer **4b**, after consumption of all the starting material in 3 h furnished eliminated product **6a**, along with **5b**. Further treatment of the inseparable mixture of **5b**/**6a** with KO_2_ afforded exclusively nortricyclene **5b** in 80% overall yield from **4b**. The 3,4,5-trimethoxy-substituted DA adducts **3c**/**4c** gave title product **5c** at rt. The *o-*methoxy-substituted adducts **3d**/**4d** gave nortricyclene **5d** in good yield (78%). The substrate bearing an NO_2_ group on the phenyl ring **3e** (Table 1, entry 5) was completely unreactive even with excess of KO_2_ at up to 100 °C. The formation of a nitrobenzyl radical anion [9,22] by one-electron transfer might be the reason for this.

Biphenyl-substituted adducts **3f**/**4f** yielded nortricyclene derivative **5f** smoothly at rt. But naphthyl-substituted adducts **3g**/**4g** furnished the title product **5g** at 60 °C, probably due to steric effects (Scheme 2). The other substrates having alkyl substituents on the phenyl ring **3h**, **3i**/**4i** afforded the corresponding nortricyclenes in good yields, whereas *syn*-isomer **4h** yielded an inseparable mixture of **5h** and **6b**. We have examined the influence of heteroarene substitution towards this reaction using adduct **3j** which afforded nortricyclene **5i** in 60% and rearranged product **7** in 16% yield at rt after 30 min. Adducts **3k**/**4k** containing bromine at *ortho* position, a relatively bulky substituent close to the reactive site, took a little longer time to afford the nortricyclene **5k**. The 2,4-dichloro-substituted adduct **3l** gave a good yield of the desired product **5m** (78%) (Scheme 2).

In order to explore this unexpected reaction with respect to substrates containing other functional groups which are known to stabilize the anion were subjected with KO_2_ (Table 1); the acetyl- and cyano-substituted adducts **3m**/**4m** and **3n** (Table 1, entries 13, 14) did not yield any product and resulted in substantial loss of starting material. The olefin-substituted compound **3p** (Table 1, entry 16) underwent a smooth reaction to afford nortricyclene **5m** (Scheme 2). The cyclooctane-fused adducts **3o**/**4o** (Table 1, entry 15) afforded exclusively the elimination product **6c** in excellent yield.

As the p*K*_a_ of the conjugate acid of the superoxide ion, the hydroperoxyl radical is 4.88 [46] in water which is similar to that of acetic acid; it implies that the superoxide ion is a weak base. Although basicity of the anion is higher in nonprotic solvents than in protic solvents [18], KOAc can be a surrogate to KO_2_. Treating adduct **3a** with KOAc in DMSO did not yield any product which suggested that there is a unique and exceptional degree of reactivity of the superoxide ion species which triggered this kind of reaction.

**Scheme 3:**
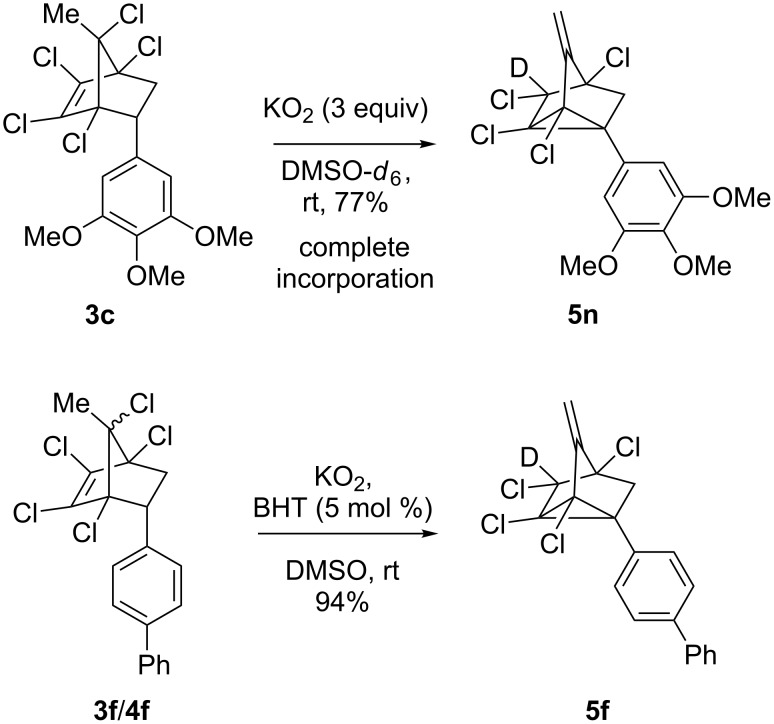
Mechanism investigations.

To elucidate the reaction mechanism, we have performed a deuterium labelling experiment by treating **3c** with KO_2_ in DMSO-*d*_6_ at rt. Absence of the peak at δ 4.26 (singlet) in ^1^H NMR suggest the incorporation of deuterium and a triplet at δ 71.0, arising from C–D coupling, instead of sharp peak at δ 71.3 in ^13^C NMR further confirmed the deuteration at the chlorine-attached carbon atom in **5n** (Scheme 3). Further, in order to understand the mechanistic aspects of the reaction we have treated **3f**/**4f** under standard conditions by using the additive radical quencher and antioxidant BHT (5 mol %) afforded the product **5f** in improved yield of 94% (Scheme 3). Similarly, substrates **3c**/**4c** and **3i**/**4i** afforded **5c** and **5i** in 91% and 89% yield, respectively. This observation provides added evidence that the mechanism involved might be ionic.

**Scheme 4:**
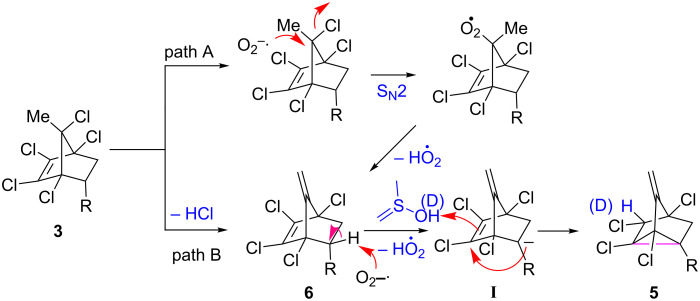
Plausible mechanism of the KO_2_-mediated reaction.

Based on known chemistry in the literature, initially we have assumed two different possible pathways for the formation of product **6**: The nucleophilic substitution of the superoxide ion followed by elimination (path A) or the direct dehydrohalogenation (path B) [47] (Scheme 4). We ruled out path A based on our observation that even after adding BHT, the reaction underwent smoothly. The second step involved the deprotonation of a benzylic proton by the superoxide ion [48]. The resulting benzylic anion **I** undergoes an unexpected rearrangement by adding onto the double bond possessing the vicinal chlorines [8] and the resulting carbanion is protonated by the solvent DMSO to afford the nortricyclene **5**.

**Figure 2:**
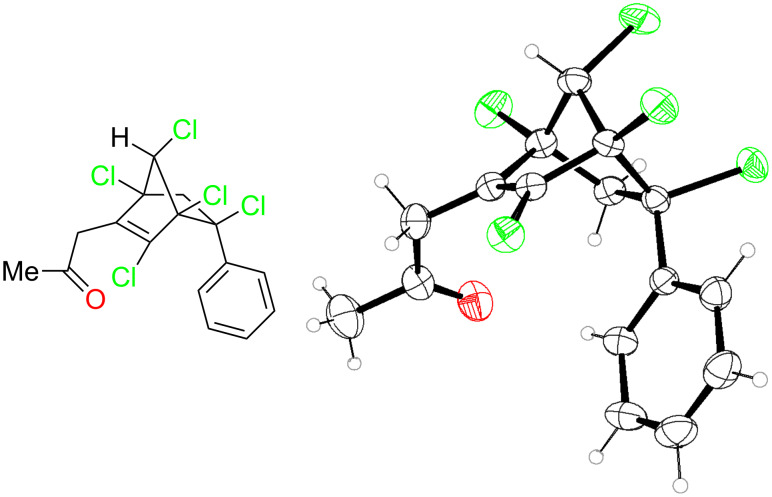
X-ray crystal structure of **8a** with 30% thermal ellipsoids.

In the next part of our study, we have investigated the acylation reactions as it was reported that acylation reactions of parent nortricyclenes [49] proceed through initial formation of chloroacetylated norbornane which was rapidly dehydrohalogenated upon storage at rt to give 1-acetylnortricyclene. We have applied the standard acylation reaction conditions (AlCl_3_, R^1^COCl, 1:1) to 3-methylenenortricyclenes to observe acylation on the double bond and not on the cyclopropane ring to give 2-propanone-substituted pentachloronorbornene derivatives **8** (Table 2). The structure of **8a** was unambiguously proved by single crystal X-ray analysis (Figure 2).

**Table 2:** Acylation reaction of 3-methylenenortricyclenes.

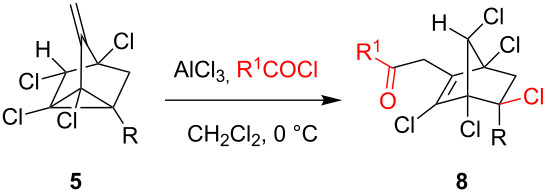		

R	R^1^	product/yield(%)^a^

Ph, **5a**	Me	**8a**/76
Ph, **5a**	Ph	**8b**/61
BiPh, **5f**	Me	**8c**/68^b^

^a^Isolated yields; ^b^acetylation also occurred at the 4'-positon of the biphenyl ring.

**Scheme 5:**
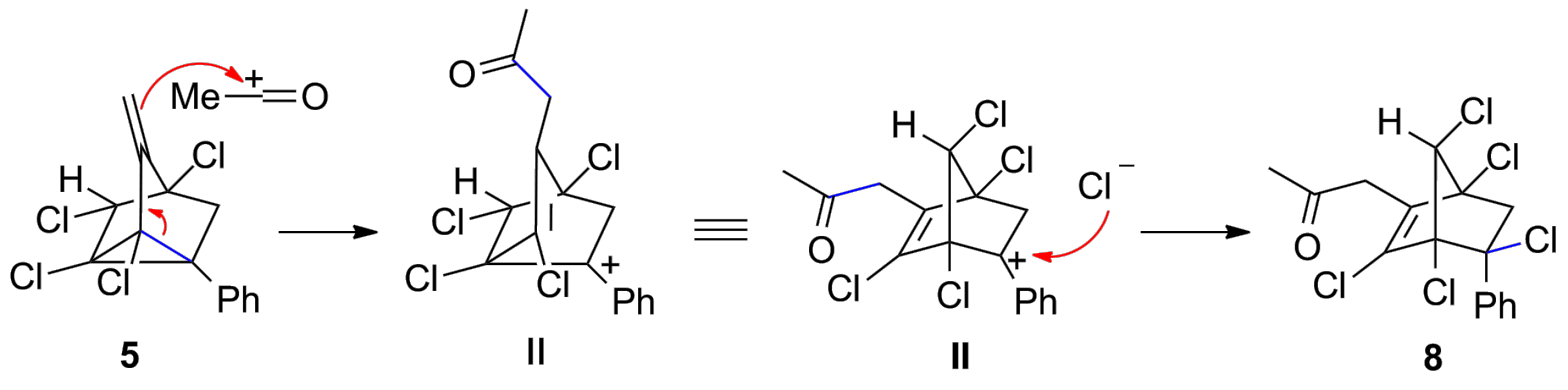
Plausible mechanism of the acylation reaction of 3-methylenenortricyclenes.

A plausible mechanism involving the initial nucleophilic attack of the exocyclic olefin on the acylium ion with concomitant cyclopropane ring opening leading to benzylic cation **II** followed by its combination with chloride ion leading to **8** is proposed (Scheme 5).

## Conclusion

In summary, we have demonstrated the preparation of a variety of Diels–Alder adducts pentachloro-7-methyl-5-aryl/vinyl-substituted norbornenes and utilized them in the synthesis of tetrachloro aryl/vinyl-substituted methylenenortricyclenes efficiently with good to excellent yields using inexpensive and commercially available potassium superoxide. The reaction mechanism of the KO_2_-mediated reaction was derived through a controlled experiment with BHT which improved the yields of nortricyclenes and a deuterium labelling experiment using DMSO-*d*_6_. Further, acylation reactions of the synthesized methylenenortricyclens afforded the 2-propanone-substituted pentachloronorbornenes in good yields. The studies towards further synthetic applications of KO_2_ are currently underway in our lab.

## Supporting Information

Experimental procedures and analytical data, including copies of ^1^H and ^13^C NMR spectra for all new compounds. Crystallographic data for structures **5a** and **8a** have been deposited with the Cambridge Crystallographic Data Centre as supplementary publication nos. CCDC 1021351 and 1021303. Copies of the data can be obtained, free of charge, on application to CCDC, 12 Union Road, Cambridge, CB2 1EZ, UK (fax: +44-(0)1223-336033 or e-mail: deposit@ccdc.cam.ac.ukor via: http://www.ccdc.cam.ac.uk).

File 1 Experimental part and NMR spectra.
