# Supplementary material for: Superoxide chemistry revisited: synthesis of tetrachloro-substituted methylenenortricyclenes
Source: Beilstein J Org Chem. 2014 Oct 30;10:2531–8. doi: 10.3762/bjoc.10.264 (PMC4222393; doi:10.3762/bjoc.10.264)
Supplement: File 1 — Experimental part and NMR spectra. [file Beilstein_J_Org_Chem-10-2531-s001.pdf]

Supporting Information

for

Superoxide chemistry revisited: synthesis of tetrachloro-substituted methylenenortricyclenes

Basavaraj M. Budanur[1] and Faiz Ahmed Khan*[2]

Address: [1]Department of Chemistry, Indian Institute of Technology Kanpur, Kanpur-208016, India and [2]Department of Chemistry, Indian Institute of Technology Hyderabad, Ordnance Factory Estate, Yeddumailaram-502205, India

Email: Faiz Ahmed Khan* - faiz@iith.ac.in

*Corresponding author

Experimental part and NMR spectra

General methods **S2**

General procedure for the preparation of Diels–Alder adducts 3/4 **S2**

^{1}H NMR, ^{13}C NMR NMR data of DA adducts 3/4 **S2**

General procedure for the KO_2 Mediated reaction **S12**

^{1}H NMR, ^{13}C NMR NMR data of the nortricyclenes **S13**

General procedure for the acylation reaction of nortricyclenes **S21**

^{1}H NMR, ^{13}C NMR NMR data of products of acylation reaction **S21**

Copies of ^{1}H NMR, ^{13C} NMR spectra of DA adducts 3/4 **S24**

Copies of ^{1}H NMR, ^{13}C NMR spectra of nortricyclenes 5 **S51**

Copies of ^{1}H NMR, ^{13C} NMR spectra of products of acylation reaction **S68**

Crystal data and structure refinement of compound 5a **S71**

Crystal data and structure refinement of compound **S72**

Reference **S72**

General methods: Melting points are uncorrected. Unless otherwise specified, all reactions were carried out in oven-dried glassware, under argon atmosphere. ^{1}H and proton decoupled ^{13}C NMR spectra were recorded in 400 and 100 MHz, respectively, unless otherwise mentioned at field strength of 500 MHz (^{1}H NMR) and 125 MHz (^{13}C NMR). The NMR samples were prepared by dissolving in $CDCl_3$, chemical shifts are reported in parts per million downfield from TMS as an internal standard and the multiplicity are reported as follows s = singlet, d = doublet, dd = double doublet, t = triplet, dt = double triplet, td = triple doublet, q = quartet, dq = double quartet, m = multiplet and the coupling constants were in Hz. The powdered potassium superoxide was purchased from Alfa Aesar and used as such. 'Caution should be taken when using KO_2, since it is explosive'.

General procedure for the preparation of Diels–Alder 3/4: The mixture of 1,2,3,4,5-pentachloro-5-methylcyclopenta-1,3-diene **1** (1 g, 3.96 mmol), styrene **2a** (825 mg, 7.92 mmol), catalytic amount of hydroquinone (3 mg), epichlorohydrin (0.01 mL) in dry toluene (1.5 mL) was heated at 120 °C - 130 °C in a sealed tube. After being heated for 10 h (reaction monitored by TLC), the reaction mixture was concentrated and the resulting crude residue was purified over silica gel column chromatography using hexane as eluent to afford adducts **3a** and **4a** (1.15 g, 83%, in 4:1 ratio) as colourless solids. Spectral data matching with reported one[1]

^{1}H NMR, ^{13}C NMR data of Diels–Alder adducts 3/4:

(1*R**,4*R****,5*R****,7*S****)-1,2,3,4,7-Pentachloro-7-methyl-5-phenylbicyclo[2.2.1]hept-2-ene** (**3a**); and **(1*R****,4*R****,5*R****,7*R****)-1,2,3,4,7-pentachloro-7-methyl-5-phenylbicyclo[2.2.1]hept-2-ene (4a)**

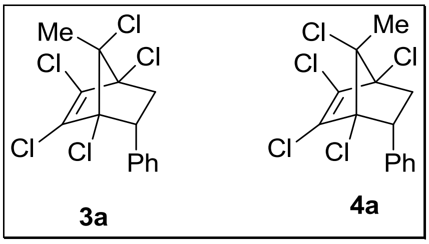

Data is matching with reported compounds; Data for **3a**: mp: 52-54 °C, R_f = 0.8 (hexane); ^{1}H NMR (400 MHz, $CDCl_3$) δ 7.40 - 7.30 (m, 3H), 7.16-7.05 (m, 2H), 4.09 (dd, *J* = 4.3, 9.3 Hz, 1H), 2.99 (dd, *J* = 9.3, 12.5 Hz, 1H), 2.43 (dd, *J* = 4.1, 12.6 Hz, 1H), 1.65 (s, 3H); ^{13}C NMR (100 MHz, $CDCl_3$) δ 135.2, 130.7, 130.6, 129.0, 128.3, 128.1, 88.6, 82.5, 77.4, 52.3, 41.9, 19.8; IR (neat): 3032, 1601, 1496, 1455, 1381, 1268, 1211, 1187, 1151, 1093, 1011, 951 cm^{-1}.

Data for **4a:** mp: 92-94 °C; R_f = 0.75 (hexane); ^{1}H NMR (400 MHz, $CDCl_3$) δ 7.39 - 7.29 (m, 3H), 7.15 - 7.07 (m, 2H), 3.67 (dd, *J* = 4.5, 9.3 Hz, 1H), 2.65 (dd, *J* = 9.3, 13.1 Hz, 1 H), 2.53 (dd, *J* = 4.6, 13.1 Hz, 1H), 1.83 (s, 3H); ^{13}C NMR (100 MHz, $CDCl_3$) δ 135.2, 132.0, 128.9, 128.4, 128.3, 91.9, 83.1, 77.4, 51.5, 40.4, 20.9; IR (neat): 3032, 1602, 1492, 1453, 1382, 1267, 1211, 1182, 1151, 1097, 1011, 951 cm^{-1}.

(1R^*,4R^*,5R^*,7S^*)-1,2,3,4,7-Pentachloro-5-(4-methoxyphenyl)-7-methylbicyclo[2.2.1]hept-2-ene (3b) and (1R^*,4R^*,5R^*,7R^*)-1,2,3,4,7-pentachloro-5-(4-methoxyphenyl)-7-methylbicyclo[2.2.1]hept-2-ene (4b):

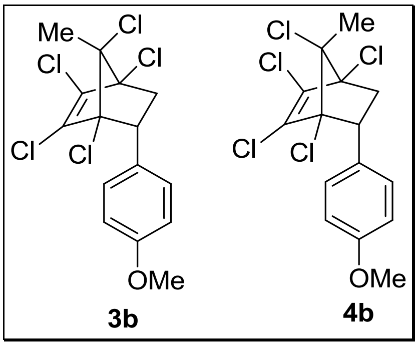

Following the same procedure as for **3a** starting from compound **1** (200 mg, 0.79 mmol) and 1-methoxy-4-vinylbenzene **2b** (211 mg, 1.58 mmol), compounds **3b** and **4b** (249 mg, 82%) were obtained in 3:1 ratio. Data for **3b**: colorless solid; mp: 90-92 °C; ^{1}H NMR (400 MHz, $CDCl_3$) δ 7.02 (d, *J* = 8.5 Hz, 2H), 6.85 (d, *J* = 8.7 Hz, 2H), 4.02 (dd, *J* = 9.2, 4.1 Hz, 1 H), 3.80 (s, 3H), 2.96 (dd, *J* = 12.6, 9.2 Hz, 1H), 2.35 (dd, *J* = 12.6, 4.1 Hz, 1H), 1.62 (s, 3H); ^{13}C NMR (125 MHz, $CDCl_3$) δ 159.5, 130.7, 130.6, 130.1 (2C), 127.2, 113.8 (2C), 88.6, 82.7, 76.8, 55.3, 52.3, 42.2, 20.0; IR (KBr): 3002, 2957, 2935, 2835, 1603, 1511, 1445, 1375, 1290, 1247, 1211, 1180, 1148, 1108, 1034, 1008, 947 cm^{-1}; HRMS (ESI): *m/z* calcd for $C_{15}H_{17}Cl_5NO$ $[M + NH_4]^+$ 403.9723; found: 403.9705.

Data for **4b**: colorless liquid; ^{1}H NMR (400 MHz, $CDCl_3$) δ 7.01 (d, *J* = 8.7 Hz, 2H), 6.85 (d, *J* = 8.8 Hz, 2H), 3.80 (s, 3H), 3.60 (dd, *J* = 9.1, 4.5 Hz, 1H), 2.64 (dd, *J* = 13.1, 9.2 Hz, 1H), 2.45 (dd, *J* = 13.1, 4.4 Hz, 1H), 1.79 (s, 3H); ^{13}C NMR (125 MHz, $CDCl_3$) δ 159.6, 132.1, 131.9, 130.0 (2C), 126.9, 113.9 (2C), 91.9, 83.2, 77.6, 55.3, 50.9, 40.7, 20.9; IR (neat): 3010, 2923, 2832, 1600, 1509, 1439, 1380, 1287, 1248, 1213, 1180, 1148, 1105, 1032, 998 cm^{-1}. HRMS (MMI): *m/z* calcd for $C_{15}H_{17}Cl_5NO$ $[M + NH_4]^+$ 403.9723; found: 403.9748.

(1R^*,4R^*,5R^*,7S^*)-1,2,3,4,7-Pentachloro-7-methyl-5-(3,4,5-trimethoxyphenyl)bicyclo[2.2.1]hept-2-ene (3c) and (1R^*,4R^*,5R^*,7R^*)-1,2,3,4,7-pentachloro-7-methyl-5-(3,4,5-trimethoxyphenyl)bicyclo[2.2.1]hept-2-ene (4c):

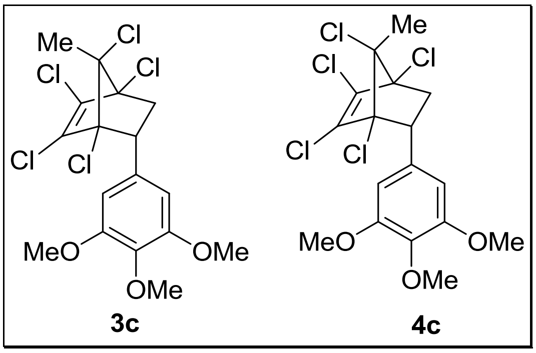

Following the same procedure as for **3a** starting from compound **1** (300 mg, 1.19 mmol) and 1,2,3-trimethoxy-5-vinylbenzene **2c** (256 mg, 1.3 mmol), compounds **3c** and **4c** (433 mg,

82%) were obtained in 4:1 ratio. Data for **3c**: colorless solid; mp: 110 - 112 °C; R_f = 0.5 (5% EtOAc in hexane); ^{1}H NMR (400 MHz, $CDCl_3$) δ 6.32 (s, 2H), 4.00 (dd, *J* = 9.3, 3.9 Hz, 1H), 3.85 (s, 6H), 3.84 (s, 3H), 3.00 (dd, *J* = 12.7, 9.3 Hz, 1H), 2.35 (dd, *J* = 12.7, 4.4 Hz, 1H), 1.64 (s, 3H); ^{13}C NMR (100 MHz, $CDCl_3$) δ 152.9 (2C), 137.8, 131.1, 130.7, 130.4, 106.1 (2C), 88.6, 82.6, 77.2, 60.9, 56.1 (2C), 53.3, 42.3, 19.7; IR (neat): 2989, 2935, 2833, 1603, 1587, 1510, 1460, 1453, 1420, 1382, 1346, 1328, 1268, 1238, 1152, 1127, 1092, 1005, 955 cm^{-1}; HRMS (APCI): *m/z* calcd for $C_{17}H_{18}Cl_5O_3[M + H]^+$ 446.9669; found: 446.9649.

Data for **4c**: colorless solid; mp: 80-82 °C, R_f = 0.45 (5% EtOAc in hexane); ^{1}H NMR (400 MHz, $CDCl_3$) δ 6.31 (s, 2H), 3.86 (s, 6H), 3.85 (s, 3H), 3.59 (dd, *J* = 9.3, 4.4 Hz, 1H), 2.68 (dd, *J* = 13.4, 9.3 Hz, 1H), 2.46 (dd, *J* = 13.7, 4.4 Hz, 1H), 1.80 (s, 3H); ^{13}C NMR (100 MHz, $CDCl_3$) δ 152.9 (2C), 138.0, 132.5, 131.6, 130.5, 106.1 (2C), 91.6, 83.1, 77.4, 60.9, 56.1 (2C), 52.0, 40.8, 20.8; IR (neat): 2990, 2932, 2833, 1600, 1582, 1513, 1460, 1451, 1422, 1382, 1342, 1325, 1273, 1242, 1152, 1128, 1092, 1013, 958 cm^{-1}; HRMS (ESI): *m/z* calcd for $C_{17}H_{21}Cl_5NO_3[M + NH_4]^+$ 463.9935; found: 463.9906.

(1*R,4*R**,5*R**,7*S**)-1,2,3,4,7-Pentachloro-5-(2,5-dimethoxyphenyl)-7-methylbicyclo[2.2.1] hept-2-ene (3d)** and **(1*R**,4*R**,5*R**,7R*)-1,2,3,4,7-pentachloro-5-(2,5-dimethoxyphenyl)-7-methylbicyclo[2.2.1]hept-2-ene (4d):**

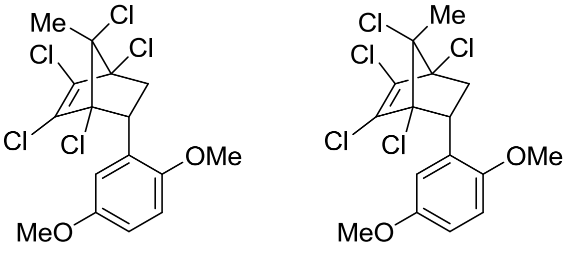

3d **4d**

Following the same procedure as for **3a** starting from compound **1** (500 mg, 1.98 mmol) and 1,4-dimethoxy-2-vinylbenzene **2d** (325 mg, 1.98 mmol), compounds **3d** and **4d** (712 mg, 87%) were obtained in 3:1 ratio. Data for **3d**: colorless liquid; R_f = 0.6 (5% EtOAc in hexane); ^{1}H NMR (400 MHz, $CDCl_3$) δ 6.84 - 6.77 (m, 2H), 6.43 (d, *J* = 2.9 Hz, 1H), 4.77 (dd, *J* = 4.4, 9.8 Hz, 1H), 3.82 (s, 3H), 3.77 (s, 3H), 2.95 (dd, *J* = 9.5, 12.5 Hz, 1H), 2.36 (dd, *J* = 4.4, 12.2 Hz, 1H), 1.64 (s, 3H); ^{13}C NMR (100 MHz, $CDCl_3$) δ 153.3, 152.7, 131.1, 130.7, 124.9, 115.0, 114.0, 112.0, 88.7, 82.2, 77.1, 56.3, 55.8, 45.2, 42.5, 19.7; IR (neat): 2991, 2932, 2830, 1602, 1539, 1500, 1462, 1452, 1449, 1420, 1386, 1350, 1296, 1260, 1181, 1118, 1097, 1046, 976 cm^{-1}; HRMS (APCI): *m/z* calcd for $C_{16}H_{15}Cl_5O_2[M]^+$ 415.9485; found: 415.9466.

Data for **4d**: colorless solid; mp: 100-102 °C; R_f = 0.55(5% EtOAc in hexane); ^{1}H NMR (400 MHz, $CDCl_3$) δ 6.87-6.76 (m, 2H), 6.40 (d, *J* = 2.4 Hz, 1H), 4.41 (dd, *J* = 4.6, 9.5 Hz, 1H), 3.82 (s, 3H), 3.77 (s, 3H), 2.65 (dd, *J* = 9.5, 13.0 Hz, 1H), 2.40 (dd, *J* = 4.6, 13.0 Hz, 1H), 1.84 (s, 3H); ^{13}C NMR (100 MHz, $CDCl_3$) δ 153.5, 152.5, 132.5, 132.0, 124.7, 114.6, 114.4,

112.0, 92.4, 82.7, 77.6, 56.3, 55.8, 43.1, 41.4, 21.0; IR (neat): 2994, 2935, 2833, 1600, 1547, 1501, 1463, 1453, 1443, 1421, 1387, 1351, 1297, 1264, 1261, 1180, 1154, 1116, 1096, 1046, 1034, 968 cm^{-1}; HRMS(APCI): *m/z* calcd for $C_{16}H_{15}Cl_5O_2$ $[M]^+$ 415.9485; found: 415.9466.

(1*R,4*R**,5*R**,7*S**)-1,2,3,4,7-Pentachloro-7-methyl-5-(4-nitrophenyl)bicyclo[2.2.1]hept-2-ene (3e):**

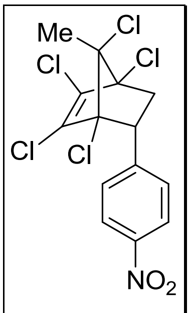

3e

Following the same procedure as for **3a** starting from compound **1** (100 mg, 0.39 mmol) and 1-nitro-4-vinylbenzene, **2e** (69 mg, 0.46 mmol), compound **3e** (118 mg, 75%) was obtained. colorless solid; mp: 130-132 °C; ^{1}H NMR (400 MHz, $CDCl_3$) δ 8.2 (d, *J* = 8.8 Hz, 2H), 7.30 (d, *J* = 8.5 Hz, 2H), 4.19 (dd, *J* = 9.2, 4.1 Hz, 1H), 3.04 (dd, *J* = 12.7, 9.2 Hz, 1H), 2.42 (dd, *J* = 12.9, 4.1 Hz, 1H), 1.64 (s, 3H); ^{13}C NMR (125 MHz, $CDCl_3$) δ 147.9, 142.9, 131.5, 130.1, 130.0 (2C), 123.5 (2C), 88.5, 82.4, 77.4, 52.8, 41.8, 19.7; IR (KBr): 3077, 1596, 1516, 1447, 1382, 1345, 1317, 1287, 1262, 1211, 1182, 1148, 1107, 1084, 1034, 1009 cm^{-1}; EI-HRMS: (*m/z*) M^+ calculated for $C_{14}H_{16}Cl_4$: 398.9154; found : 398.9156.

(1*R,4*R**,5*R**,7*S**)-5-(Biphenyl-4-yl)-1,2,3,4,7-pentachloro-7-methylbicyclo[2.2.1]hept-2-ene (3f) and (1*R**,4*R**,5*R**,7*R**)-5-(biphenyl-4-yl)-1,2,3,4,7-pentachloro-7-methylbicyclo[2.2.1]hept-2-ene (4f):**

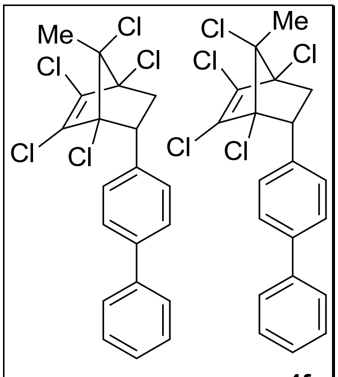

3f **4f**

Following the same procedure as for **3a** starting from compound **1** (200 mg, 0.79 mmol) and 4-vinylbiphenyl **2f** (157 mg, 0.87 mmol), compounds **3f** and **4f** (323 mg, 94%) were obtained in 74:26 ratio. Data for **3f**: colorless solid; mp: 96-98 °C, R_f = 0.55 (hexane); ^{1}H NMR (400 MHz, $CDCl_3$) δ 7.60-7.53 (m, 4H), 7.43 (t, *J* = 7.5 Hz, 2H), 7.36-7.32 (m, 1H), 7.17 (d, *J* =

8.3 Hz, 2H), 4.11 (dd, J = 9.2, 4.1 Hz, 1H), 3.00 (dd, J = 12.7, 9.1 Hz, 1H), 2.43 (dd, J = 12.8, 4.1 Hz, 1H), 1.65 (s, 3H); ^{13}C NMR (125 MHz, $CDCl_3$) δ 140.8, 140.3, 134.2, 130.7, 130.6, 129.4 (2C), 128.8 (2C), 127.4, 127.0 (2C), 126.9 (2C), 88.6, 82.5, 77.3, 52.6, 42.0, 19.7; IR (neat): 3040, 2923, 1600, 1518, 1485, 1442, 1380, 1261, 1190, 1157, 1092, 1028, 1005, 972, 963 cm^{-1}; HRMS (APCI): *m/z* calcd for $C_{20}H_{16}Cl_5K[M + H]^+$ 432.9665; found: 432.9644.

Data for **4f**: colourless solid; mp: 110-112 °C, R_f = 0.45 (hexane); ^{1}H NMR (400 MHz, $CDCl_3$) δ 7.57 (t, J = 8.3 Hz, 4H), 7.43 (t, J = 7.5 Hz, 2H), 7.37-7.33 (m, 1H), 7.16 (d, J = 8.3 Hz, 2H), 3.70 (dd, J = 9.0, 4.3 Hz, 1H), 2.69 (dd, J = 13.3, 9.1 Hz, 1H), 2.54 (dd, J = 13.1, 4.6 Hz, 1H), 1.82 (s, 3H); ^{13}C NMR (125 MHz, $CDCl_3$) δ 141.1, 140.2, 134.0, 132.0, 131.9, 129.3(2C), 128.8 (2C), 127.5, 127.03 (2C), 127.02 (2C), 91.8, 83.1, 77.5, 51.2, 40.5, 20.8; IR (neat): 3030, 2926, 1601, 1520, 1487, 1440, 1383, 1263, 1186, 1154, 1097, 1027, 1007, 979, 969 cm^{-1}; HRMS (ESI): *m/z* calcd for $C_{20}H_{15}Cl_5K$ $[M + K]^+$ 470.9224; found: 470.9230.

1-((1*R,4*R**,5*R**,7*S**)-1,4,5,6,7-Pentachloro-7-methylbicyclo[2.2.1]hept-5-en-2-yl)naphthalene (3g) and 1-((1*R**,4*R**,5*R**,7*R**)-1,4,5,6,7-pentachloro-7-methylbicyclo[2.2.1]hept-5-en-2-yl)naphthalene (4g):**

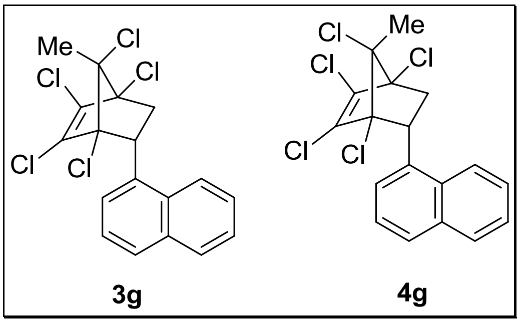

Following the same procedure as for **3a** starting from compound **1** (200 mg, 0.79 mmol) and 1-vinylnaphthalene, **2g** (146 mg, 0.95 mmol), compounds **3g** and **4g** (297 mg, 92%) were obtained in 4:1 ratio. Data for **3g**: colorless solid; mp: 128 - 130 °C, ^{1}H NMR (500 MHz, $CDCl_3$) δ 8.35 (d, J = 8.5 Hz, 1H), 7.86 (d, J = 8.0 Hz, 1H), 7.81 (d, J = 8.2, Hz, 1H), 7.59-7.55 (m, 1H), 7.51-7.419 (m, 1H) 7.43 (t, J = 7.8 Hz, 1H), 7.01 (d, J = 7.3 Hz, 1H), 5.26 (dd, J = 9.2, 4.2 Hz, 1H), 3.13 (dd, J = 12.7, 9.2 Hz, 1H), 2.51 (dd, J = 12.8, 4.2 Hz, 1H), 1.68 (s, 3H); ^{13}C NMR (125 MHz, $CDCl_3$) δ 134.0, 133.3, 132.1, 131.1. 131.0, 128.9, 128.7, 126.2, 125.9, 125.8, 125.2, 124.2, 89.4, 83.3, 77.4 (merged with $CDCl_3$ peak), 45.9, 44.2, 19.9; IR (KBr): 3078, 1599, 1509, 1438, 1378, 1264, 1206, 1147, 1100, 1005, 945 cm^{-1}; EI-HRMS: (m/z) M^+ calculated for $C_{14}H_{16}Cl_4$: 403.9460; found: 403.9462.

Data for **4g**: colorless solid; mp: 118-120 °C, ^{1}H NMR (500 MHz, $CDCl_3$) δ 8.30 (d, J = 8.5 Hz, 1H), 7.88 (d, J = 8.0 Hz, 1H), 7.82 (d, J = 8.2 Hz, 1H), 7.59-7.56 (m, 1H), 7.53-7.51 (m, 1H), 7.43 (t, J = 7.8 Hz, 1H), 7.02 (d, J = 7.3 Hz, 1H), 4.79 (dd, J =9.3, 4.4 Hz, 1H), 2.81 (dd, J = 13.1, 9.2 Hz, 1H), 2.51 (dd, J = 12.7, 4.1 Hz, 1H), 1.98 (s, 3H); ^{13}C NMR (125 MHz, $CDCl_3$) δ 133.8, 132.9, 132.3, 132.1, 131.7, 129.0, 128.9, 126.2, 126.0, 125.8, 125.2, 123.7,

92.3, 83.8, 77.5, 44.4, 42.7, 21.0; IR (KBr): 3077, 1598, 1509, 1438, 1393, 1261, 1148, 1095; EI-HRMS: (m/z) M^+ calculated for $C_{14}H_{16}Cl_4$: 403.9460; found: 403.9467.

(1*R,4*R**,5*R**,7*S**)-1,2,3,4,7-Pentachloro-7-methyl-5-o-tolylbicyclo[2.2.1]hept-2-ene (3h) and (1*R**,4*R**,5*R**,7*R**)-1,2,3,4,7-pentachloro-7-methyl-5-*o*-tolylbicyclo[2.2.1]hept-2-ene (4h):**

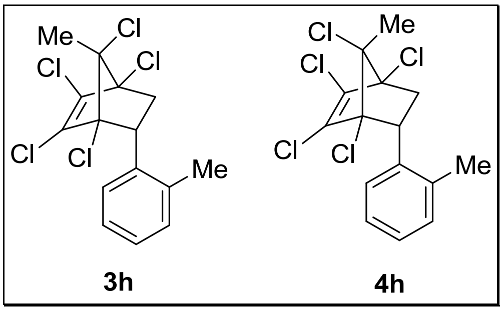

Following the same procedure as for **3a** starting from compound **1** (200 mg, 0.79 mmol) and 1-methyl-2-vinylbenzene, **2h** (112 mg, 0.95 mmol), compounds **3h** and **4h** (251 mg, 86%) were obtained in 4:1 ratio. Data for **3h**: colorless solid; mp: 60-62 °C, R_f = 0.8 (hexane); ^1H NMR (400 MHz, $CDCl_3$) δ 7.23 - 7.10 (m, 3H), 6.79 (d, *J* = 6.8 Hz, 1H), 4.62 (dd, *J* = 4.4, 9.3 Hz, 1H), 3.00 (dd, *J* = 9.3, 12.7 Hz, 1H), 2.53 (s, 3H), 2.33 (dd, *J* = 4.4, 12.7 Hz, 1H), 1.65 (s, 3H); ^{13}C NMR (100 MHz, $CDCl_3$) δ 138.4, 134.0, 130.7, 127.7, 127.5, 126.3, 89.1, 83.4, 77.2, 47.1, 43.8, 20.7, 19.7; IR (neat): 3024, 2961, 1601, 1491, 1453, 1381, 1269, 1208, 1188, 1153, 1090, 1009, 954 cm^{-1}; HRMS (ESI): *m/z* calcd for $C_{15}H_{13}Cl_5Na$ $[M + Na]^+$ 392.9328; found: 392.9316.

Data for **4h**: colorless solid; mp:102-104 °C; R_f = 0.7 (hexane); ^1H NMR (400 MHz, $CDCl_3$) δ 7.23 - 7.16 (m, 3H), 6.82 - 6.75 (m, 1H), 4.14 (dd, *J* = 4.4, 9.3 Hz, 1H), 2.70 (dd, *J* = 9.3, 13.2 Hz, 1H), 2.51 (s, 3H), 2.43 (dd, *J* = 4.4, 13.2 Hz, 1H), 1.87 (s, 3H); ^{13}C NMR (100 MHz, $CDCl_3$) δ 138.0, 133.8, 132.1, 130.7, 127.9, 127.6, 126.5, 92.4, 83.7, 77.5, 45.6, 42.5, 20.9, 20.7; IR (neat): 2986, 2921, 1603, 1490, 1462, 1380, 1286, 1264, 1253, 1217, 1183, 1152, 1113, 1086, 1009, 974, 959 cm^{-1}; HRMS (ESI): *m/z* calcd for $C_{15}H_{13}Cl_5Na$ $[M + Na]^+$ 392.9328; found: 392.9311.

(1R^*,4R^*,5R^*,7S^*)-1,2,3,4,7-Pentachloro-5-(4-isopropylphenyl)-7-methylbicyclo[2.2.1]hept-2-ene (3i) and (1R^*,4R^*,5R^*,7R^*)-1,2,3,4,7-pentachloro-5-(4-isopropylphenyl)-7-methylbicyclo[2.2.1]hept-2-ene (4i):

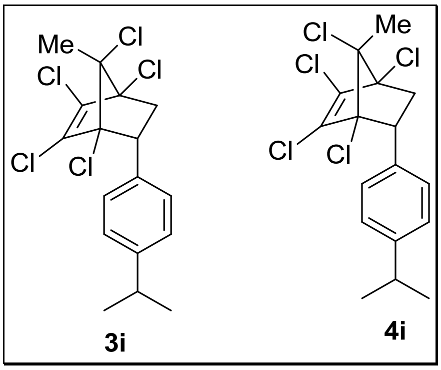

Following the same procedure as for **3a** starting from compound **1** (400 mg, 1.58 mmol) and 1-isopropyl-4-vinylbenzene, **2i** (238 mg, 1.63 mmol), compounds **3i** and **4i** (556 mg, 89%) were obtained in 93:7 ratio. Data for **3i**: colorless solid; mp: 56-58 °C; R_f = 0.8 (hexane); ^{1}H NMR (400 MHz, $CDCl_3$) δ 7.20 (d, J = 7.8 Hz, 2H), 7.05 (d, J = 8.3 Hz, 2H), 4.07 (dd, J = 4.4, 9.3 Hz, 1H), 2.98 (dd, J = 9.3, 12.7 Hz, 1H), 2.94 - 2.89 (m, 1H), 2.41 (dd, J = 4.2, 12.5 Hz, 1H), 1.65 (s, 3H), 1.26 (d, J = 7.3 Hz, 6H); ^{13}C NMR (100 MHz, $CDCl_3$) δ 148.7, 132.5, 130.7, 130.5, 128.9 (2C), 126.4 (2C), 88.6, 82.6, 77.3, 52.6, 42.1, 33.7, 23.95, 23.9, 19.8; IR (neat): 2956, 2928, 2872, 1601, 1550, 1513, 1450, 1419, 1381, 1362, 1269, 1256, 1184, 1151, 1105, 1089, 1058, 1009, 952 cm^{-1}; HRMS (ESI): *m/z* calcd for $C_{17}H_{17}Cl_5Na$ $[M + Na]^+$ 420.9641; found: 420.9634.

Data for **4i**: colorless liquid; R_f = 0.6 (hexane); ^{1}H NMR (400 MHz, $CDCl_3$) δ 7.21 (d, J = 8.3 Hz, 2H), 7.03 (d, J = 8.3 Hz, 2H), 3.65 (dd, J = 4.6, 9.0 Hz, 1H), 2.92 (td, J = 6.8, 13.4 Hz, 1H), 2.65 (dd, J = 9.1, 13.1 Hz, 1H), 2.50 (dd, J = 4.5, 13.1 Hz, 1H), 1.82 (s, 3H), 1.26 (d, J = 6.8 Hz, 6H); ^{13}C NMR (100 MHz, $CDCl_3$) δ 149.0, 132.3, 132.1, 131.8, 128.8 (2C), 126.5 (2C), 91.9, 83.1, 77.5, 51.2, 40.6, 33.7, 23.92, 23.88, 20.8; IR (neat): 2960, 2928, 2870, 1602, 1513, 1463, 1442, 1420, 1383, 1363, 1263, 1223, 1187, 1154, 1096, 1057, 1018, 980, 969 cm^{-1}; HRMS (ESI): *m/z* calcd for $C_{17}H_{18}Cl_5$ $[M + H]^+$ 396.9851 ; found: 396.9902.

4-((1R^*,4R^*,5R^*,7S^*)-1,4,5,6,7-Pentachloro-7-methylbicyclo[2.2.1]hept-5-en-2-yl)pyridine (3j):

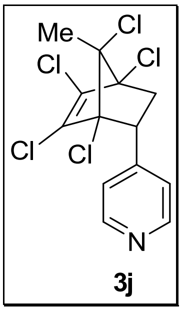

Following the same procedure as for **3a** starting from compound **1** (200 mg, 0.79 mmol) and 4-vinylpyridine, **2j** (174 mg, 0.95 mmol), compound **3j** (263 mg, 77%) was obtained as pale yellow solid; mp: 94-96 °C; R_f = 0.6 (25% EtOAc in hexane); ^{1}H NMR (400 MHz, $CDCl_3$) δ 8.57 (d, *J* = 5.4 Hz, 2H), 7.05 (d, *J* = 5.9 Hz, 2H), 4.05 (dd, *J* = 4.2, 9.0 Hz, 1H), 2.99 (dd, *J* = 9.3, 12.7 Hz, 1H), 2.39 (dd, *J* = 4.4, 12.7 Hz, 1H), 1.64 (s, 3H); ^{13}C NMR (100 MHz ,$CDCl_3$) δ 149.8 (2C), 144.5, 131.2, 130.1, 124.1 (2C), 88.4, 82.1, 77.4, 52.3, 41.1, 19.6; IR (neat) 2925, 2853, 1599, 1557, 1494, 1453, 1415, 1381, 1272, 1258, 1203, 1151, 1107, 1088, 1029, 1012, 950 cm^{-1}; HRMS (ESI): *m/z* calcd for $C_{13}H_{11}Cl_5N$ $[M + H]^+$ 357.9305; found: 357.9300.

(1*R,4*R**,5*R**,7*S**)-5-(2-Bromophenyl)-1,2,3,4,7-pentachloro-7-methylbicyclo[2.2.1]hept-2-ene (3k) and (1*R**,4*R**,5*R**,7*R**)-5-(2-bromophenyl)-1,2,3,4,7-pentachloro-7-methylbicyclo [2.2.1] hept-2-ene (4k):**

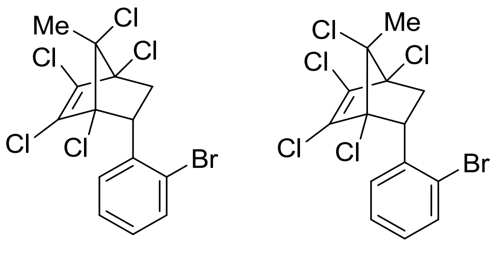

3k **4k**

Following the same procedure as for **3a** starting from compound **1** (200mg, 0.79 mmol) and 1-bromo-2-vinylbenzene, **2k** (174 mg, 0.95 mmol), compounds **3k** and **4k** (263 mg, 77%) were obtained in 4:1 ratio. Data for **3k**: colorless solid; mp: 78-80 °C, R_f = 0.8 (hexane); ^{1}H NMR (400 MHz, $CDCl_3$) δ 7.61 (dd, *J* = 1.0, 8.0 Hz, 1H), 7.27 - 7.24 (m, 1H), 7.18 - 7.12 (m, 1H), 6.83 (dd, *J* = 1.0, 7.8 Hz, 1H), 4.99 (dd, *J* = 4.4, 9.3 Hz, 1H), 3.04 (dd, *J* = 9.3, 12.7 Hz, 1H), 2.23 (dd, *J* = 4.6, 12.5 Hz, 1H), 1.63 (s, 3H); ^{13}C NMR (100 MHz, $CDCl_3$) δ 135.2, 133.4, 131.2, 130.6, 129.4, 129.2, 129.1, 127.6, 82.6, 77.4, 71.1, 50.0, 43.8, 19.5; IR (neat) 3030, 2921, 1602, 1473, 1435, 1384, 1259, 1184, 1155, 1097, 1033, 1009, 970 cm^{-1}; HRMS (APCI): calcd for $C_{14}H_{10}BrCl_5[M]^+$ 431.8409; found: 431.8483.

Data for **4k**: colorless solid; mp: 96-98 °C, R_f = 0.75 (hexane); ^{1}H NMR (400 MHz, $CDCl_3$) δ 7.61 (d, *J* = 7.8 Hz, 1H), 7.32 - 7.27 (m, 1H), 7.19 - 7.11 (m, 1H), 6.84 (d, *J* = 7.8 Hz, 1H), 4.54 (dd, *J* = 4.9, 9.3 Hz, 1H), 2.75 (dd, *J* = 9.3, 13.2 Hz, 1H), 2.33 (dd, *J* = 4.9, 13.2 Hz, 1H), 1.87 (s, 3H); ^{13}C NMR (100 MHz, $CDCl_3$) δ 134.9, 133.4, 132.5, 131.9, 129.7, 129.2, 127.8, 126.8, 92.3, 83.2, 77.1, 48.4, 42.5, 20.8; IR (neat) 2986, 2921, 1600, 1470, 1433, 1382, 1260, 1180, 1153, 1095, 1031, 1010, 968 cm^{-1}; HRMS (ESI): calcd for $C_{14}H_{10}BrCl_5Na$ $[M + Na]^+$ 454.8306; found: 454.8338.

(1*R,4*R**,5*R**,7*S**)-1,2,3,4,7-Pentachloro-5-(2,4-dichlorophenyl)-7-methylbicyclo[2.2.1]hept-2-ene (3l):**

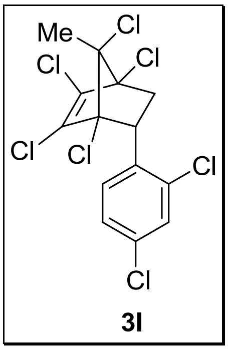

Following the same procedure as for **3a** starting from compound **1** (300 mg, 1.2 mmol) and 2,4-dichloro-1-vinylbenzene, **2l** (216 mg, 1.25 mmol), compound **3l** (354 mg, 70%) was obtained as colorless solid; mp: 138-140 °C; R_f = 0.8 (hexane); ^{1}H NMR (400 MHz, $CDCl_3$) δ 7.45 (d, *J* = 2.0 Hz, 1H), 7.22 (dd, *J* = 2.2, 8.6 Hz, 1H), 6.77 (d, *J* = 8.3 Hz, 1H), 4.92 (dd, *J* = 4.4, 9.3 Hz, 1H), 3.05 (dd, *J* = 9.3, 12.7 Hz, 1H), 2.20 (dd, *J* = 4.4, 12.7 Hz, 1H), 1.64 (s, 3H); ^{13}C NMR (100 MHz, $CDCl_3$) δ 136.8, 134.4, 132.3, 131.4, 129.85, 129.83, 127.3, 88.7, 82.4, 77.1, 47.2, 43.2, 43.5, 19.5; IR (neat): 2954, 2923, 2853, 1601, 1587, 1555, 1470, 1453, 1379, 1270, 1154, 1154, 1145, 1103, 1088, 1049, 1011, 953 cm^{-1}; HRMS (APCI): *m/z* calcd for $C_{14}H_{13}Cl_7N$ $[M + NH_4]^+$ 439.8868; found: 439.8897.

1-((1*R,4*R**,5*R**,7*S**)-1,4,5,6,7-Pentachloro-7-methylbicyclo[2.2.1]hept-5-en-2-yl)ethanone (3m)** and **1-((1*R**,4*R**,5*R**,7*R**)-1,4,5,6,7-pentachloro-7-methylbicyclo[2.2.1]hept-5-en-2-yl)ethanone (4m):**

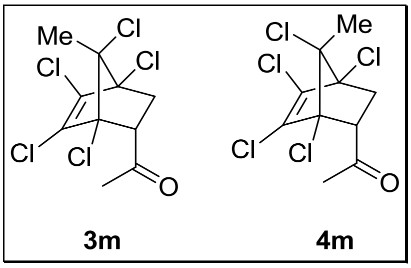

Following the same procedure as for **3a** starting from compound **1**(500 mg, 1.98 mmol) and methylvinyl ketone, **2m** (413 mg, 5.9 mmol), compounds **3m** and **4m** (610 mg, 95%) were obtained in 4:1 ratio. Data for **3m**: colorless solid; mp: 72-74 °C; R_f = 0.55 (5% EtOAc in hexane); ^{1}H NMR (400 MHz, $CDCl_3$) δ 3.85 (dd, *J* = 4.4, 8.3 Hz, 1H), 2.62 - 2.47 (m, 2H), 2.39 (s, 3H), 1.60 (s, 3H); ^{13}C NMR (125 MHz, $CDCl_3$) δ 203.5, 132.0, 128.0, 89.0, 79.6, 77.1, 58.8, 37.7, 31.7, 19.2; ; IR (neat): 2933, 2841, 1718, 1603, 1444, 1381, 1360, 1273, 1244, 1175, 1151, 1105, 983 cm^{-1}; HRMS (ESI): *m/z* calcd for $C_{10}H_9Cl_5ONa$ $[M + Na]^+$ 344.8964; found: 344.8955.

Data for **4m**: colourless solid; mp: 78-80 °C; R_f = 0.5 (5% EtOAc in hexane); ^{1}H NMR (400 MHz, $CDCl_3$) δ 3.42 (dd, *J* = 4.3, 8.5 Hz, 1H), 2.62 (dd, *J* = 3.9, 12.6 Hz, 1H), 2.40 (s, 3H),

2.17 (dd, J = 8.7, 12.5 Hz, 1H), 1.71 (s, 3H); ^{13}C NMR (125 MHz, CDCl$_3$) δ 202.1, 133.3, 129.0, 91.5, 80.0, 77.1, 56.9, 35.8, 31.5, 20.7; IR (neat): 2930, 2840, 1716, 1604, 1439, 1384, 1359, 1270, 1241, 1175, 1153, 1101, 982 cm^{-1}; HRMS (APCI): *m/z* calcd for $C_{10}H_9Cl_5ONa$ $[M + Na]^+$ 344.8964; found: 344.8957.

(1*R,4*R**,5*R**,7*S**)-1,4,5,6,7-Pentachloro-7-methylbicyclo[2.2.1]hept-5-ene-2-carbonitrile (3n):**

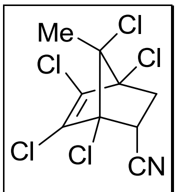

3n

Following the same procedure as for **3a** starting from compound **1** (200 mg, 0.79 mmol) and acrylonitrile, **2n** (167 mg, 3.16 mmol), compound **3n** (222 mg, 92%) was obtained as colorless solid; mp: 138-140 °C; R$_f$ = 0.8 (hexane); ^{1}H NMR (400 MHz, CDCl$_3$) δ 3.78 (dd, J = 4.0, 9.4 Hz, 1H), 2.96 (m, 1 H), 2.29 (m, 1H), 1.60 (s, 3H); ^{13}C NMR (125 MHz, CDCl$_3$) δ 132.2, 129.9, 116.8, 86.3, 77.4, 76.3, 41.5, 39.2, 19.1; IR (neat): 3023, 2250, 1602, 1448, 1383, 1275, 1234, 1156, 1110, 1095, 1046, 1015, 953 cm^{-1}; HRMS (ESI): *m/z* calcd for $C_9H_6Cl_5NNa$ $[M + Na]^+$ 327.8811; found: 327.8806.

Compound 3o, 4o:

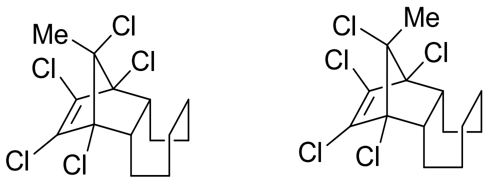

3o **4o**

Following the same procedure as for **3a** starting from compound **1** (500 mg, 1.98 mmol) and cyclooctene, **2o** (468 mg, 4.26 mmol), compounds **3o** and **4o** (582 mg, 81%) were obtained in 4:1 ratio. Data for **3o**: colorless solid; mp: 42-44 °C; R$_f$ = 0.85 (hexane); ^{1}H NMR (400 MHz, CDCl$_3$) δ 2.74 (dd, J = 2.1, 8.0 Hz, 2H), 1.90 (dt, J = 14.0, 3.7 Hz, 2H), 1.69-1.62 (m, 2H), 1.57 (s, 3H), 1.40-1.38 (m, 4H), 1.28-1.13 (m, 4H); ^{13}C NMR (125 MHz, CDCl$_3$) δ 130.1 (2C), 87.6, 81.9, 51.7 (2C), 30.3 (2C), 25.9 (2C), 22.6 (2C), 20.1 (2C); IR (neat): 2922, 2852, 1602, 1465, 1445, 1380, 1283, 1249, 1162, 1108, 1091, 992, 947 cm^{-1}; HRMS (ESI): (*m/z*) M^+ calcd for $C_{14}H_{18}Cl_5[M + H]^+$ 360.9851, found: 360.9902

Data for **4o**: colorless solid; mp: 112-114 °C; R$_f$ = 0.8 (hexane); ^{1}H NMR (400 MHz, CDCl$_3$) δ 2.35 (dd, J = 1.9, 7.3 Hz, 2H), 1.96-1.91 (m, 2H), 1.74-1.68 (m, 2H), 1.71 (s, 3H), 1.40-1.38 (m, 4H), 1.25-1.20 (m, 4H); ^{13}C NMR (125 MHz, CDCl$_3$) δ 131.5 (2C), 92.0, 82.4, 50.7 (2C), 30.3 (2C), 25.9 (2C), 23.3 (2C), 21.1 (2C); IR (neat): 3030, 1600, 1486, 1448, 1380,

1266, 1152, 1089, 1008, 954 cm^{-1}; HRMS (ESI): (*m/z*) M^+ calcd for $C_{14}H_{18}Cl_5$[M + H]$^+$ 360.9851, found: 360.9890

(1*R,4*R**,5*R**,7*S**)-1,2,3,4,7-Pentachloro-7-methyl-5-(prop-1-en-2-yl)bicyclo[2.2.1]hept-2-ene (3p):**

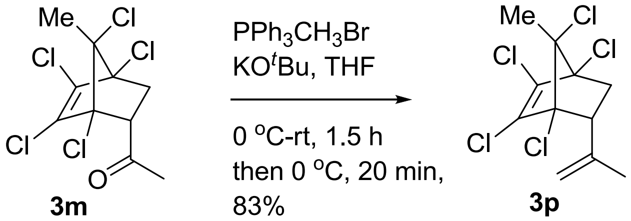

To the stirred suspension of methyltriphenylphosphonium bromide (443 mg, 1.24 mmol) in THF (3 mL) was added KO*t*Bu (135 mg, 1.209 mmol) at 0 °C and the resulting ylide was stirred for 10 min. then allowed to warmed to rt and stirred for 1.5 h. Then reaction mixture was cooled to -5 °C (ice salt mixture) and to this was added a solution of ketone **3m** (200 mg, 0.62 mmol) in THF (2 mL). Completion of starting material was monitored by tlc, after 20 min, 3 mL of water was added and diluted with EtOAc (30 mL), organic phase was separated, aqueous phase was extracted with EtOAc (25mL x 3) combined organic phases was washed with water (25 mL), brine (20 mL), dried over Na_2SO_4, and concentrated in *vacuo*. The resulting crude was purified by silica gel column chromatography using hexane as eluent furnished diene **3p** (165 mg, 83%) as colorless liquid, R_f = 0.9 (hexane); ^{1}H NMR (400 MHz, $CDCl_3$) δ 5.03 (s, 1H), 4.74 (s, 1H), 3.52 (dd, J = 4.2, 9.0 Hz, 1H), 2.72 (dd, J = 9.0, 12.5 Hz, 1H), 2.11 (dd, J = 4.2, 12.5 Hz, 1H), 1.87 (s, 3H), 1.60 (s, 3H), ^{13}C NMR (100 MHz, $CDCl_3$) δ 140.4, 130.5, 130.2, 115.6, 88.8, 81.6, 76.9, 53.1, 40.6, 23.9, 19.6; IR (neat): 3084, 2994, 2972, 2942, 1644, 1601, 1446, 1379, 1269, 1212, 1152, 1107, 1091, 1046, 1010, 949 cm^{-1}; HRMS (ESI): (*m/z*) M^+ calcd for $C_{11}H_{11}Cl_5Na$ [M + Na]$^+$ 340.9201, found: 340.9256.

General procedure for the KO_2-mediated synthesis of tetrachloro-substituted 3-methylenenortricyclenes 5:

To a stirred solution of **3a** & **4a** (1.1 g, 3.09 mmol) in DMSO (31 mL) was added KO_2 (660 mg, 9.26 mmol) in portions at ~15 °C (ice-water bath) under argon atmosphere and then allowed to warm to room temperature and then heated at 60 °C, reaction mixture turned deep wine-red color. After completion of reaction 6 h (monitored by TLC) 25 mL of water was added drop wise to destroy the extra KO_2. The mixture was then diluted with EtOAc (70 mL), organic phase was separated and aqueous phase was extracted with EtOAc (60 mL x 3). The combined organic phases were washed with water (50 mL), brine (50 mL), dried over Na_2SO_4, filtered and concentrated afforded the crude product. Column chromatography (hexane) provided compound **5a** as a colourless solid (837 mg, 85%)

^{1}H NMR, ^{13}C NMR data of the nortricyclenes 5

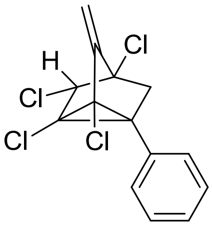

5a

R$_f$ = 0.75 (Hexane), mp: 80-82 °C; ^{1}H NMR (400 MHz, $CDCl_3$) δ 7.44-7.26 (m, 5H) 5.42 (s, 1H) 5.33 (s, 1H) 4.26 (s, 1H) 2.94 (d, J = 10.7 Hz, 1H) 2.31 (d, J = 10.7 Hz, 1H); ^{13}C NMR (125 MHz, $CDCl_3$) δ 147.2, 130.8, 130.1 (2C), 128.7 (2C), 128.5, 104.2, 71.4, 65.9, 57.7, 54.0, 48.0, 43.02; IR (KBr): 3044, 2977, 2942, 1691, 1603, 1500, 1446, 1368, 1279, 1233, 1177, 1119, 1092, 1047, 1023 cm^{-1}; EI-HRMS: (m/z) M$^+$ calculated for $C_{14}H_{10}Cl_4$: 317.9537; found: 317.9538.

Compound 5b:

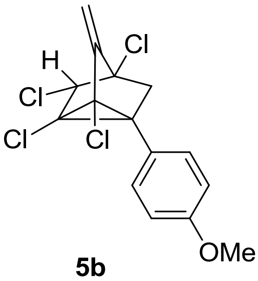

5b

Following the same procedure as for **5a** starting from compound **3b** (30 mg, 0.077mmol), compound **5b** (23.8 mg, 88%) was obtained as colorless solid. mp: 42-44 °C; R$_f$ = 0.5 (5% CH_2Cl_2 in hexane); ^{1}H NMR (400 MHz, $CDCl_3$) δ 7.30 (d, J = 8.8 Hz, 2H), 6.93 (d, J = 8.8 Hz, 2H), 5.40 (s, 1H), 5.31 (s, 1H), 4.24 (s, 1H), 3.82 (s, 3H), 2.90 (d, J = 10.7 Hz, 1H), 2.26 (d, J = 10.5 Hz, 1H); ^{13}C NMR (125 MHz, $CDCl_3$) δ 159.7, 147.3, 131.3 (2C), 122.7, 114.3 (2C), 104.0, 71.5, 66.0, 56.0, 55.4, 54.2, 48.1, 42.6; IR (neat): 2960, 2836, 1691, 1610, 1461, 1371, 1298, 1275, 1248, 1175, 1122, 1110, 1091, 1044, 1028, 999, 954 cm^{-1}; EI-HRMS: (m/z) M$^+$ calculated for $C_{15}H_{12}Cl_4O$: 347.9642; Found: 347.9652.

Compounds 5b and 6a:

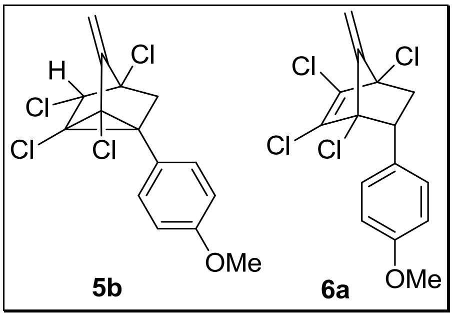

Following the same procedure as for **5a** starting from compound **4b** (22 mg, 0.056 mmol), compounds **5b** and **6a** (17 mg, 86%) were obtained as inseparable mixture in 2:1 (**5b**:**6a**). NMR data for **5b:** R_f= 0.5 (5% CH_2Cl_2 in hexane); ^{1}H NMR (400 MHz, $CDCl_3$) δ 7.30 (d, *J* = 8.8 Hz, 2H), 6.93 (d, *J* = 8.8 Hz, 2H), 5.40 (s, 1H), 5.31 (s, 1H), 4.24 (s, 1H), 3.82 (s, 3H), 2.90 (d, *J* = 10.7 Hz, 1H), 2.27 (d, *J* = 10.7 Hz, 1H); ^{13}C NMR (125 MHz, $CDCl_3$) δ 159.7, 147.3, 131.3 (2C), 122.7, 114.3 (2C), 104.0, 71.5, 66.0, 56.0, 55.4, 54.2, 48.1, 42.6; NMR data for **6a**: ^{1}H NMR (400 MHz, $CDCl_3$) δ 7.03 (d, *J* = 8.8 Hz, 2H), 6.86 (d, *J* = 8.8 Hz, 2H), 4.94 (d, *J* = 1.48 Hz, 1H), 4.91 (d, *J* = 1.48 Hz, 1H), 3.60 (dd, *J* = 9.6, 4.9 Hz, 1H), 2.63 (dd, *J* = 12.3, 9.6 Hz, 1H), 2.45 (dd, *J* = 12.3, 4.9 Hz, 1H), ^{13}C NMR (125 MHz, $CDCl_3$) δ 159.5, 157.7, 131.8, 131.7, 129.8 (2C), 127.8, 113.8 (2C), 93.8, 70.8, 58.0, 55.3, 55.0, 44.7, IR (neat): 3001, 2955, 2836, 1692, 1611, 1585, 1516, 1461, 1297, 1268, 1250, 1178, 1031, 1000, 959 cm^{-1}; HRMS (ESI): *m/z* calcd for $C_{15}H_{13}Cl_4O$ $[M + H]^+$ 350.9691, found: 350.9674.

Compound 5c:

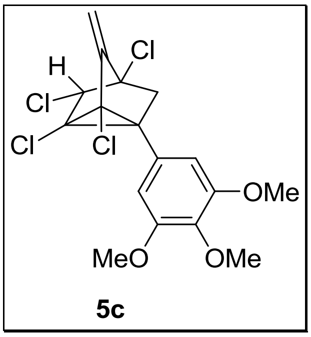

Following the same procedure as for **5a** starting from compounds **3c** and **4c** (30 mg, 0.067 mmol), compound **5c** (23 mg, 84%) was obtained at rt as colorless solid (in a separate experiment 5 mol% of BHT was used for the same scale yield of nortricyclene 94%). R_f = 0.5 (5% EtOAc in hexane); mp : 92-94 ^oC; ^{1}H NMR (400 MHz, $CDCl_3$) δ 6.55 (s, 2H), 5.43 (d, *J* = 0.7 Hz, 1H), 5.34 (s, 1H), 4.26 (s, 1H), 3.88 (s, 3H), 3.88(s, 3H), 3.87 (s, 3H), 2.93 (d, *J* = 10.7 Hz, 1H), 2.30 (d, *J* = 10.7 Hz, 1H); ^{13}C NMR (100 MHz, $CDCl_3$) δ 153.4, 147.0 (2C), 138.2, 126.0, 107.0 (2C), 104.2, 71.3, 65.8, 60.9, 57.8, 56.2 (2C), 54.1, 47.9, 43.2; IR (neat): 2984, 2936, 2828, 1696, 1585, 1508, 1456, 1412, 1386, 1328, 1263, 1239, 1121,

1005, 951 cm^{-1}; HRMS(ESI): *m/z* calcd for $C_{17}H_{20}Cl_4NO_3$ [M + NH$_4$]$^+$ 428.0168, found: 428.0145.

Compound 5d:

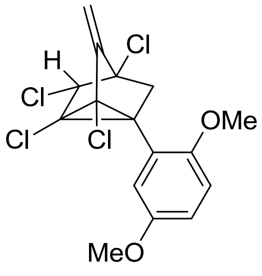

5d

Following the same procedure as for **5a** starting from compounds **3d** and **4d** (38 mg, 0.091 mmol), compound **5d** (27 mg, 78%) was obtained as colorless liquid. R_f = 0.45(2% EtOAc in hexane); ^{1}H NMR (400 MHz, $CDCl_3$) δ 6.91 (d, *J* = 2.3 Hz, 1H), 6.81-6.71 (m, 2H), 5.31 (s, 1H), 5.22 (s, 1H), 4.14 (s, 1H), 3.70 (s, 6H), 2.81 (d, *J* = 10.4 Hz, 1H), 2.30 (d, *J* = 10.4 Hz, 1H); ^{13}C NMR (100 MHz, $CDCl_3$) δ 153.3, 153.2, 147.4, 120.0, 117.3, 114.5, 111.8, 103.5, 71.4, 66.1, 57.3, 55.9, 55.8, 54.0, 45.3, 41.0; IR (neat): 2951, 2833, 1692, 1611, 1588, 1503, 1462, 1462, 1427, 1268, 1219, 1168, 1046, 1025, 998, 964 cm^{-1}; HRMS (ESI): *m/z* calcd for $C_{16}H_{18}Cl_4NO_2$ [M + NH$_4$]$^+$ 398.0062; found: 398.0098.

Compound 5f:

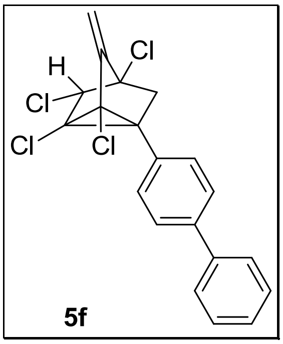

5f

Following the same procedure as for **5a** starting from compounds **3f** and **4f** (60 mg, 0.138 mmol), compound **5f** (47 mg, 86%) was obtained at rt as colorless solid (in a separate experiment 5 mol% of BHT was used for the same scale yield of nortricyclene 91%). R_f = 0.5 (hexane); mp :106-108 °C; ^{1}H NMR (400 MHz, $CDCl_3$) δ 7.62 (d, *J* = 8.2 Hz, 2H), 7.58 (d, *J* = 7.8 Hz, 2H), 7.47- 7.42 (m, 4H), 7.37-7.34 (m, 1H), 5.43 (s, 1H), 5.35 (s, 1H), 4.28 (s, 1H), 2.97 (d, *J* = 10.6, 1H), 2.33(d, *J* = 10.7 Hz, 1H); ^{13}C NMR (100 MHz, $CDCl_3$) δ 147.1, 141.3, 140.5, 130.3 (2C), 129.7, 128.8 (2C), 127.6, 127.4 (2C), 127.2 (2C), 104.2, 71.4, 66.0, 57.8, 54.1, 47.8, 42.8; IR (neat): 3030, 1691, 1600, 1528, 1447, 1398, 1234, 1193, 1122, 998 cm^{-1}; HRMS (APCI): *m/z* calcd for $C_{20}H_{14}Cl_4K$ [M + K]$^+$ 434.9457, found: 434.9448.

Compound 5g:

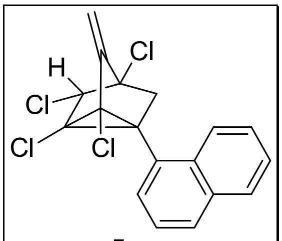

5g

Following the same procedure as for **5a** starting from compounds **3g** and **4g** (40 mg, 0.098 mmol), compound **5g** (30.5mg, 83%) was obtained as colorless solid. R_f = 0.45 (hexane); mp : 82-84 °C; ^{1}H NMR (500 MHz, $CDCl_3$) δ 7.89-7.85 (m, 2H), 7.62 (d, *J* = 7.0 Hz, 1H), 7.55-7.48 (m, 4H), 5.55 (s, 1H), 5.48 (s, 1H), 4.37 (s, 1H), 3.01 (d, *J* = 7.6 Hz, 1H), 2.54 (d, *J* = 7.6 Hz, 1H); ^{13}C NMR (125 MHz, $CDCl_3$) δ 147.2, 134.1. 132.1, 129.9, 129.5, 129.0, 127.2, 126.5, 126.1, 125.4, 124.8, 104.8, 71.7, 66.1, 57.3, 54.1, 47.5, 42.8; IR (KBr): 3041, 1690, 1593, 1549, 1508, 1447, 1400, 1353, 1247, 1235, 1209, 1123, 997 cm^{-1}; HRMS (APCI): *m/z* calcd for $C_{18}H_{13}Cl_4$ $[M + H]^+$ 370.9742, found: 370.9740.

Compound 5h:

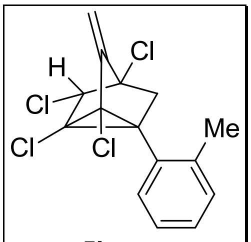

5h

Following the same procedure as for **5a** starting from compounds **3h** (25 mg, 0.067 mmol), compound **5h** (16.7 mg, 75%) was obtained as colorless liquid. R_f =0.8 (hexane); ^{1}H NMR (400 MHz, $CDCl_3$) δ 7.39 (d, *J* = 7.7 Hz, 1H), 7.29-7.17 (m, 3H), 5.44 (s, 1H), 5.35 (s, 1H), 4.26 (s, 1H), 2.89 (d, *J* = 10.8 Hz, 1H), 2.38 (s, 3H), 2.34 (d, *J* = 10.8, 1H), ^{13}C NMR (100 MHz, $CDCl_3$) δ 147.1, 138.9, 131.4, 131.0, 129.0, 128.8, 125.9, 104.3, 71.5, 65.8, 57.0, 54.0, 46.2, 43.3, 19.7; IR (neat): 2950, 2926, 2855, 1692, 1511, 1495, 1452, 1381, 1273, 1236, 1122, 998, 946 cm^{-1}; HRMS (APCI): *m/z* calcd for $C_{15}H_{13}Cl_4$ $[M + H]^+$ 334.9742, found: 334.9744

Compound 5h and **6b**:

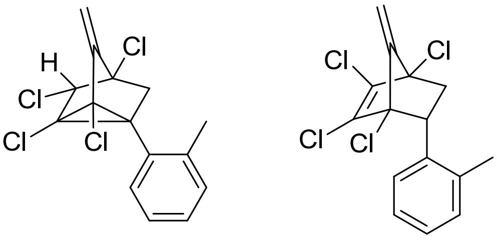

5h **6b**

Following the same procedure as for **5a** starting from compounds **4h** (20 mg, 0.054 mmol), compound **5h** and **6b** (11.3 mg, 63% combined yield) were obtained inseparable mixture in 2:3 (**5h:6b**) ratio as colorless liquid. $R_f = 0.5$ (hexane); NMR data of **5h** : ^{1}H NMR (400 MHz, $CDCl_3$) δ 7.39 (d, $J = 7.7$ Hz, 1H), 7.29-7.17 (m, 3H), 5.44 (s, 1H), 5.35 (s, 1H), 4.26 (s, 1H), 2.89 (d, $J = 10.8$ Hz, 1H), 2.38 (s, 3H), 2.34 (d, $J = 10.8$ Hz, 1H), ^{13}C NMR (100 MHz, $CDCl_3$) δ 147.1, 138.9, 131.4, 131.0, 129.0, 128.8, 125.9, 104.3, 71.5, 65.8, 57.0, 54.0, 46.2, 43.3, 19.7; NMR data of **6b:** 7.20-7.15 (m, 3H), 6.83-6.80 (m, 1H), 4.96 (d, $J = 1.5$ Hz, 1H), 4.92 (d, $J = 1.5$ Hz, 1H), 4.09 (dd, $J = 9.6$, 5 Hz, 1H), 2.68 (dd, $J = 12.2$, 9.7 Hz, 1H), 2.48 (s, 3H), 2.43-2.40 (m, 1H); ^{13}C NMR (100 MHz, $CDCl_3$) δ 158, 138.1, 134.7, 133.1, 132.5, 130.7, 127.7, 127.16, 126.3, 93.4, 70.7, 60.6, 49.8, 46.4, 20.7; IR (neat): 3023, 2955, 1692, 1587, 1492, 1454, 1380, 1268, 1235, 1189, 1141, 1030, 1000, 958 cm^{-1}; HRMS (APCI): *m/z* calcd for $C_{15}H_{16}NCl_4$ $[M + NH_4]^+$ 350.0037, found: 350.0011

Compound 5i:

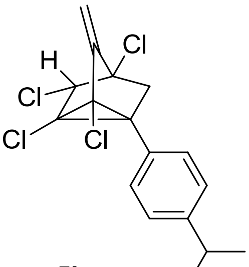

5i

Following the same procedure as for **5a** starting from compound **3i** and **4i** (50mg, 0.125 mmol), compound **5i** (37.2 mg, 85%) was obtained as colorless liquid (in a separate experiment 5 mol% of BHT was used for the same scale yield of nortricyclene 89%). $R_f = 0.6$ (hexane); ^{1}H NMR (400 MHz, $CDCl_3$) δ 7.28 (m, 4H), 5.40 (s, 1H), 5.32 (s, 1H), 4.25 (s, 1H), 2.96 - 2.87 (m, 2H), 2.28 (d, $J = 10.8$ Hz, 1H), 1.26 (d, $J = 6.8$ Hz, 6H); ^{13}C NMR (100 MHz, $CDCl_3$) δ 149.1, 147.3, 129.8, 128.0, 126.7, 103.9, 71.4, 65.9, 57.8, 54.1, 47.9, 42.8, 33.9, 23.91, 23.88; IR (neat): 2961, 2927, 2869, 1690, 1512, 1459, 1448,1406, 1365, 1278, 1267, 1235, 1120, 1061, 1018, 998 cm^{-1}; HRMS (APCI): *m/z* calcd for $C_{17}H_{17}Cl_4[M + H]^+$ 363.0055, found: 363.0042.

Compound 5j :

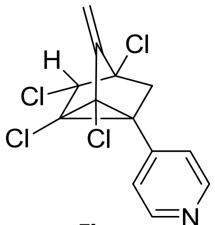

5j

Following the same procedure as for **5a** starting from compound **3j** (24 mg, 0.067 mmol), compound **5j** (12.7 mg, 60%) and **7** (3.8 mg, 16%) were obtained at rt as colorless liquids. R_f =0.45 (20% EtOAc in hexane); ^{1}H NMR (400 MHz, $CDCl_3$) δ 8.66 (dd, *J* = 4.5, 1.6 Hz, 2H), 7.33 (dd, *J* = 4.5, 1.7 Hz, 2H), 5.46 (d, *J* = 1 Hz, 1H), 5.37 (s, 1H), 4.27 (s, 1H), 2.98 (d, *J* = 10.6 Hz, 1H), 2.34 (d, *J* = 10.6 Hz, 1H); ^{13}C NMR (100 MHz, $CDCl_3$) δ 150.1, 146.3, 139.6, 124.4, 105.0, 77.2, 71.0, 65.6, 57.6, 54.0, 46.4, 41.7; IR (neat): 3030, 2925, 2854, 1692, 1598, 1550, 1500, 1448, 1410, 1281, 1237, 1123, 1071, 999 cm^{-1}; HRMS (APCI): *m/z* calcd for $C_{13}H_{10}Cl_4ON[M + H]^+$ 321.9538, found: 321.9523.

Compound 7:

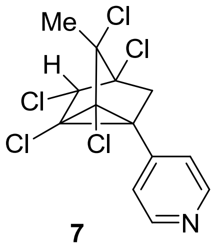

7

NMR data for **7:** R_f = 0.5 (20% EtOAc in hexane); ^{1}H NMR (400 MHz, $CDCl_3$) δ 8.67 (d, *J* = 6.0 Hz, 2H), 7.31 (dd, *J* = 4.5, 1.5 Hz, 2H), 4.51 (s, 1H), 3.01 (d, *J* = 11.3 Hz, 1H), 2.96 (d, *J* = 11.6 Hz, 1H), 1.77 (s, 3H); ^{13}C NMR (100 MHz, $CDCl_3$) δ 150.2 (2C), 139.0, 124.8 (2C), 78.8, 77.2, 71.0, 68.6, 60.4, 54.9, 45.4, 39.9, 29.7, 21.0; IR (neat) 3031, 2924, 2853, 1690, 1599, 1552, 1451, 1411, 1385, 1271, 1226, 1180, 1073, 1046, 1008 cm^{-1}; HRMS (APCI): *m/z* calcd for $C_{13}H_{10}Cl_4ON$ $[M + H]^+$ 321.9538, found: 321.9534.

Compound 5k:

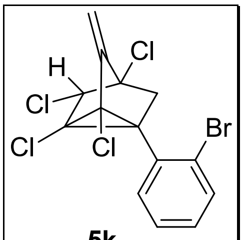

5k

Following the same procedure as for **5a** starting from compounds **3k** and **4k** (25 mg, 0.057 mmol), compound **5k** (64%) was obtained as colorless solid. R_f = 0.5 (hexane); mp: 78-80 ^{o}C; ^{1}H NMR (400 MHz, $CDCl_3$) δ 7.62 (dd, *J* = 7.9, 1.2 Hz, 1H), 7.53 (dd, *J* = 7.7, 1.7 Hz, 1H), 7.35 (td, *J* = 7.6, 1.3 Hz, 1H), 7.23 (td, *J* = 7.6, 1.7 Hz, 1H), 5.45 (s, 1H), 5.36 (s, 1H), 4.25 (s, 1H), 2.82 (d, *J* = 10.6 Hz, 1H), 2.78 (d, *J* = 10.6 Hz, 1H); ^{13}C NMR (100 MHz, $CDCl_3$) δ 146.4, 133.7, 132.8, 131.0, 130.2, 127.4, 125.6, 104.7, 71.5, 65.6, 56.6, 54.4, 44.7, 43.9; IR (neat): 3023, 2955, 1694, 1582, 1477, 1442, 1428, 1400, 1277, 1233, 1178, 1119, 1065, 1022, 997 cm^{-1}; HRMS (APCI): *m/z* calcd for $C_{14}H_{10}BrCl_4$ $[M + H]^+$ 396.8720, found: 396.8773.

Compound 5l:

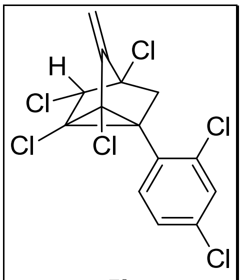

5l

Following the same procedure as for **5a** starting from compound **3l** (50 mg, 0.117 mmol), compound **5l** (40.2 mg, 78%) was obtained as colorless liquid. R_f = 0.6 (hexane); ^{1}H NMR (400 MHz, $CDCl_3$) δ 7.47-7.44 (m, 2H), 7.31-7.26 (m, 1H), 5.45 (d, *J* = 0.7 Hz, 1H), 5.36 (s, 1H), 4.24 (s, 1H), 2.83(d, *J* = 10.6 Hz, 1H), 2.58 (d, *J* = 10.6 Hz, 1H); ^{13}C NMR (100 MHz, $CDCl_3$) δ 146.2, 136.9, 135.5, 133.2, 130.2, 127.8, 127.3, 104.8, 71.2, 65.6, 56.8, 54.1, 44.6, 42.1; IR (neat): 3022, 2950, 1693, 1589,1554, 1482, 1448, 1385, 1281, 1234, 1124, 1106, 1072, 1001, 950 cm^{-1}; HRMS(ESI): *m/z* calcd for $C_{14}H_9Cl_6$ $[M + H]^+$ 388.8806, found: 388.8798.

Compound 5m:

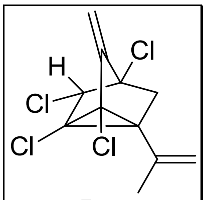

5m

Following the same procedure as for **5a** starting from compound **2p** (40 mg, 0.125 mmol) compound **5m** (28.1 mg, 80%) was obtained as colorless liquid. R_f = 0.85 (hexane); ^{1}H NMR (400 MHz, $CDCl_3$) δ 5.35 (s, 1H), 5.28-5.21 (m, 3H), 4.14 (s, 1H), 2.81 (d, *J* = 10.8 Hz, 1H), 2.15 (d, *J* = 10.8 Hz, 1H), 1.89 (s, 3H); ^{13}C NMR (100 MHz, $CDCl_3$) δ 147.3, 134.7, 119.0,

103.8, 71.4, 65.7, 57.1, 53.2, 45.2, 43.8, 20.8; IR (neat): 2947, 2923, 2855, 1692, 1648, 1527, 1501, 1451, 1381, 1271, 1234, 999, 904 cm^{-1}; HRMS (APCI): (*m/z)* M^+ calcd for $C_{11}H_{14}Cl_4N[M + NH_4]^+$ 299.9880, found: 299.9885.

Compound 6c:

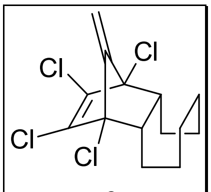

6c

Following the same procedure as for **5a** starting from compounds **3o** and **4o** (40 mg, 0.124 mmol), compound **6c** (34.6 mg, 89%) was obtained at 100 °C. R_f = 0.95 (hexane); mp: 116-118 °C; ^{1}H NMR (400 MHz, $CDCl_3$) δ 4.83 (s, 2H), 2.34 (dd, *J* = 7.3, 2.6 Hz, 2H), 1.95 (dd, *J* = 10.7, 4.4 Hz, 2H), 1.71 - 1.67 (m, 2H), 1.38-1.37 (m, 4H), 1.25-1.23 (m, 4H); ^{13}C NMR (100 MHz, $CDCl_3$) δ 157.8 (2C), 131.9 (2C), 92.9, 75.6, 55.0 (2C), 30.2 (2C), 25.9 (2C), 22.9 (2C); IR (KBr): 2953, 2923, 2850, 1702, 1586, 1463, 1442, 1383, 1297, 1279, 1244, 1202, 1156, 1035, 999 cm^{-1}; EI-HRMS: (m/z) M^+ calcd for $C_{14}H_{16}C_{14}$: 324.0006; found : 324.0007.

Compound 5n:

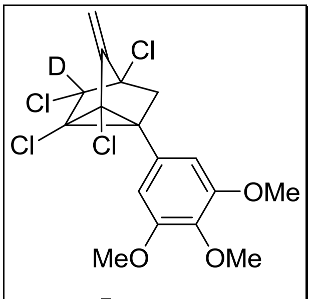

5n

Following the same procedure as for **5a** starting from compounds **3c** and **4c** (24 mg, 0.053 mmol), compound **5n** (16.7 mg, 77%) was obtained instead of DMSO, DMSO-d_6 was used as solvent and at room temperature. R_f = 0.5 (5% EtOAc in hexane); mp : 92-94 °C; ^{1}H NMR (400 MHz, $CDCl_3$) δ 6.56 (s, 2H), 5.44 (s, 1H), 5.35 (s, 1H), 3.88 (s, 6H), 3.87 (s, 3H), 2.94 (d, *J* = 10.8 Hz, 1H), 2.31 (d, *J* = 10.8 Hz, 1H); ^{13}C NMR (100 MHz, $CDCl_3$) δ 153.4, 147.1, 138.3, 126.0, 107.1, 104.2, 70.9 (triplet, C_5 attached to D), 65.7, 60.9, 57.7, 56.2, 54.1, 47.9, 43.2; IR(neat) 2937, 2830, 1693, 1585, 1509, 1453, 1413, 1388, 1300, 1263, 1237, 1125, 1008, 947 cm^{-1}; HRMS (ESI): *m/z* calcd for $C_{17}H_{19}DCl_4NO_3$ $[M + NH_4]^+$ 429.0231, found: 429.0210.

General procedure for the acylation reaction of nortricyclenes and ^{1}H NMR, ^{13}C NMR data of products of acylation reaction of nortricyclens:

1-((1*R,4*R**,5*R**,7*S**)-1,3,4,5,7-Pentachloro-5-phenylbicyclo[2.2.1]hept-2-en-2-yl)propan-2-one (8a):**

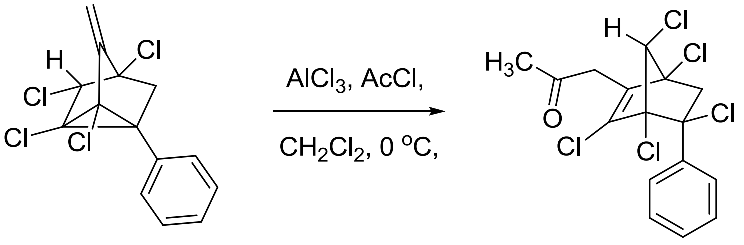

5a **8a**

To a stirred suspension of $AlCl_3$ (83 mg, 0.62 mmol) in CH_2Cl_2 (3 mL) was added acetyl chloride (73 mg, 0.93 mmol) at 0 °C and the resulting mixture was stirred for 10 min. Then a solution of **5a** (100 mg, 0.31 mmol) in CH_2Cl_2 (2 mL) was added. After being stirred for 5 min. 1 mL of dil. HCl was added drop wise and diluted with CH_2Cl_2 (25 mL). The organic phase was separated and aqueous phase was extracted with CH_2Cl_2 (30 mL x 3). The combined organic phases were washed with water (20 mL), brine (20 mL), dried over Na_2SO_4 and concentrated *in vacuo*. The resulting crude was purified over silica gel column chromatography with 5% EtOAc in Hexane, which afforded product **8a** (93 mg, 76%); R_f = 0.5 (5% EtOAc in Hexane); mp: 134-136 °C; ^{1}H NMR (400 MHz, $CDCl_3$) δ 7.67 (dd, *J* = 2.0, 7.3 Hz, 2H), 7.35 - 7.26 (m, 3H), 4.32 (d, *J* = 2.0 Hz, 1H), 3.97 (dd, *J* = 1.5, 13.7 Hz, 1H), 3.52 (d, *J* = 4.9 Hz, 2H), 3.37 (d, *J* = 13.7 Hz, 1H), 2.26 (s, 3H); ^{13}C NMR (100 MHz, $CDCl_3$) δ 202.7, 139.2, 138.9, 134.9, 128.4, 128.2, 1279, 80.9, 78.2, 75.1, 71.1, 48.5, 41.8, 30.2; IR (Neat) 2980, 1719, 1692, 1679, 1659, 1630, 1549, 1512, 1499, 1452, 1390, 1354, 1323, 1288, 1264, 1164, 1142, 1104, 1063, 1025, 995 cm^{-1}; HRMS (ESI): *m/z* calcd for $C_{16}H_{13}Cl_5OK$ $[M + K]^+$ 436.9017; Found: 436.9003.

1-((1*R**,4*R**,5*R**,7*S**)-5-(4'-Acetylbiphenyl-4-yl)-1,3,4,5,7-pentachlorobicyclo[2.2.1]hept-2-en-2-yl)propan-2-one, 8c:

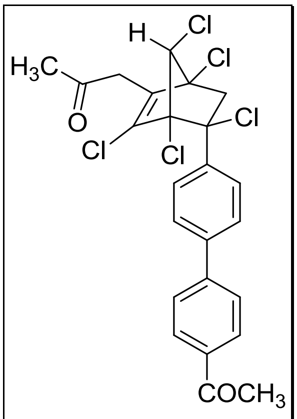

8c

Following the same procedure as for **8a** starting from compound **5f** (20 mg, 0.05 mmol), compound **8c** (17 mg, 68%) was obtained as pale yellow liquid. R_f = 0.5 (30% EtOAc in Hexane); ^{1}H NMR (400 MHz, $CDCl_3$) δ 8.03 (d, *J* = 8.3 Hz, 2H), 7.82 (d, *J* = 8.8 Hz, 2H), 7.69 (d, *J* = 8.3 Hz, 2H), 7.59 (d, *J* = 8.8 Hz, 2H), 4.34 (d, *J* = 1.5 Hz, 1H), 4.05 (dd, *J* = 1.5, 13.7 Hz, 1H), 3.57 (s, 2H), 3.41 (d, *J* = 13.7 Hz, 1H), 2.65 (s, 3H), 2.29 (s, 3H); ^{13}C NMR (100 MHz, $CDCl_3$) δ 202.9, 197.7, 144.5, 139.6, 139.3, 139.2, 136.1, 134.7, 128.9, 128.8, 127.1, 126.6, 80.9, 78.2, 74.9, 71.1, 48.6, 41.9, 30.2, 26.7; IR (neat) 2923, 2852, 1721, 1680, 1604, 1417, 1397, 1358, 1323, 1266, 1185, 1165, 1146, 1109, 1024, 1002, 957 cm^{-1}; HRMS (ESI): *m/z* calcd for $C_{24}H_{19}Cl_5O_2Na$ $[M + Na]^+$ 538.9696; Found: 538.9662.

2-((1*R**,4*R**,5*R**,7*S**)-1,3,4,5,7-Pentachloro-5-phenylbicyclo[2.2.1]hept-2-en-2-yl)-1-phenylethanone (8b):

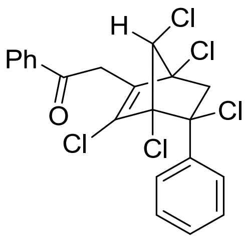

8b

Following the same procedure as for **8a** starting from compound **5a** (45 mg, 0.14 mmol), compound **8b** (36 mg, 61%) instead of acetyl chloride, benzoyl chloride was used, obtained as colorless liquid; R_f = 0.5 (5% EtOAc in Hexane); ^{1}H NMR (400 MHz, $CDCl_3$) δ 8.02 - 7.96 (m, 2H), 7.79 - 7.72 (m, 2H), 7.68 - 7.59 (m, 1H), 7.56 - 7.48 (m, 2H), 7.32 - 7.24 (m, 3H), 4.40 (d, *J* = 1.5 Hz, 1H), 4.18 (dd, *J* = 1.5, 13.7 Hz, 1H), 4.10 (d, *J* = 6.4 Hz, 2H), 3.43 (d, *J* = 13.7 Hz, 1H); ^{13}C NMR (100 MHz, $CDCl_3$) δ 194.5, 139.5, 139.3, 135.9, 135.1, 133.9, 128.9, 128.4, 128.3, 127.9, 81.1, 78.3, 75.2, 71.3, 48.7, 37.6; IR (neat) 2972, 2820, 1759,

1684, 1631, 1596, 1549, 1512, 1499, 1446, 1330, 1281, 1257, 1217, 1180, 1105, 1029, 989 cm^{-1}; HRMS (ESI): *m/z* calcd for $C_{21}H_{15}Cl_5OK$ $[M + K]^+$ 498.9173; Found: 498.9146.

Copies of ^{1}H NMR, ^{13}C NMR spectra of DA adducts 3/4 :

^{1}H NMR (400 MHz) and ^{13}C NMR (100 MHz) of 3a in $CDCl_3$:

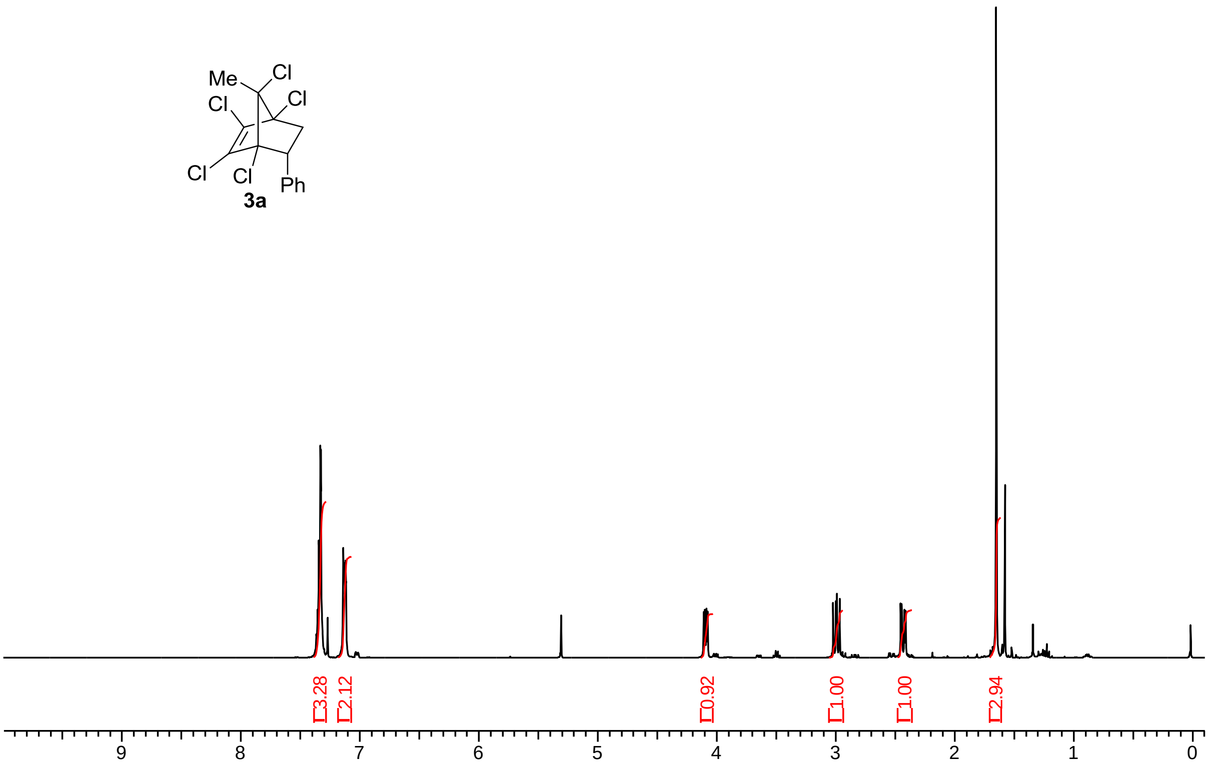

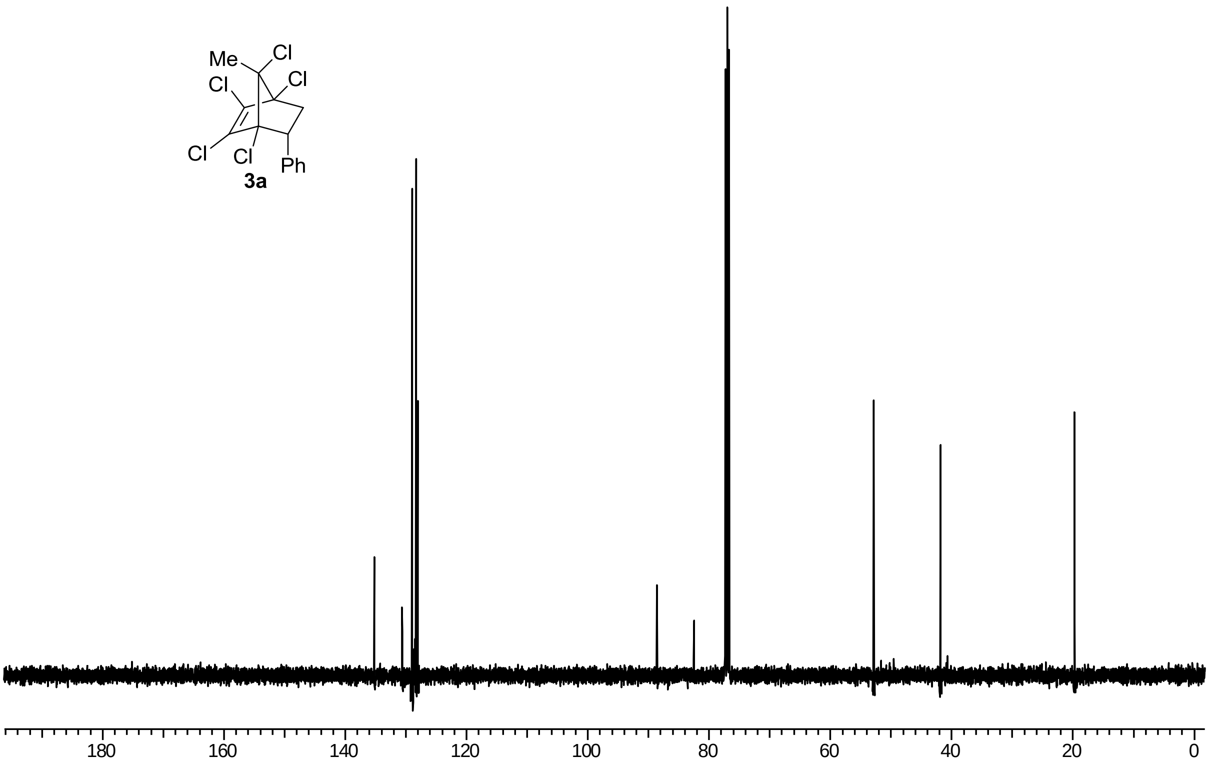

^{1}H NMR (400 MHz) and ^{13}C NMR (100 MHz) of 4a in $CDCl_3$:

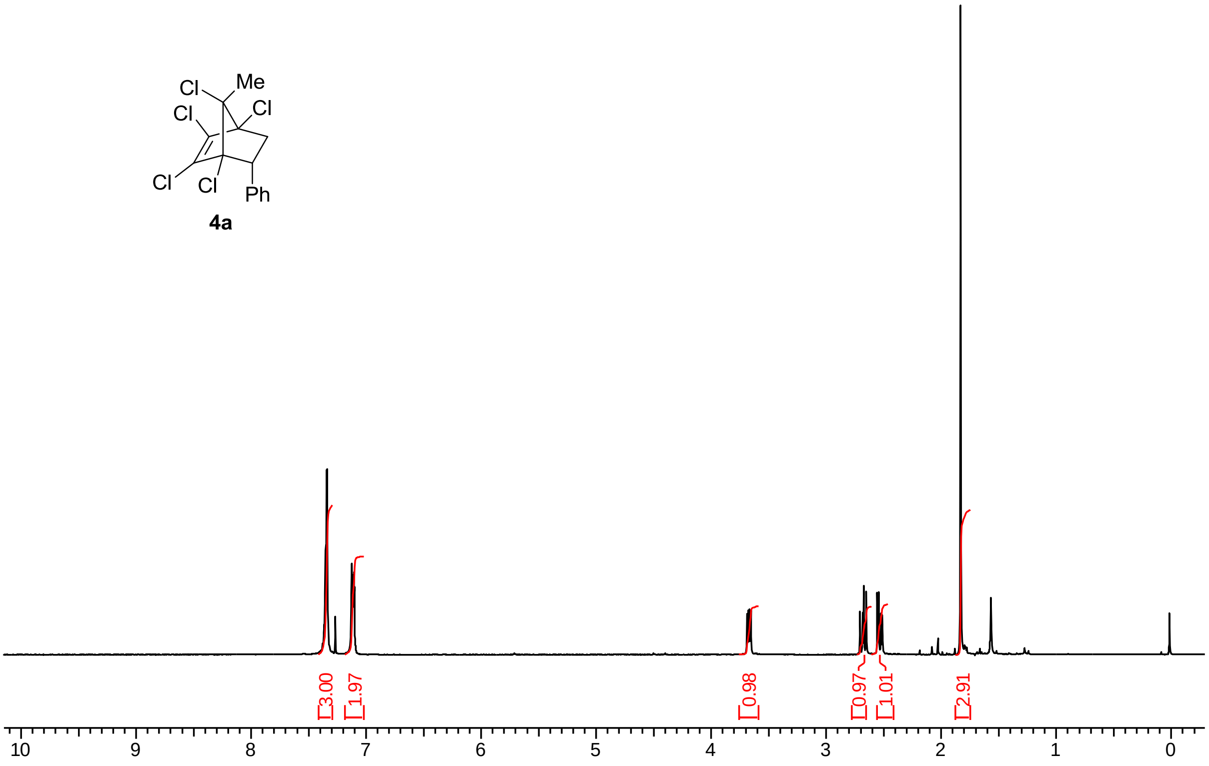

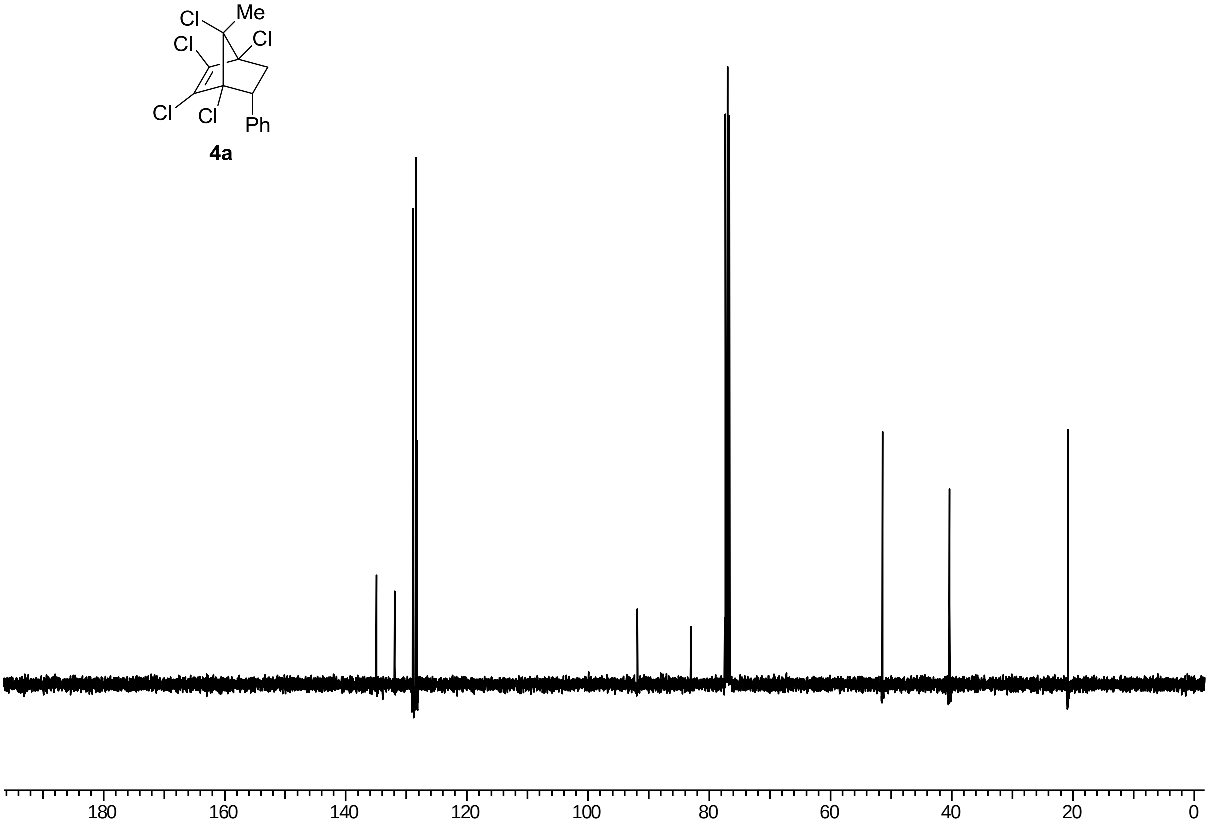

^{1}H NMR (400 MHz) and ^{13}C NMR (125 MHz) of 3b in $CDCl_3$:

3b:

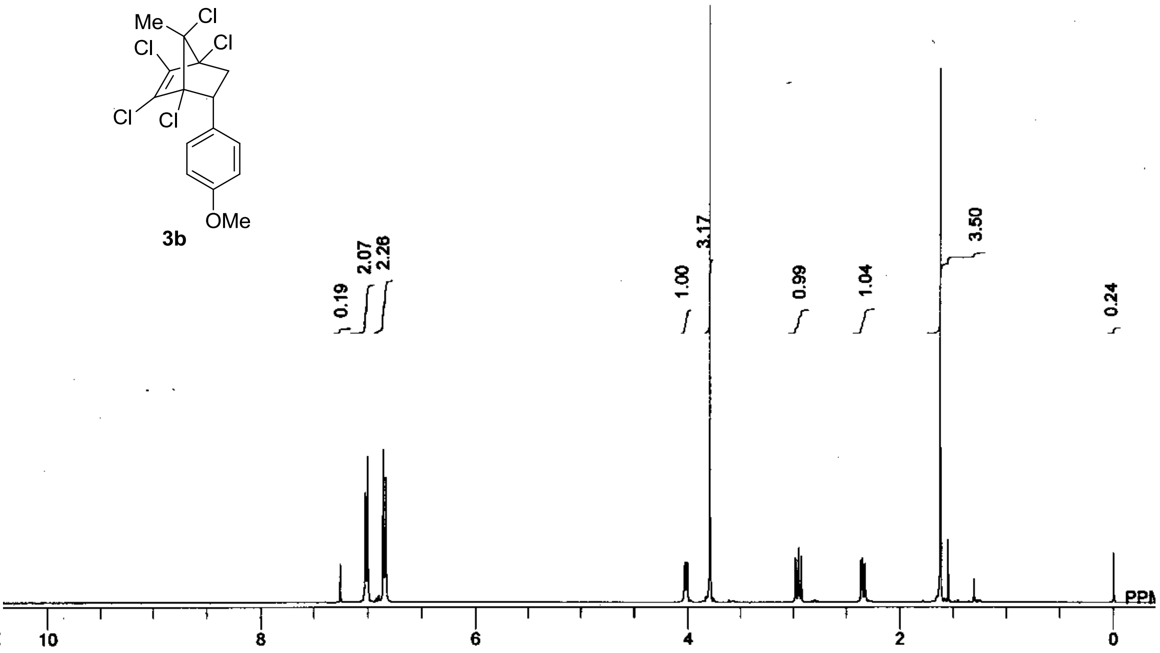

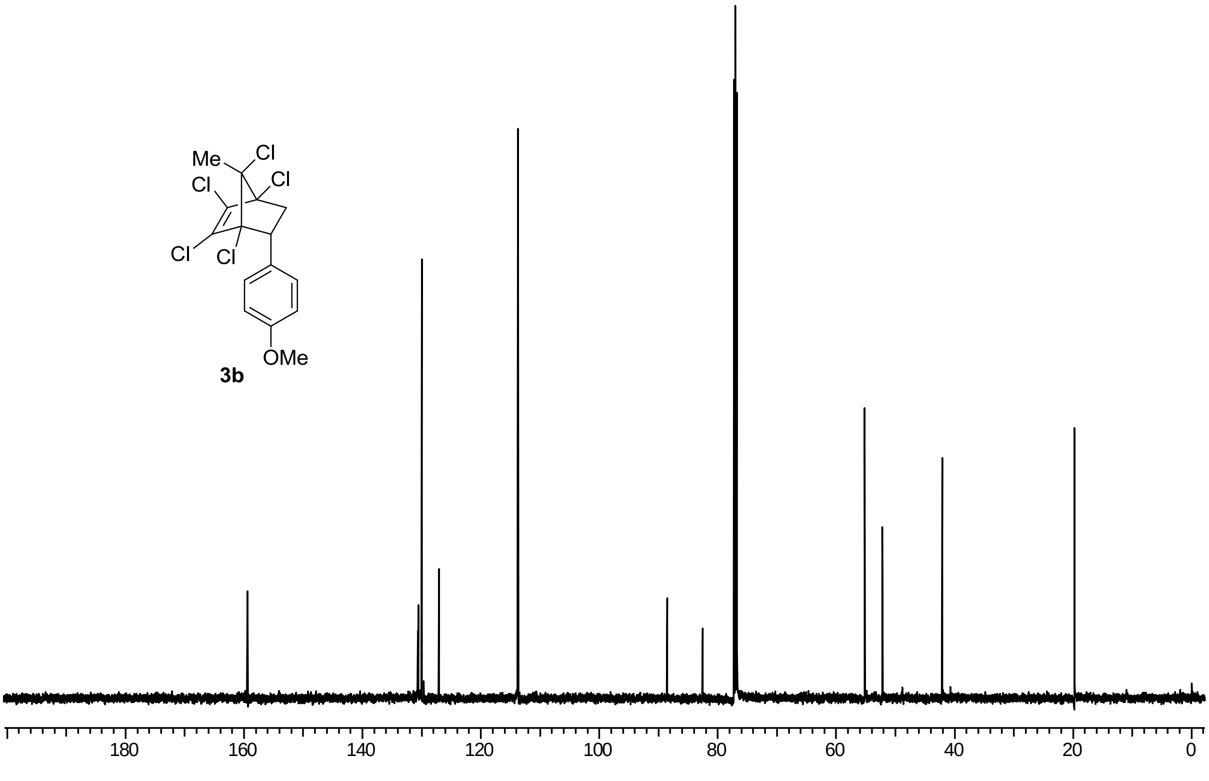

^{1}H NMR (400 MHz) and ^{13}C NMR (125 MHz) of 4b in $CDCl_3$:

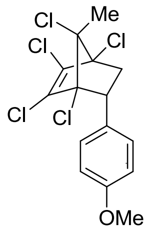

4b

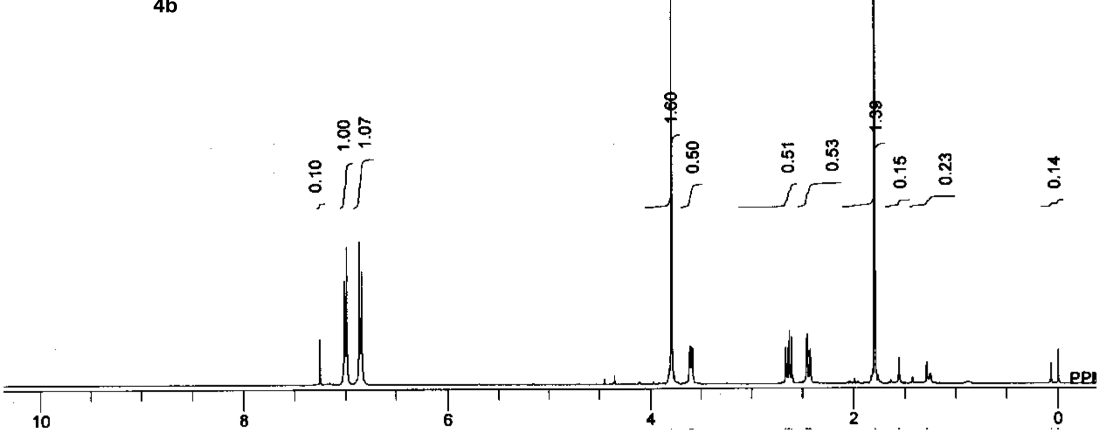

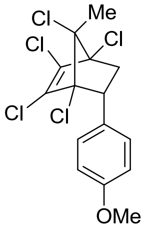

4b

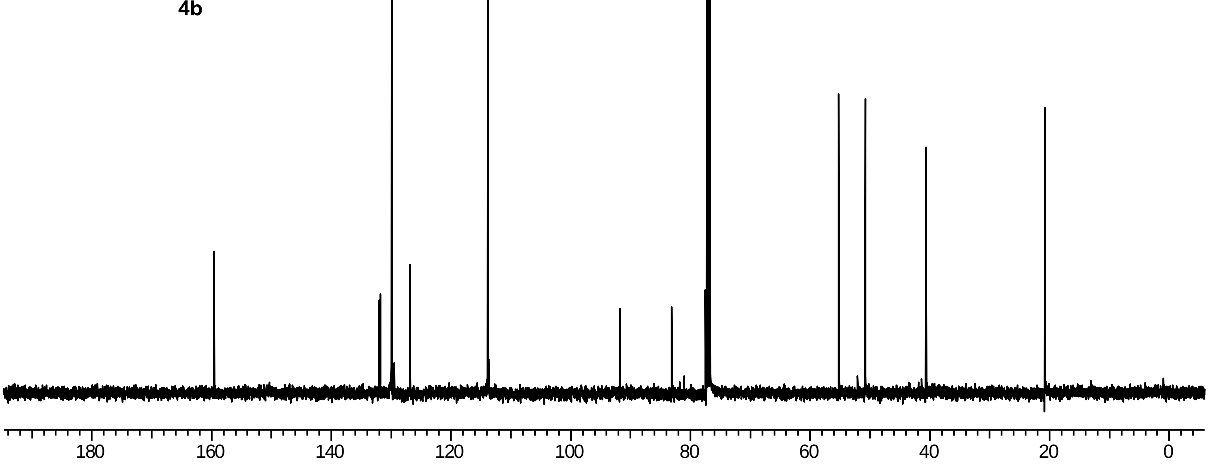

^{1}H NMR (400 MHz) and ^{13}C NMR (100 MHz) of 3c in $CDCl_3$:

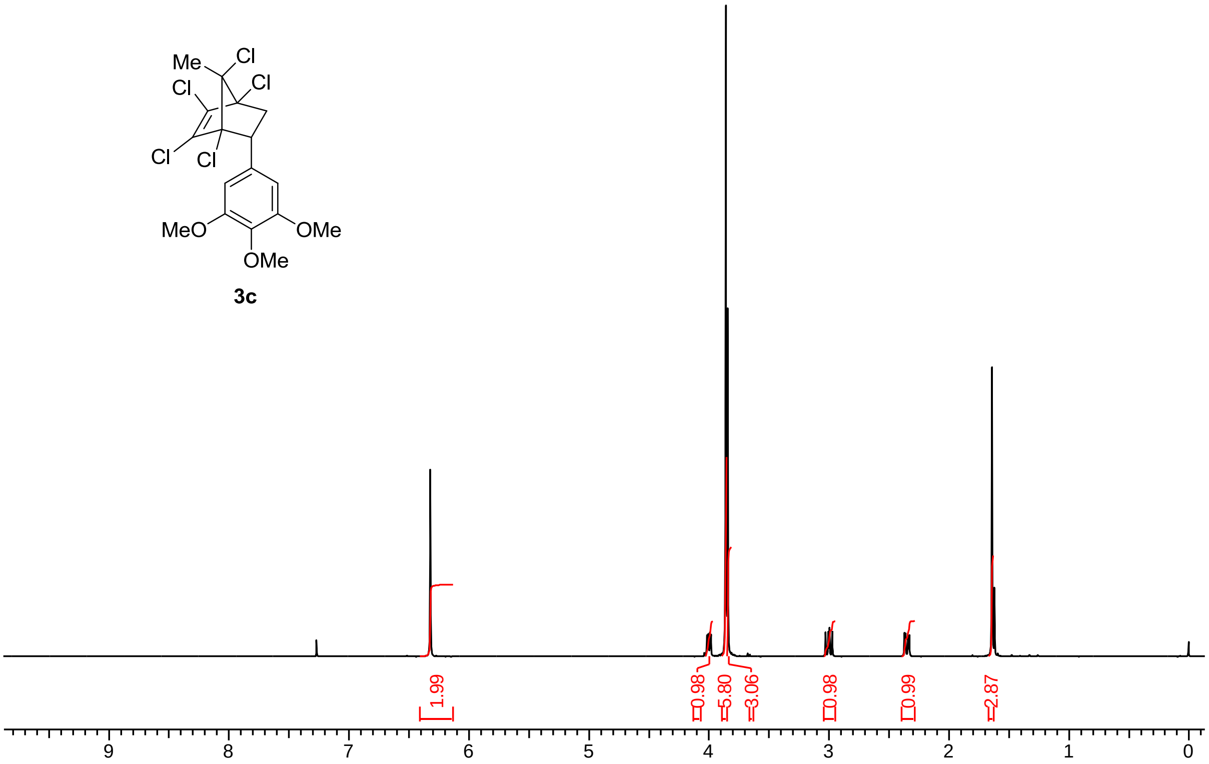

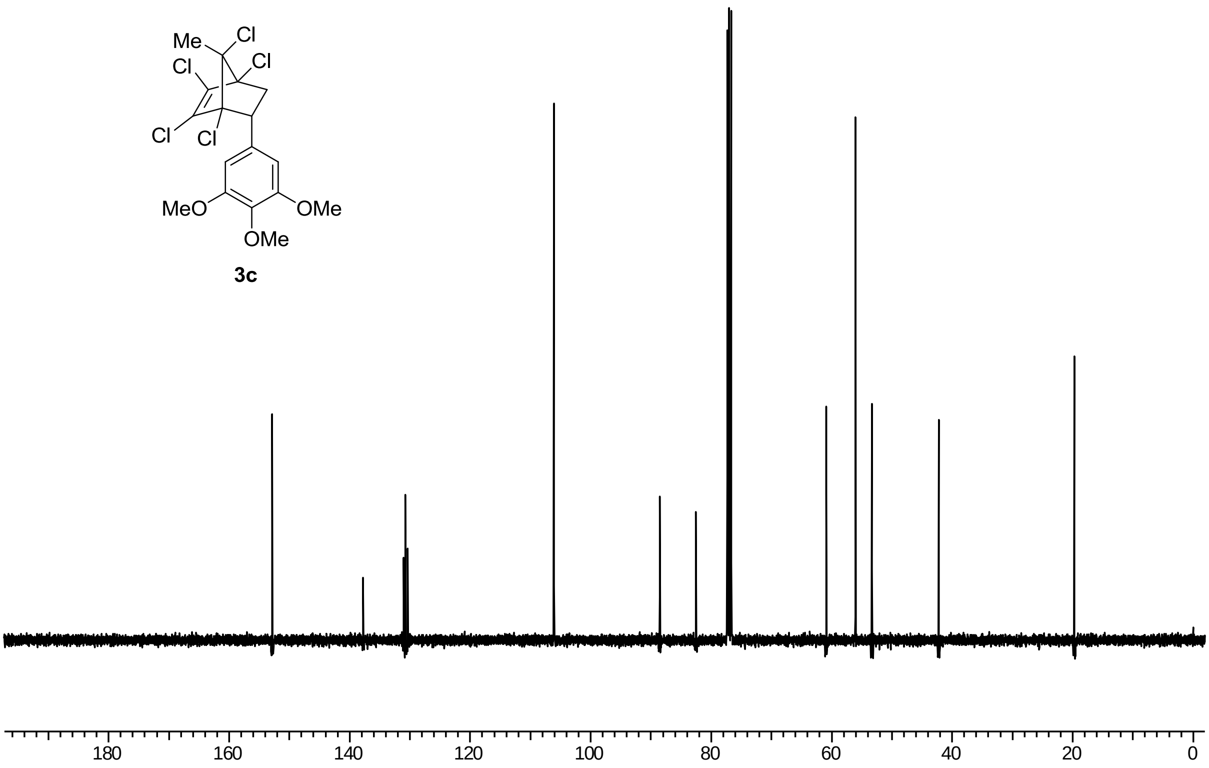

^{1}H NMR (400 MHz) and ^{13}C NMR (100 MHz) of 4c in $CDCl_3$:

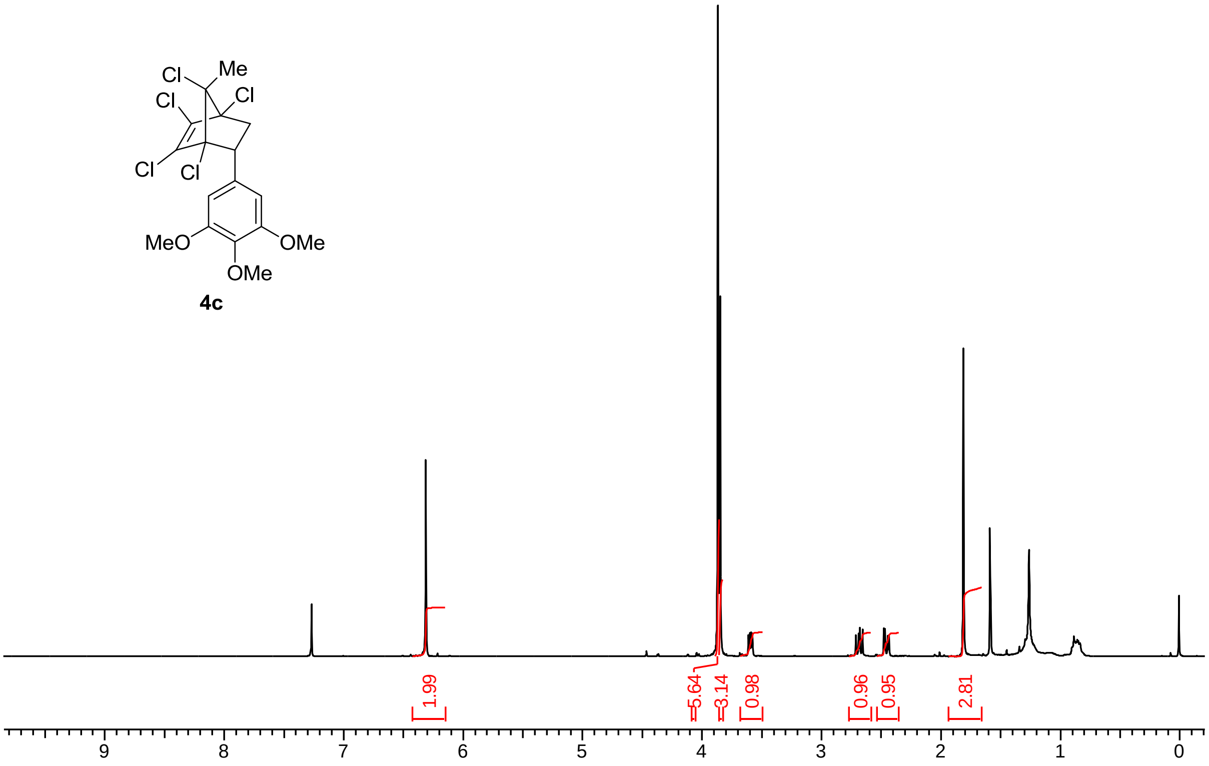

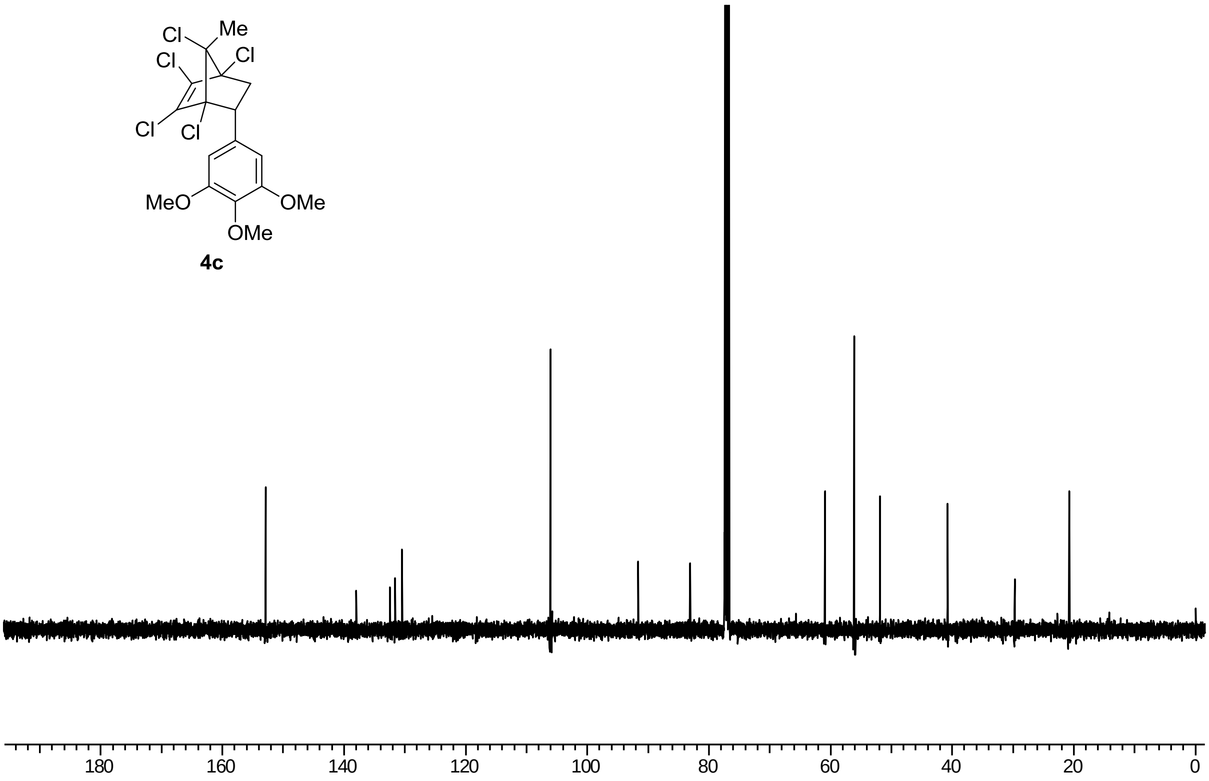

^{1}H NMR (400 MHz) and ^{13}C NMR (100 MHz) of 3d in $CDCl_3$:

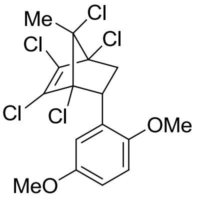

3d

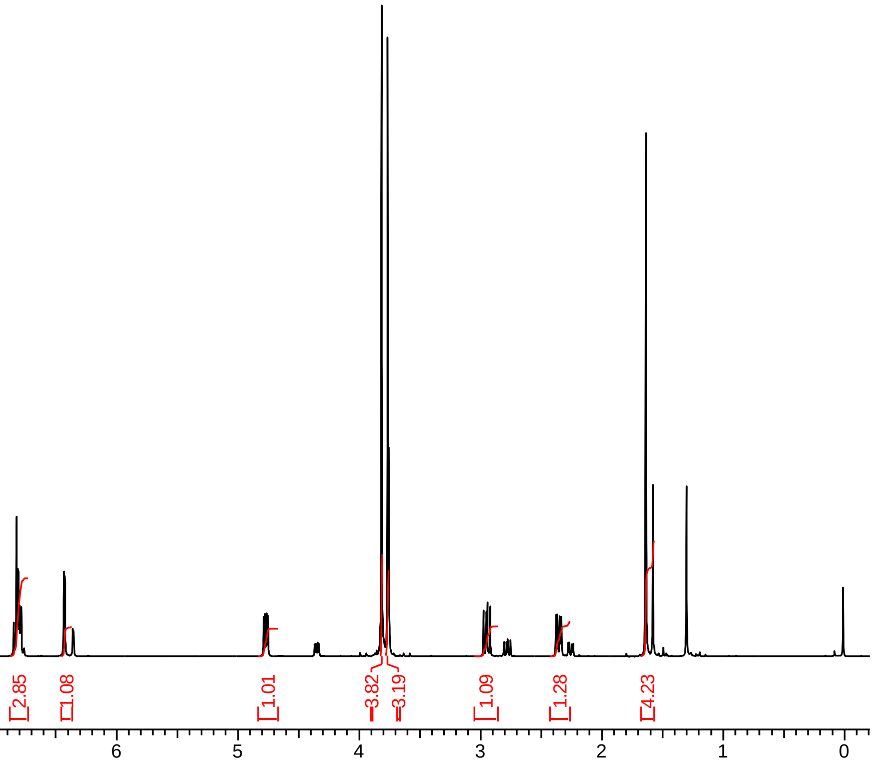

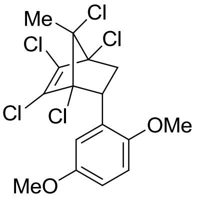

3d

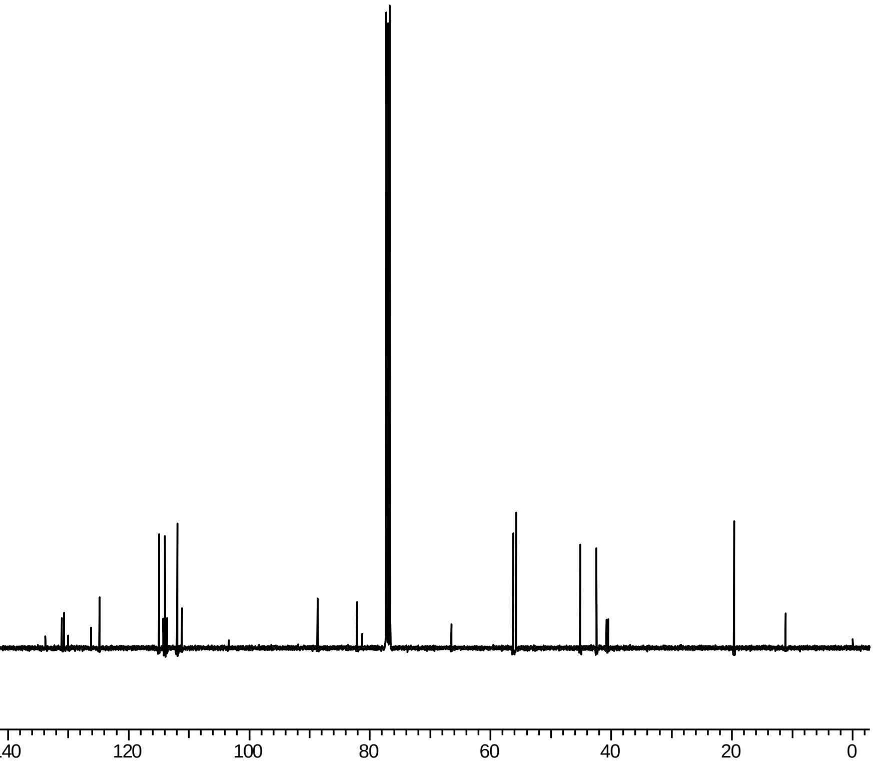

^{1}H NMR (400 MHz) and ^{13}C NMR (100 MHz) of 4d in $CDCl_3$:

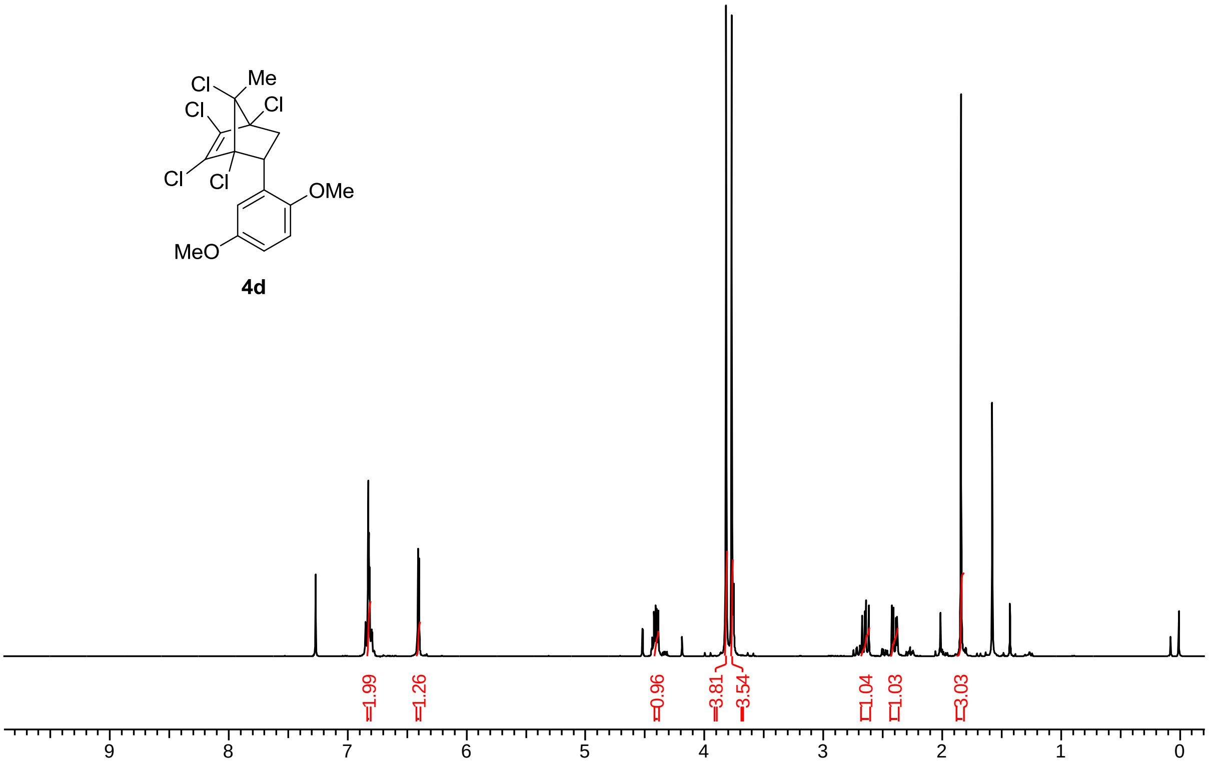

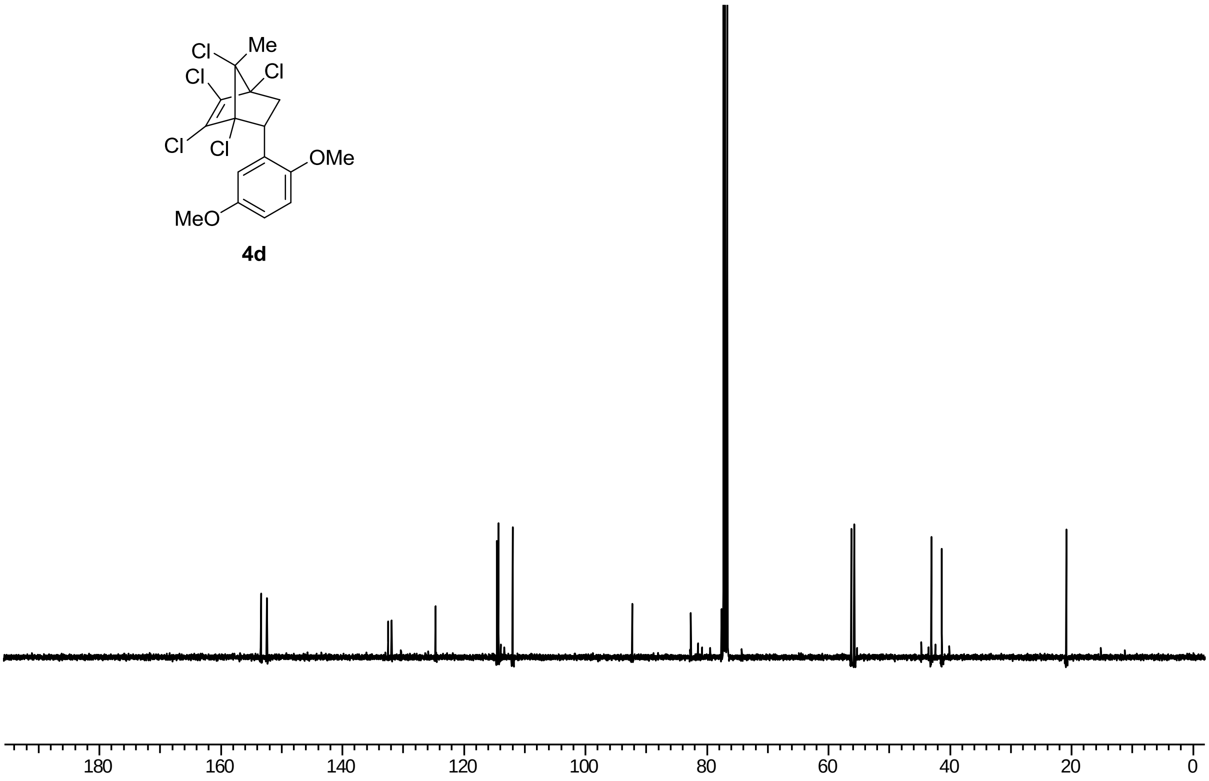

^1H NMR (400 MHz) and ^{13}C NMR (125 MHz) of 3e in $CDCl_3$:

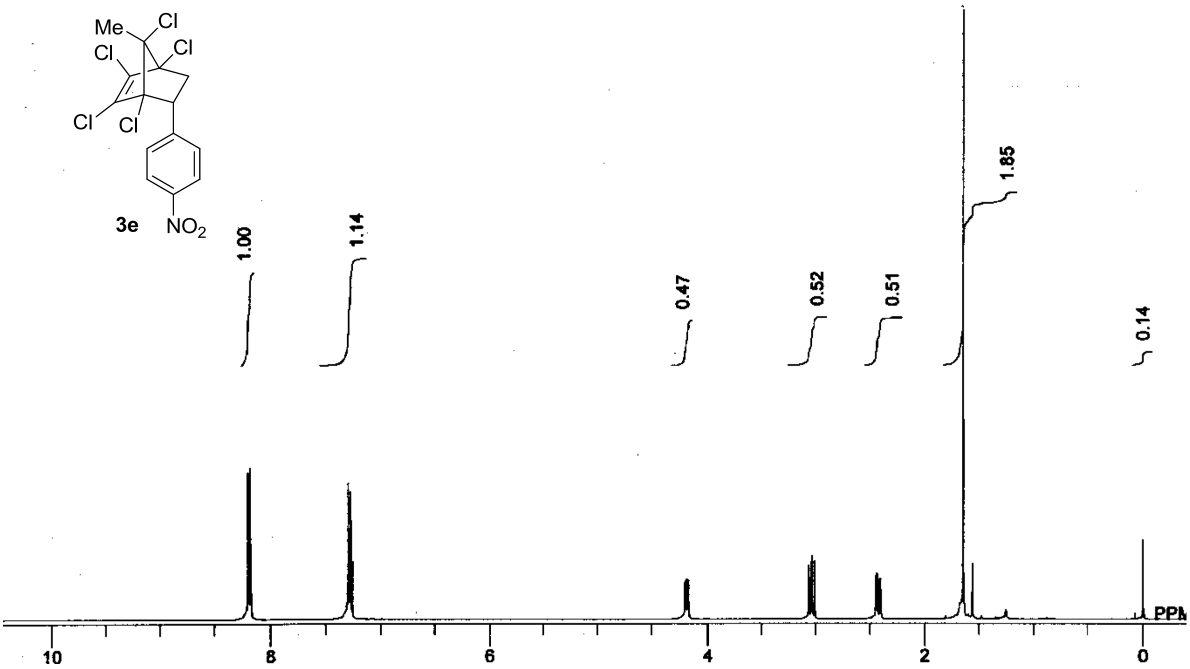

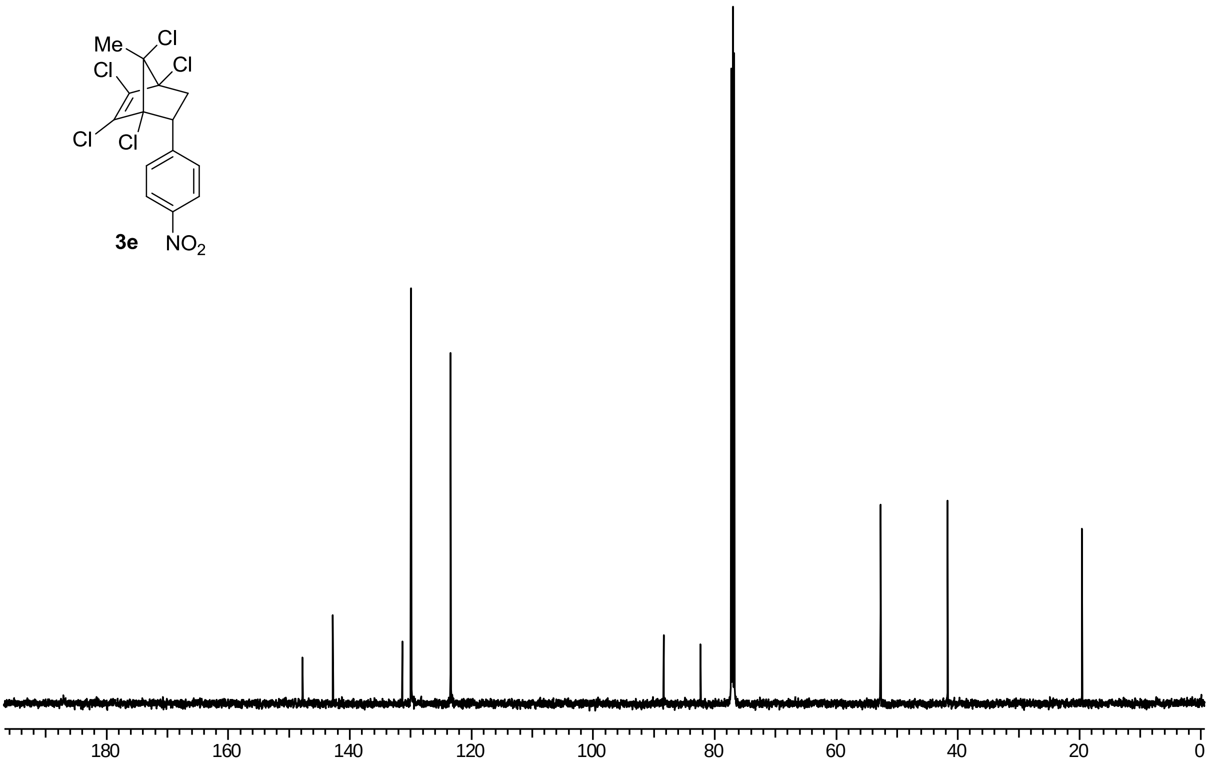

^{1}H NMR (400 MHz) and ^{13}C NMR (125 MHz) of 3f in $CDCl_3$:

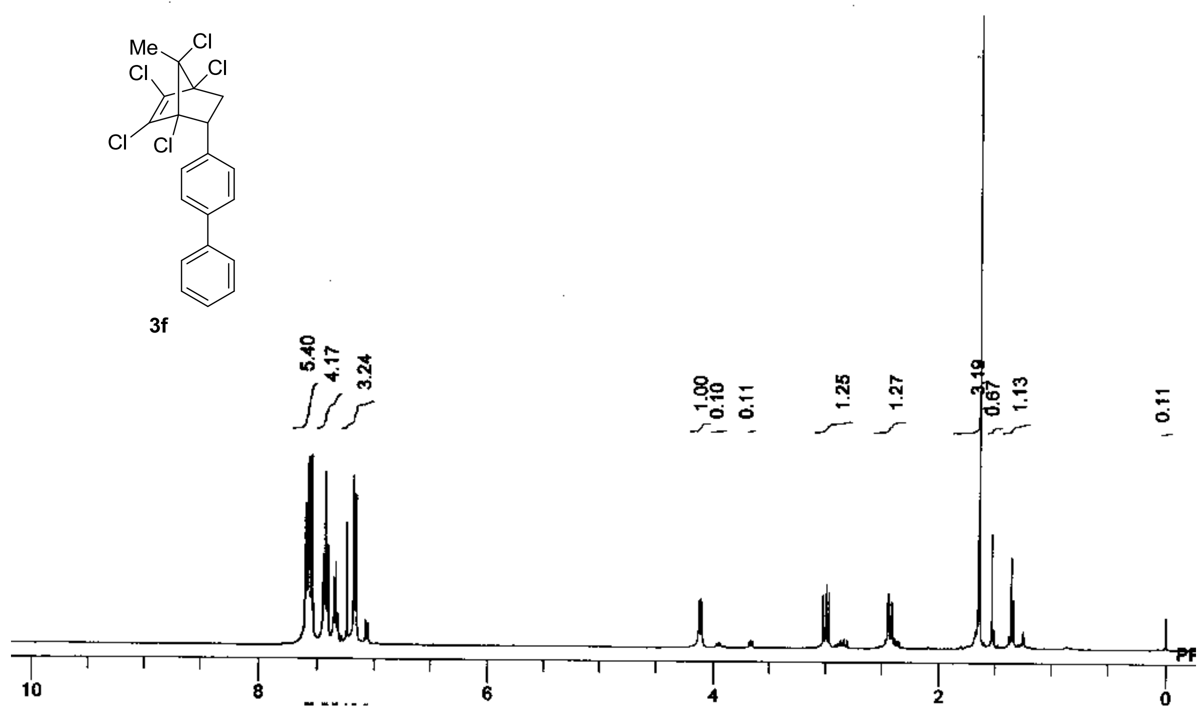

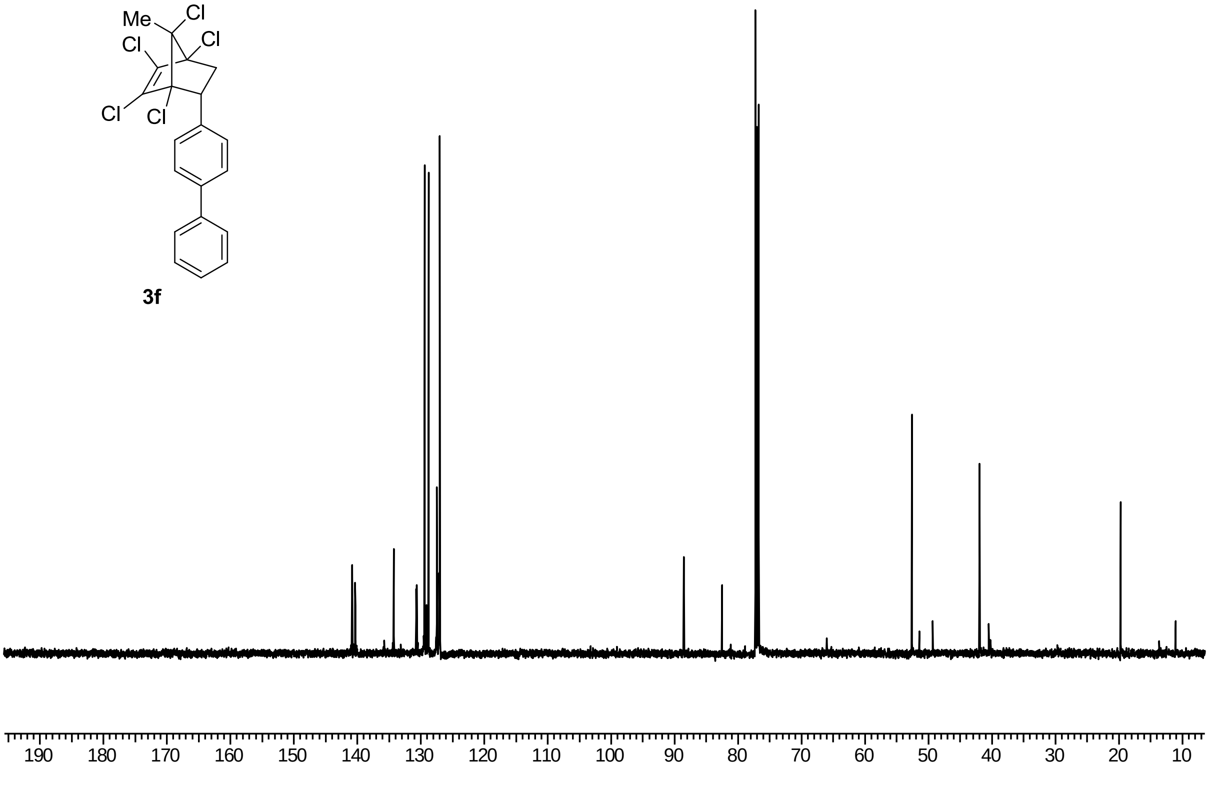

^{1}H NMR (400 MHz) and ^{13}C NMR (125 MHz) of 4f in $CDCl_3$:

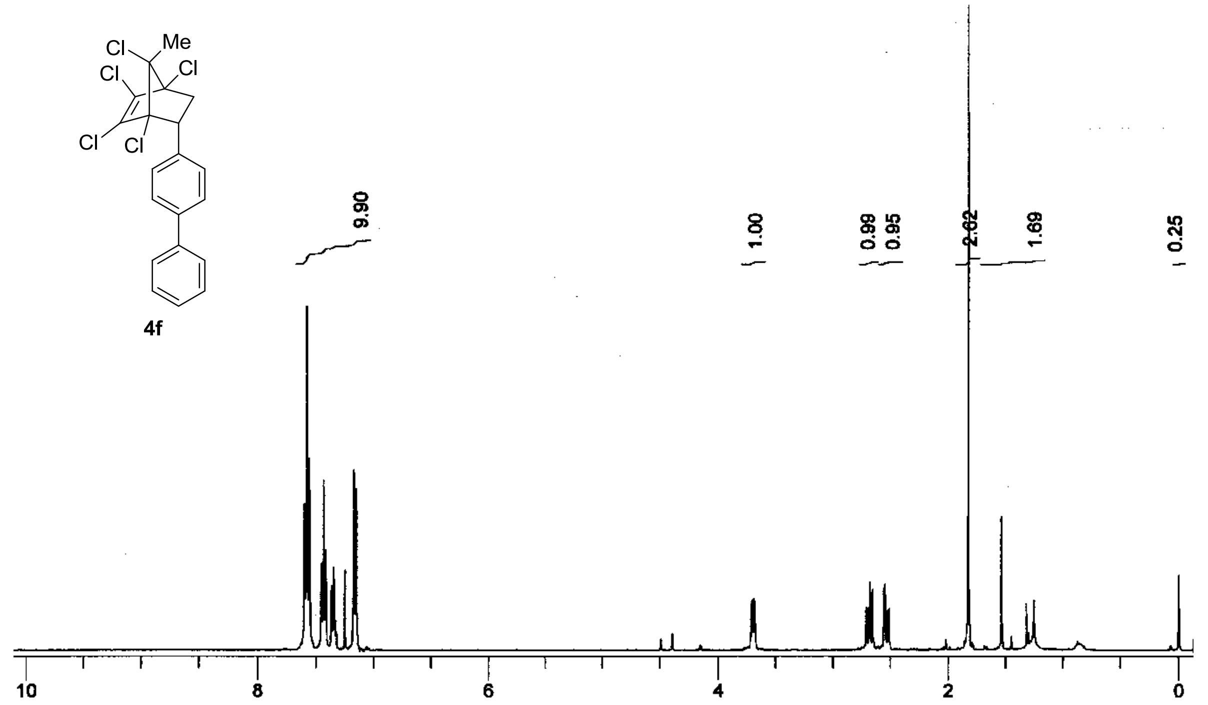

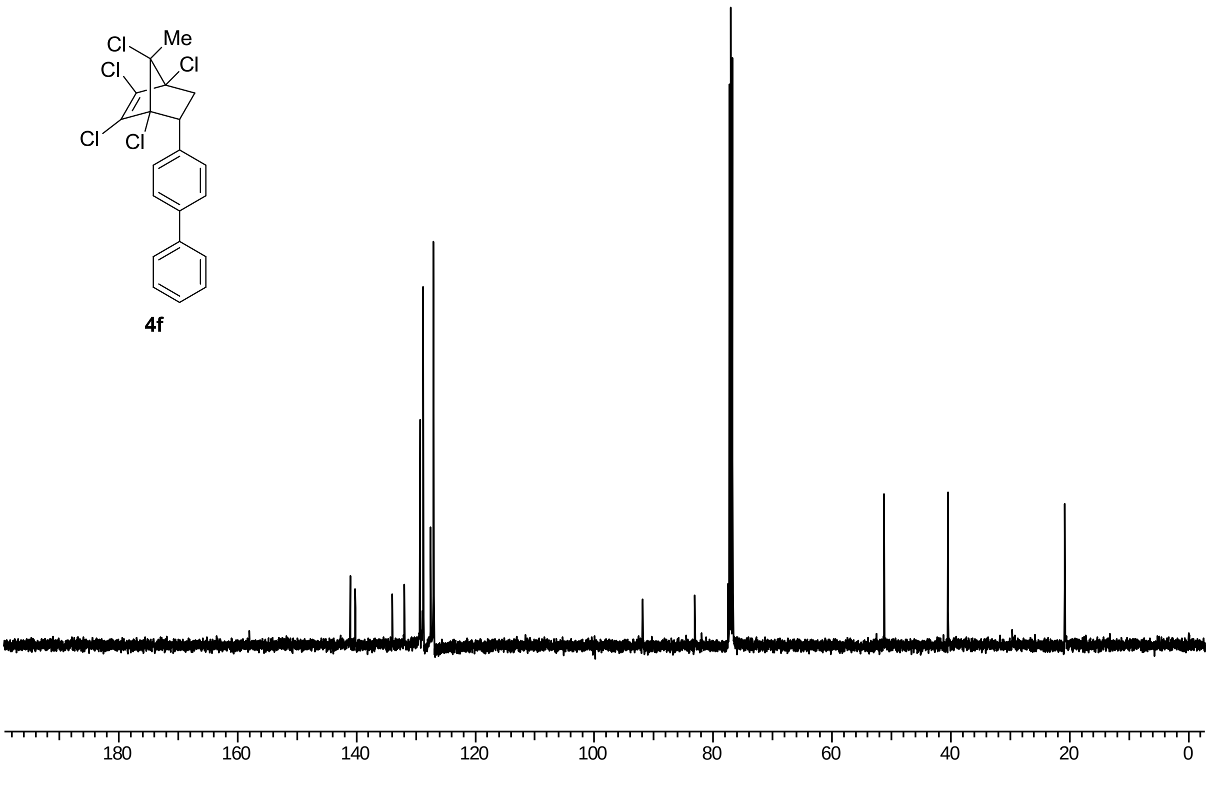

^{1}H NMR (500 MHz) and ^{13}C NMR (125 MHz) of 3g in $CDCl_3$:

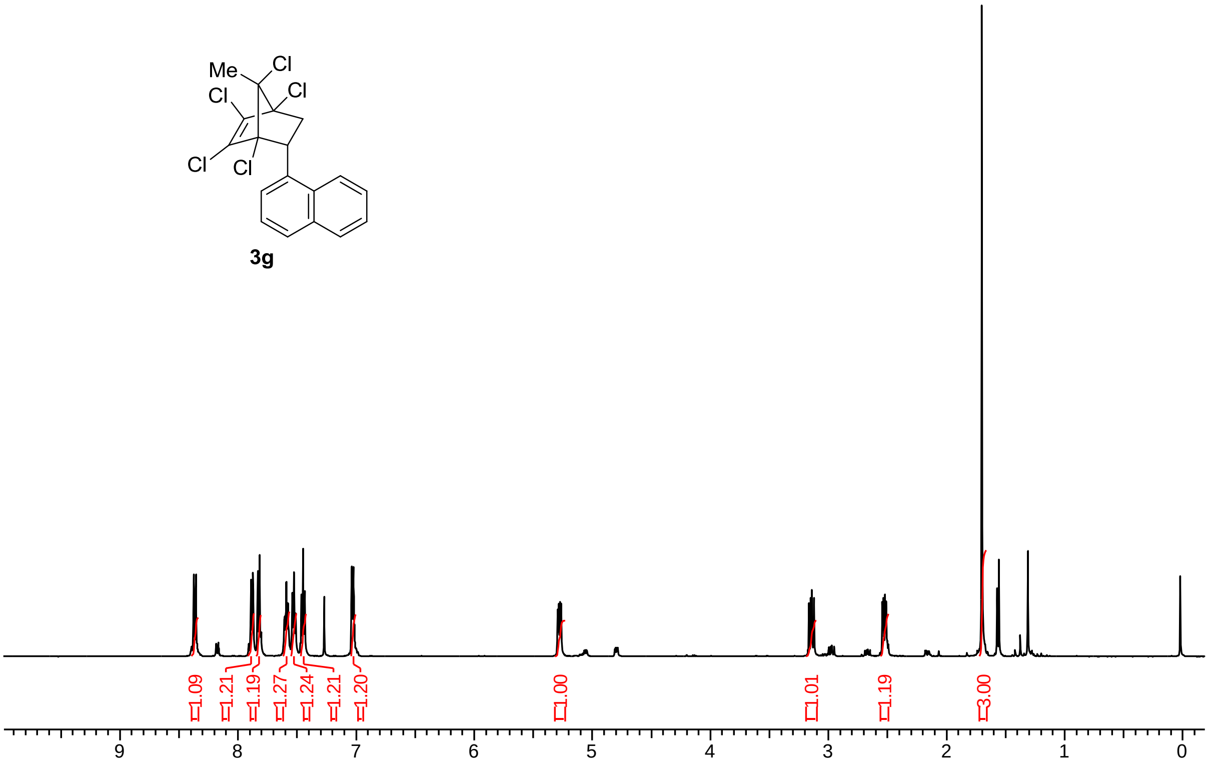

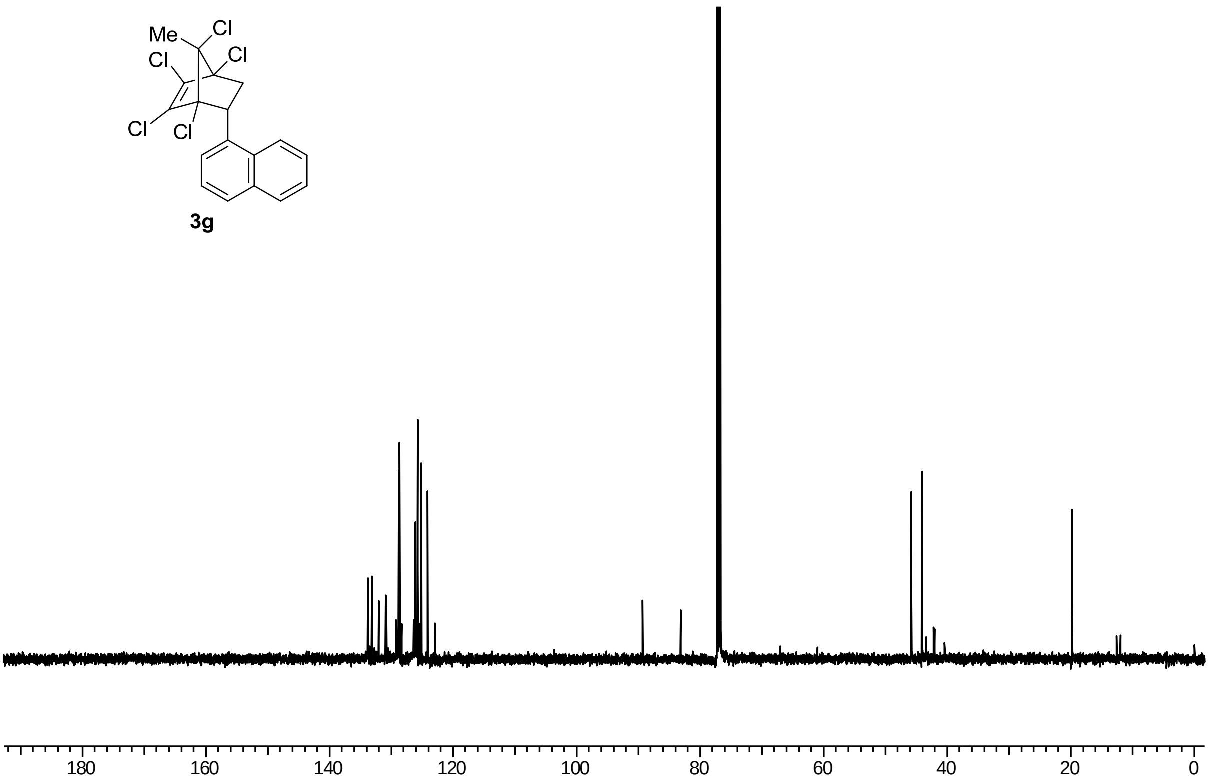

^{1}H NMR (500 MHz) and ^{13}C NMR (125 MHz) of 4g in $CDCl_3$:

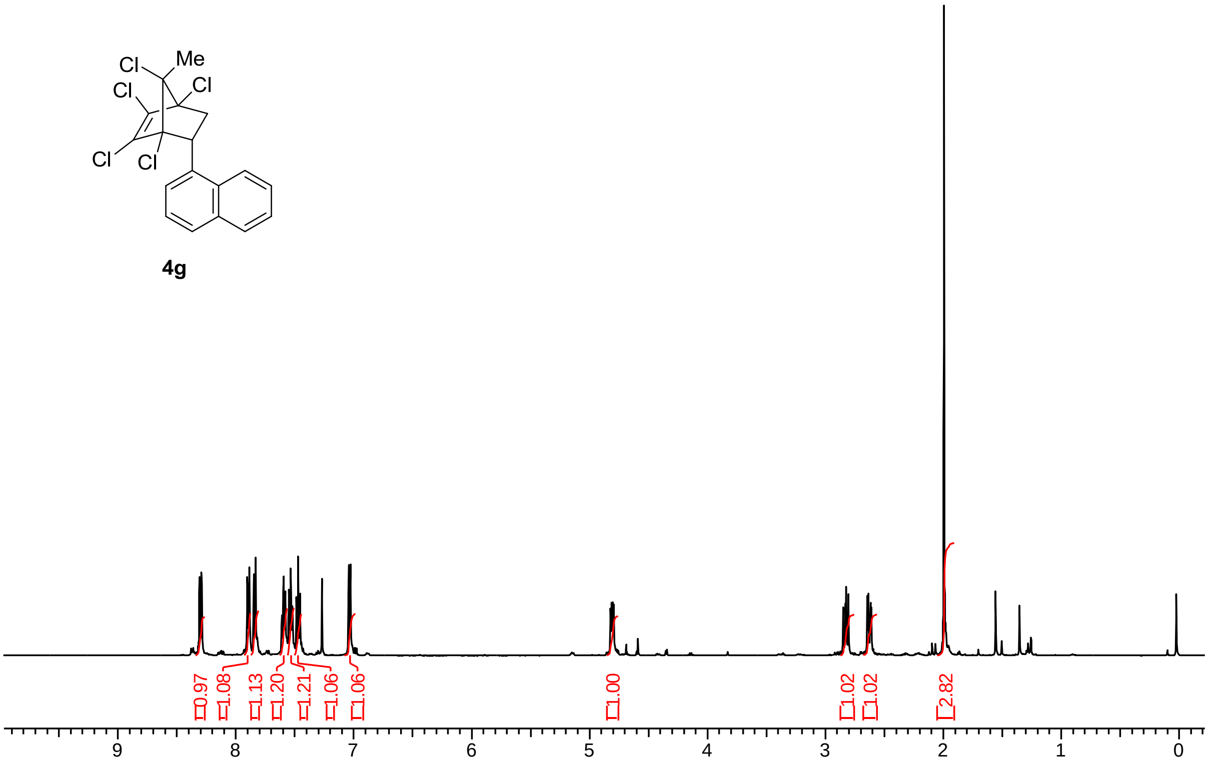

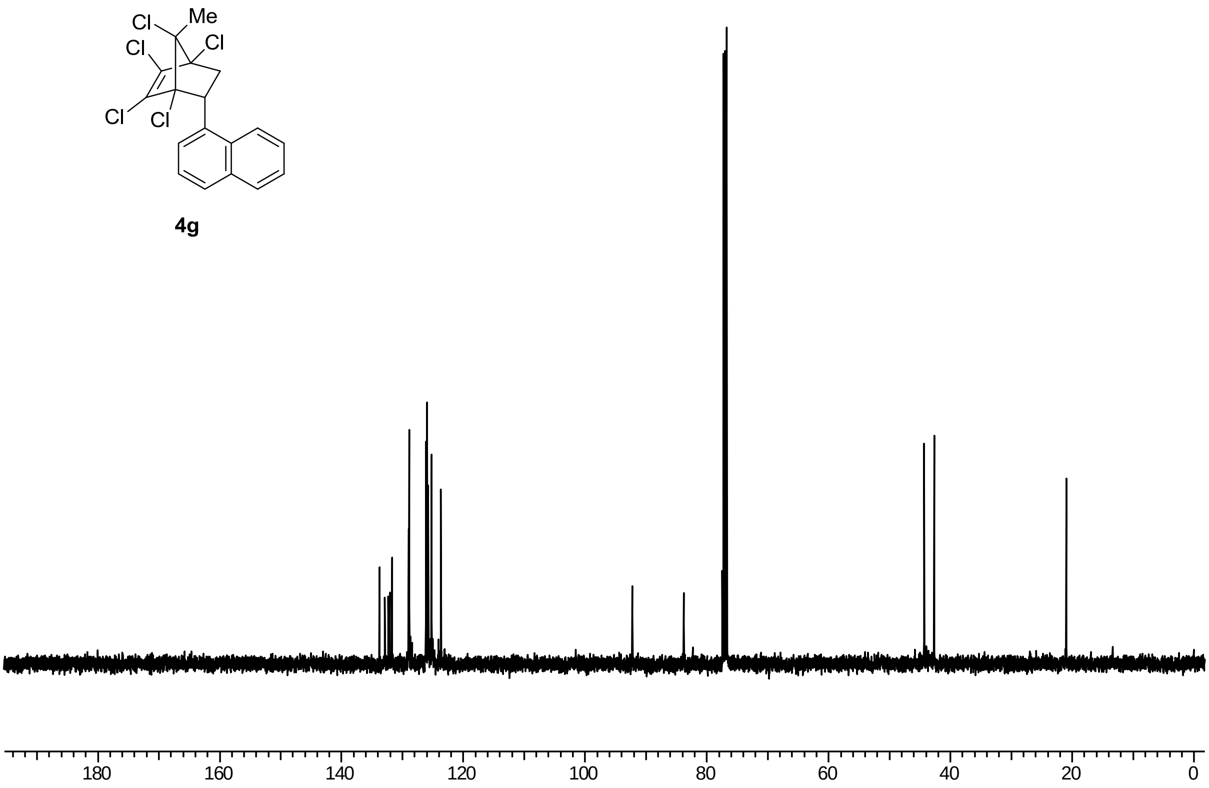

^{1}H NMR (400 MHz) and ^{13}C NMR (100 MHz) of 3h in $CDCl_3$:

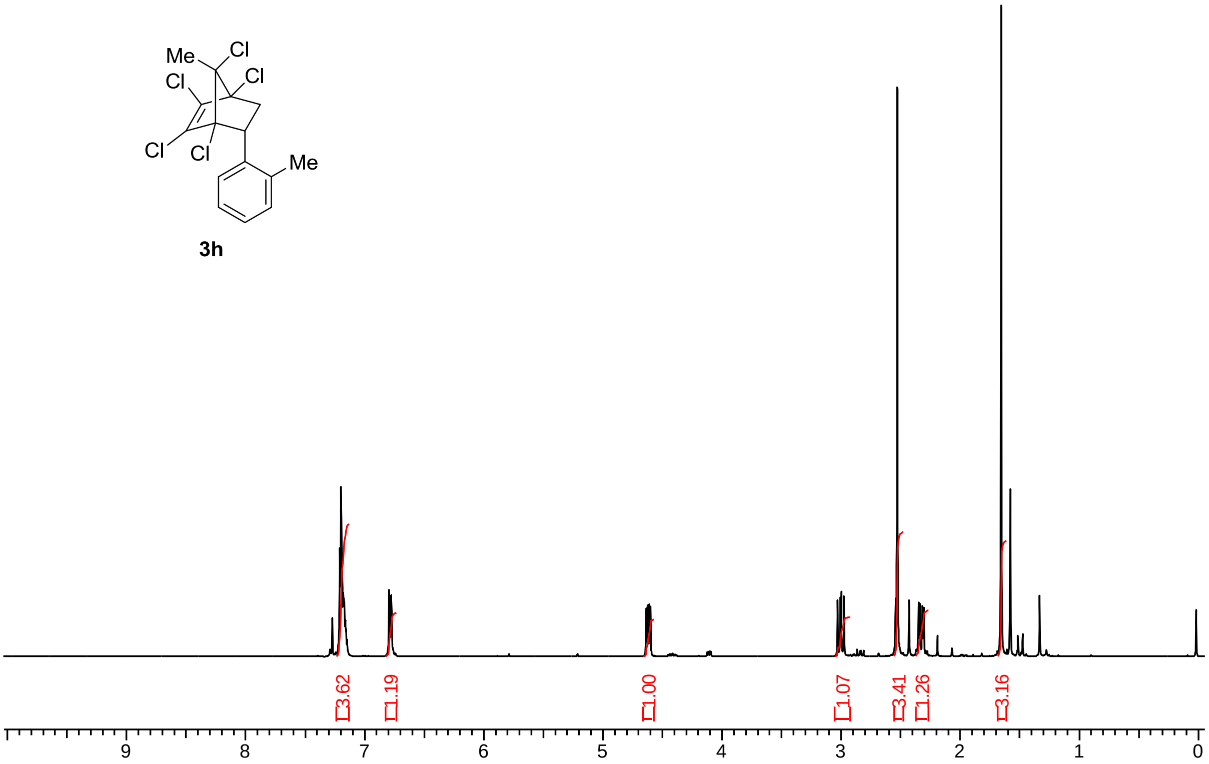

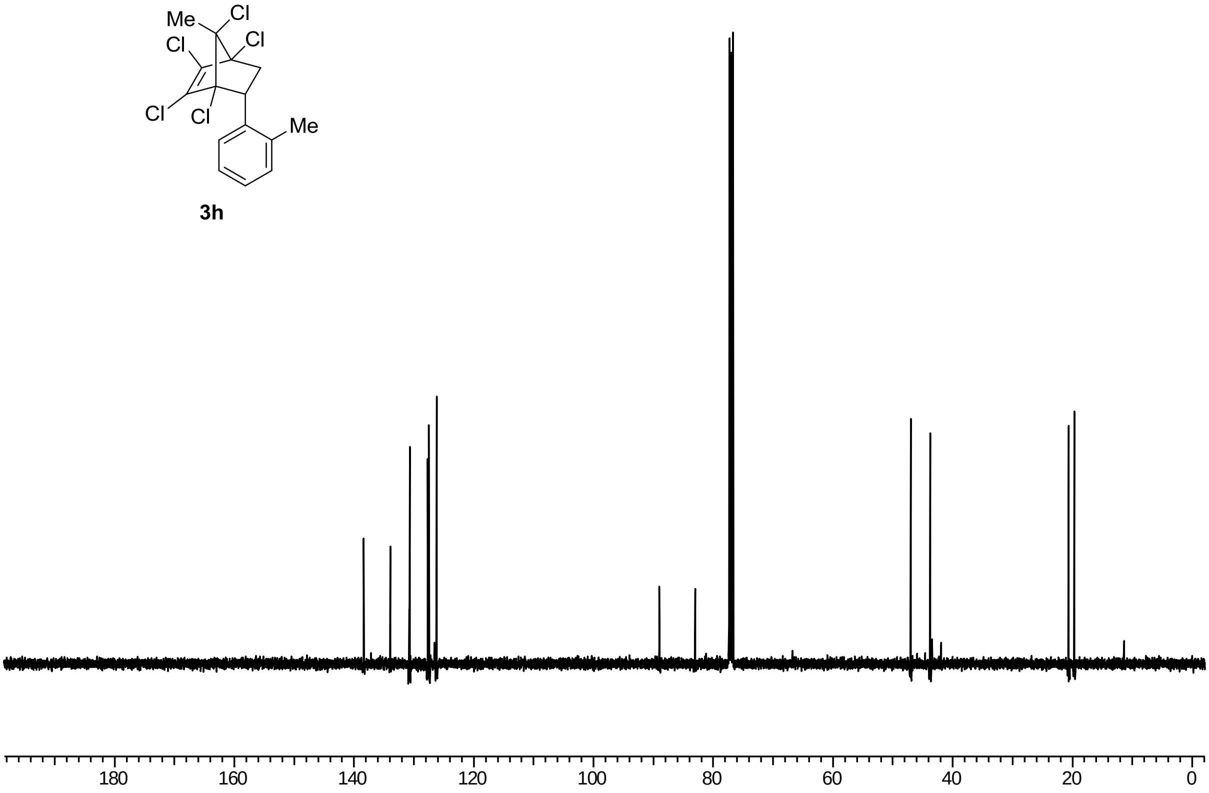

^{1}H NMR (400 MHz) and ^{13}C NMR (100 MHz) of 4h in $CDCl_3$:

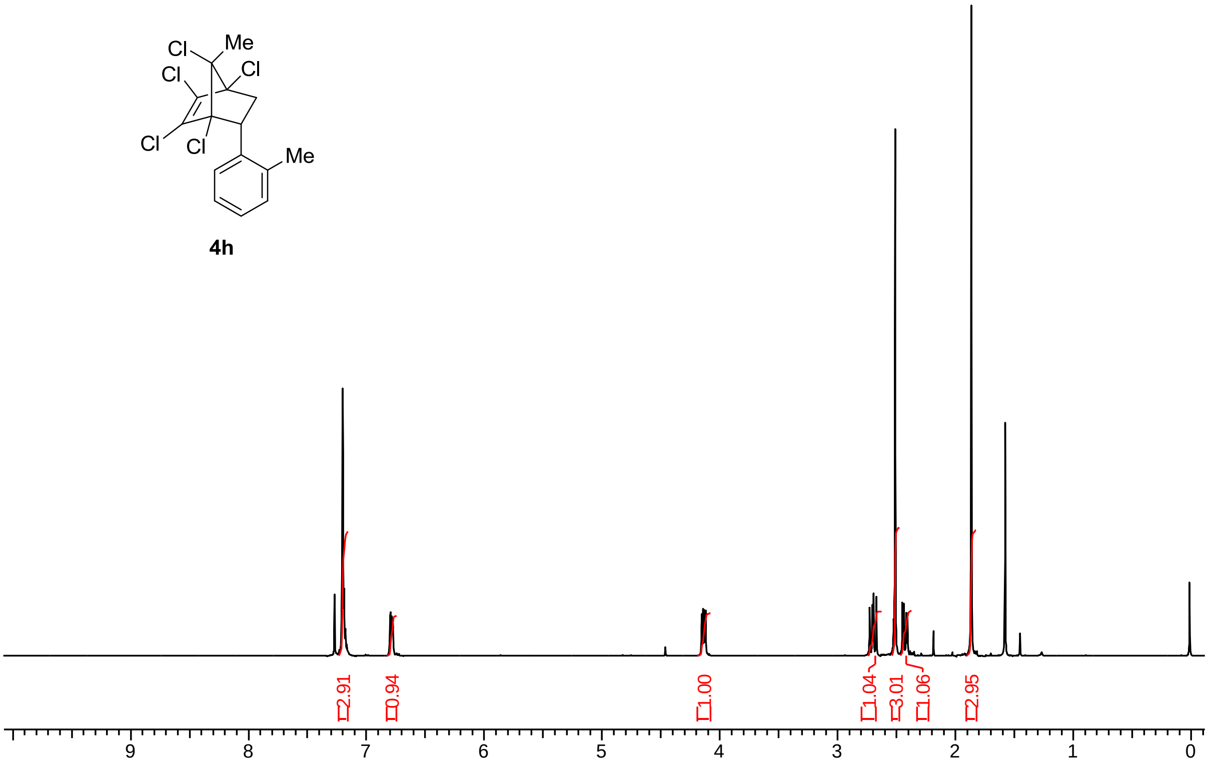

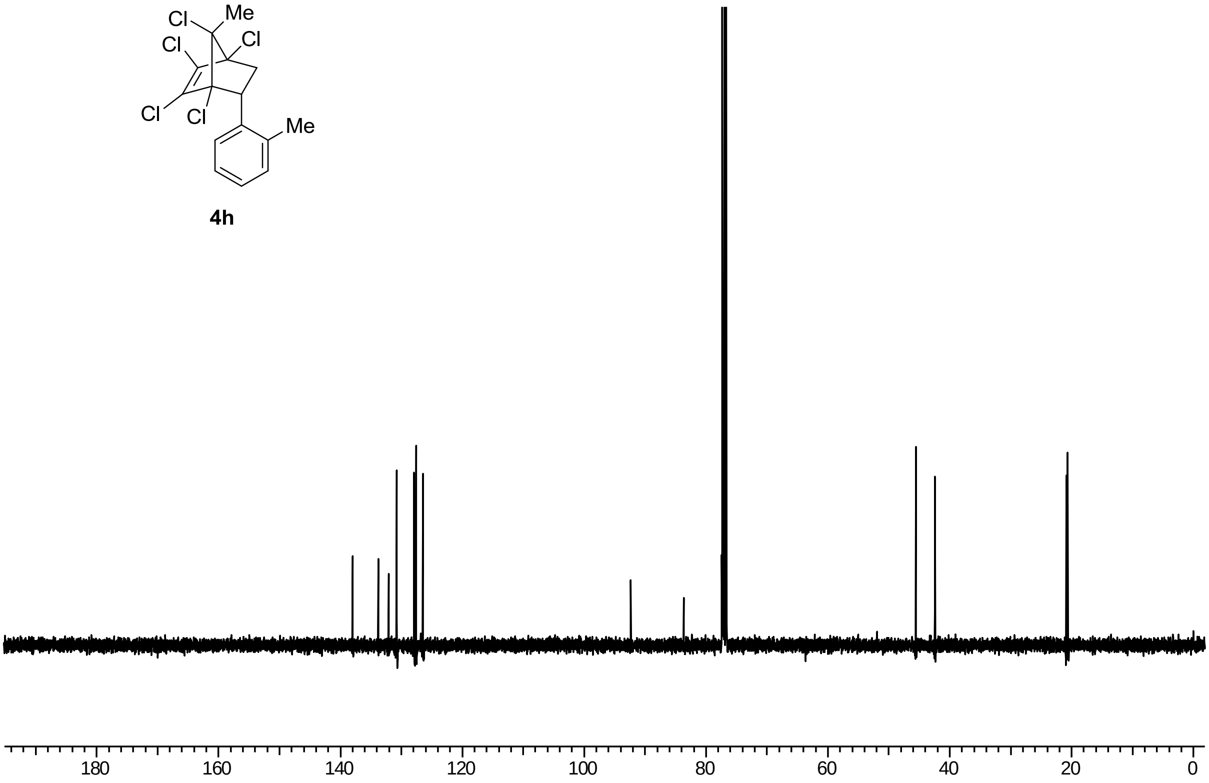

^{1}H NMR (400 MHz) and ^{13}C NMR (100 MHz) of 3i in $CDCl_3$:

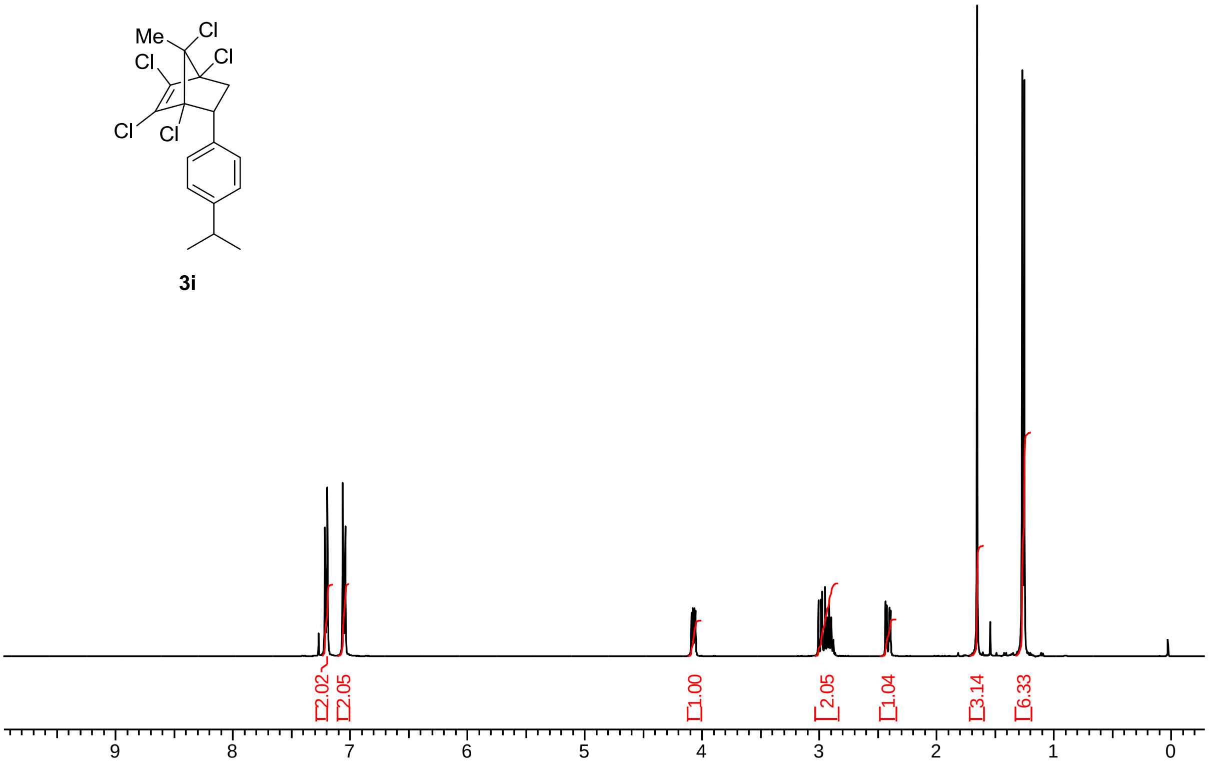

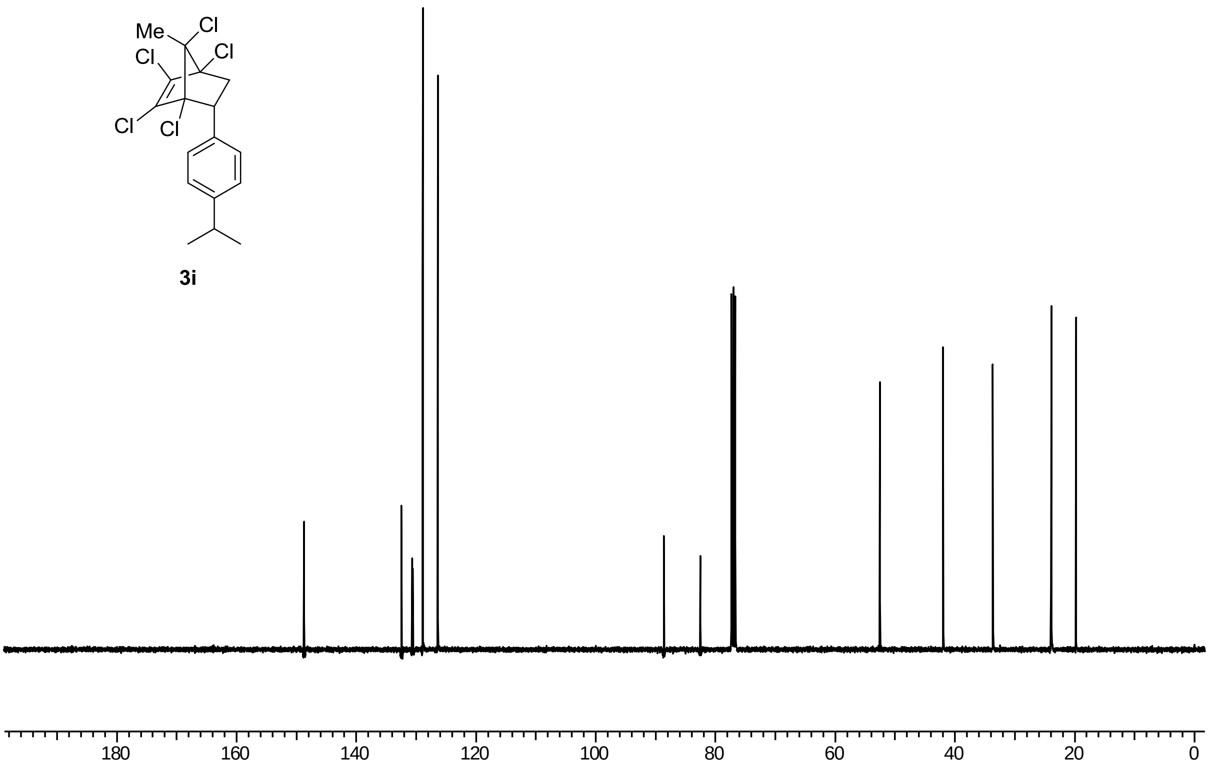

^{1}H NMR (400 MHz) and ^{13}C NMR (100 MHz) of 4i in $CDCl_3$:

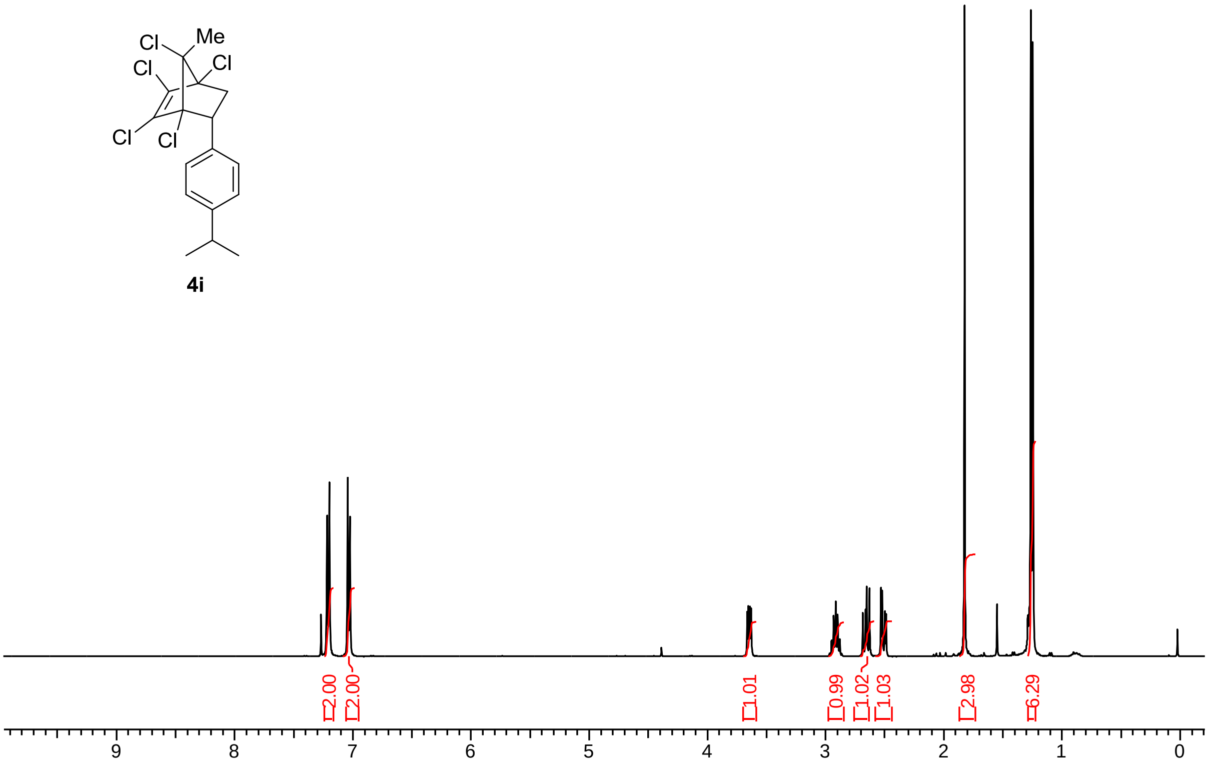

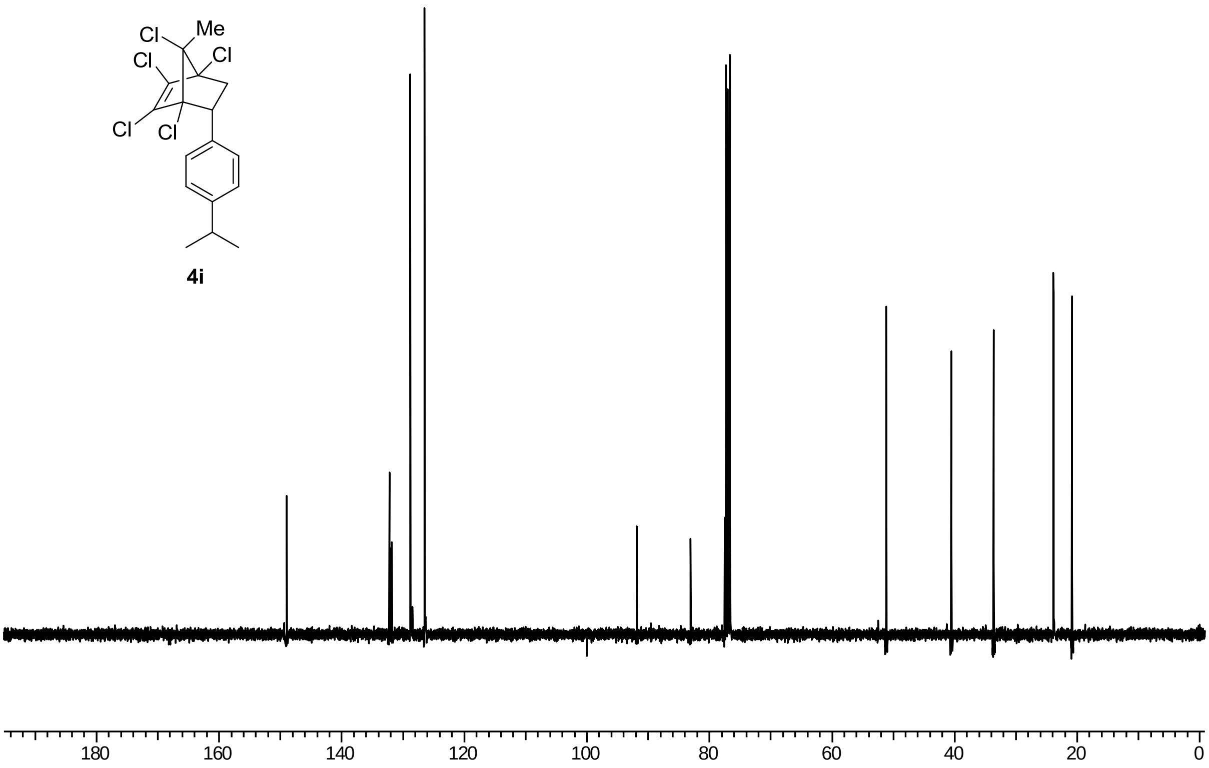

^{1}H NMR (400 MHz) and ^{13}C NMR (100 MHz) of 3j in $CDCl_3$:

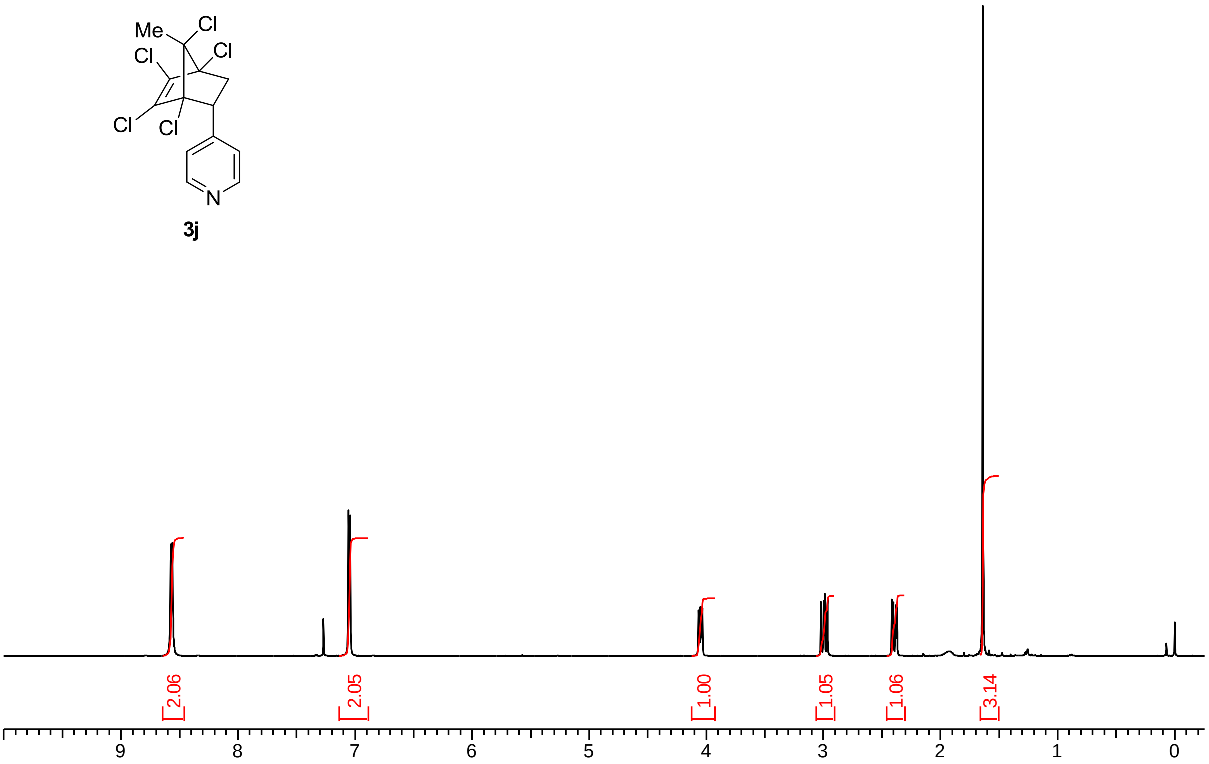

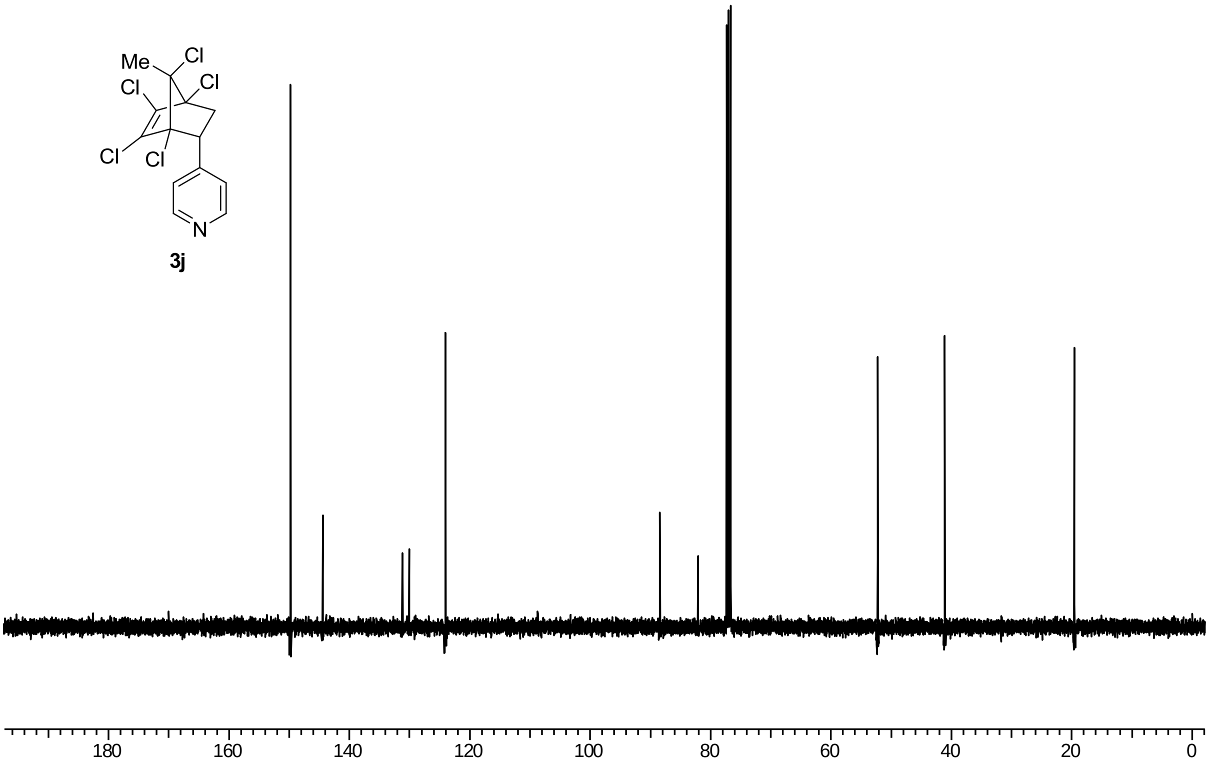

^{1}H NMR (400 MHz) and ^{13}C NMR (100 MHz) of 3k in $CDCl_3$:

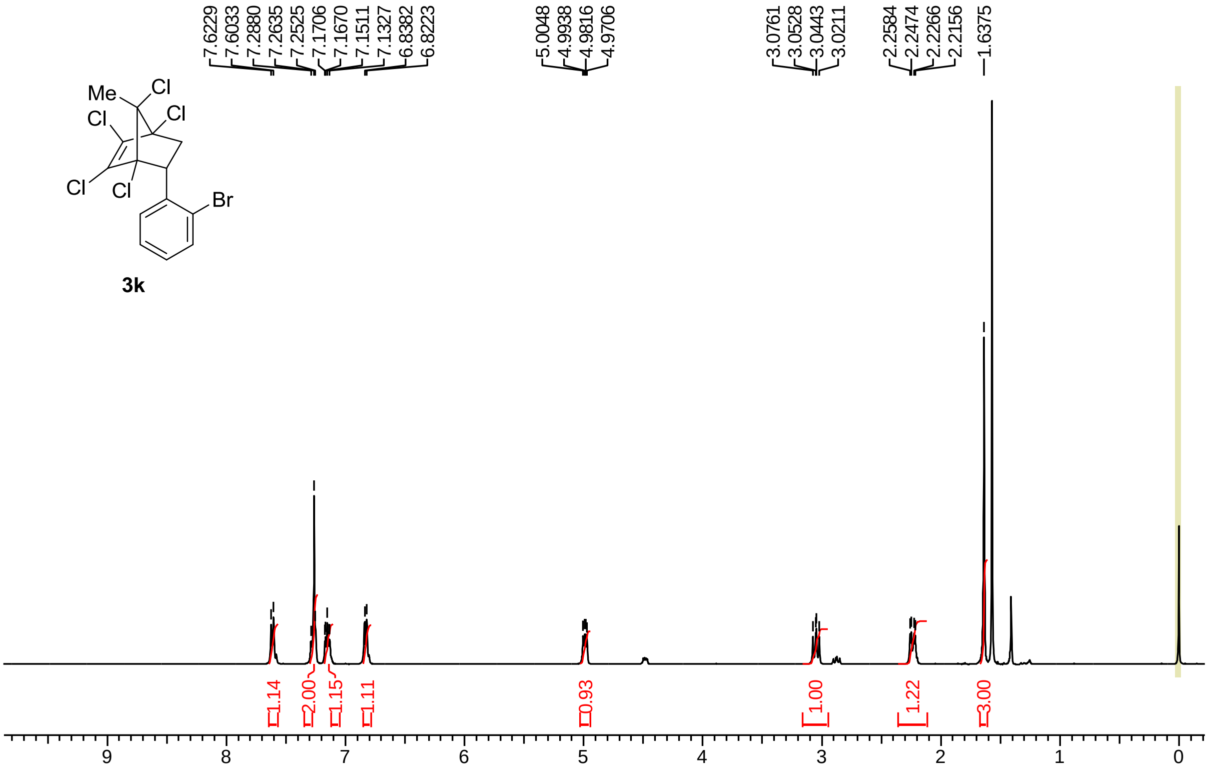

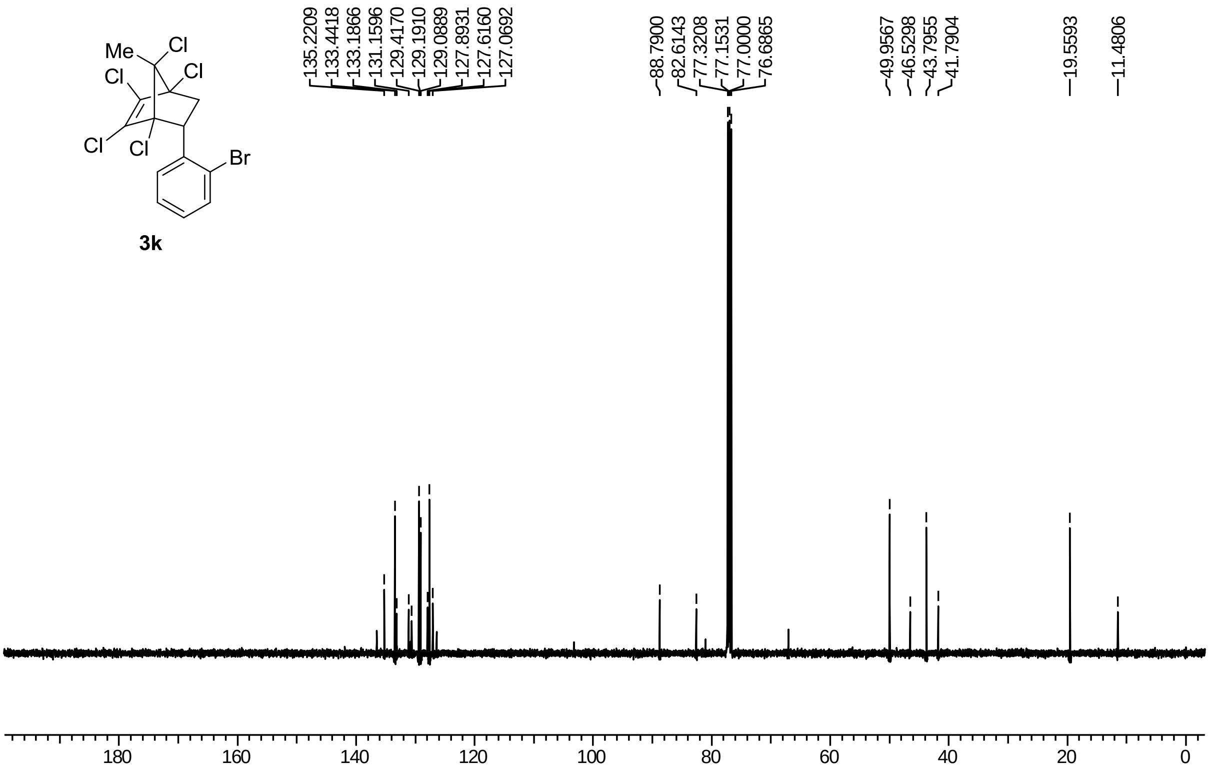

^{1}H NMR (400 MHz) and ^{13}C NMR (100 MHz) of 4k in $CDCl_3$:

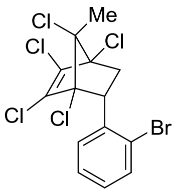

4k

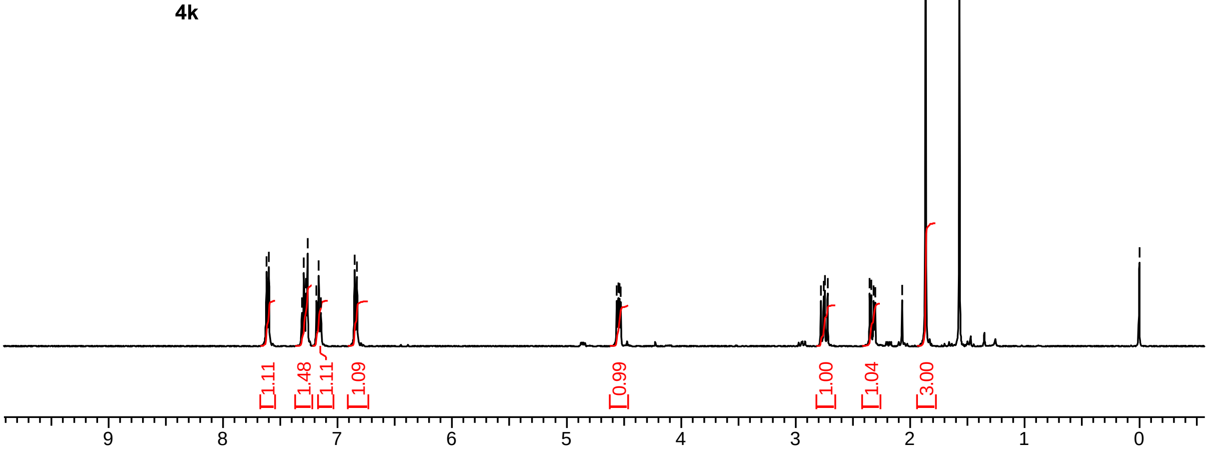

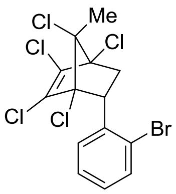

4k

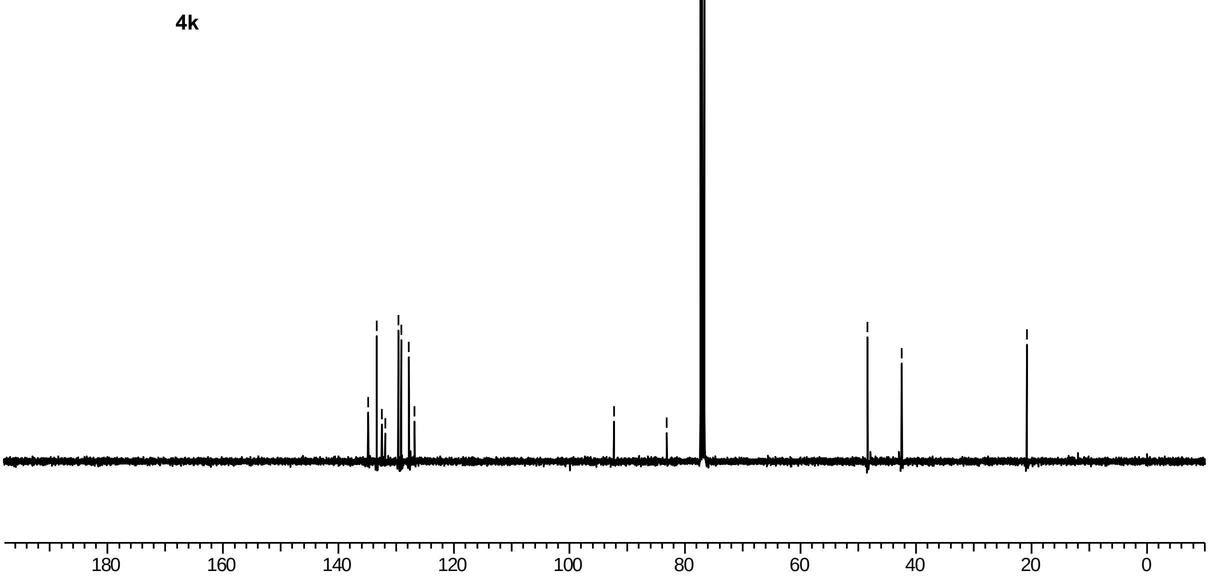

^{1}H NMR (400 MHz) and ^{13}C NMR (100 MHz) of 3l in $CDCl_3$:

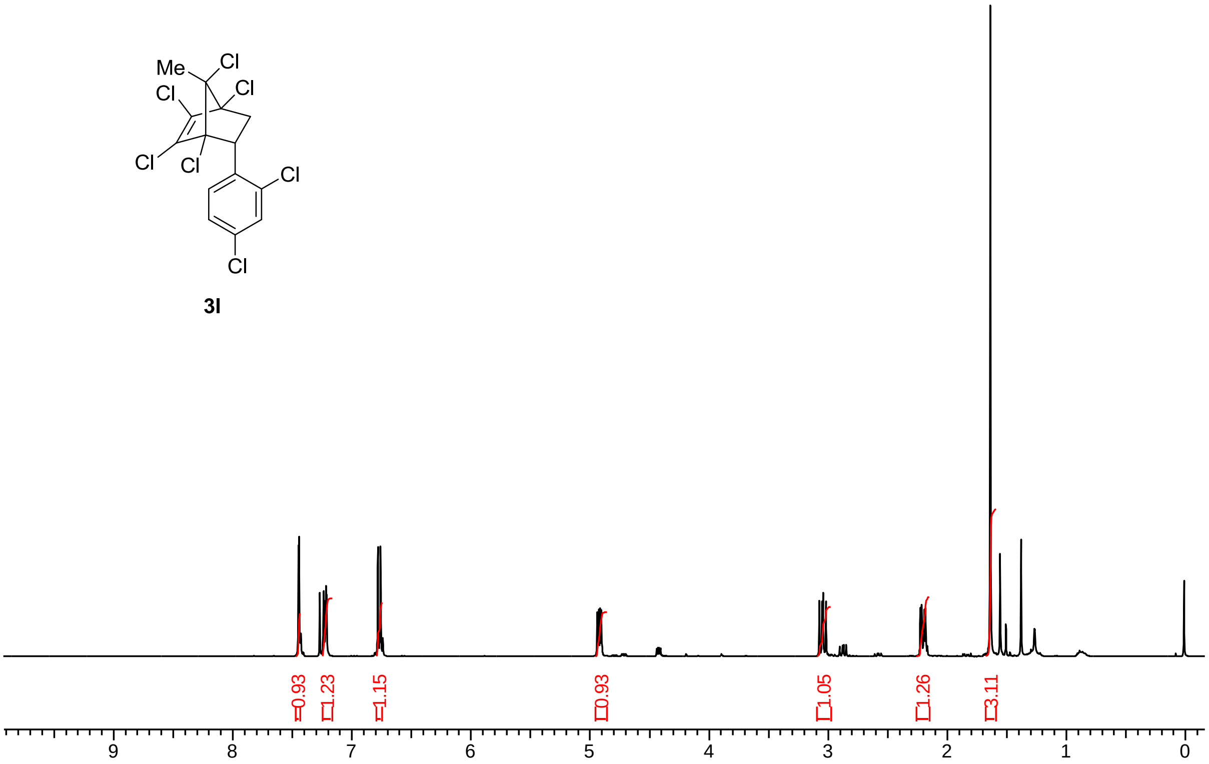

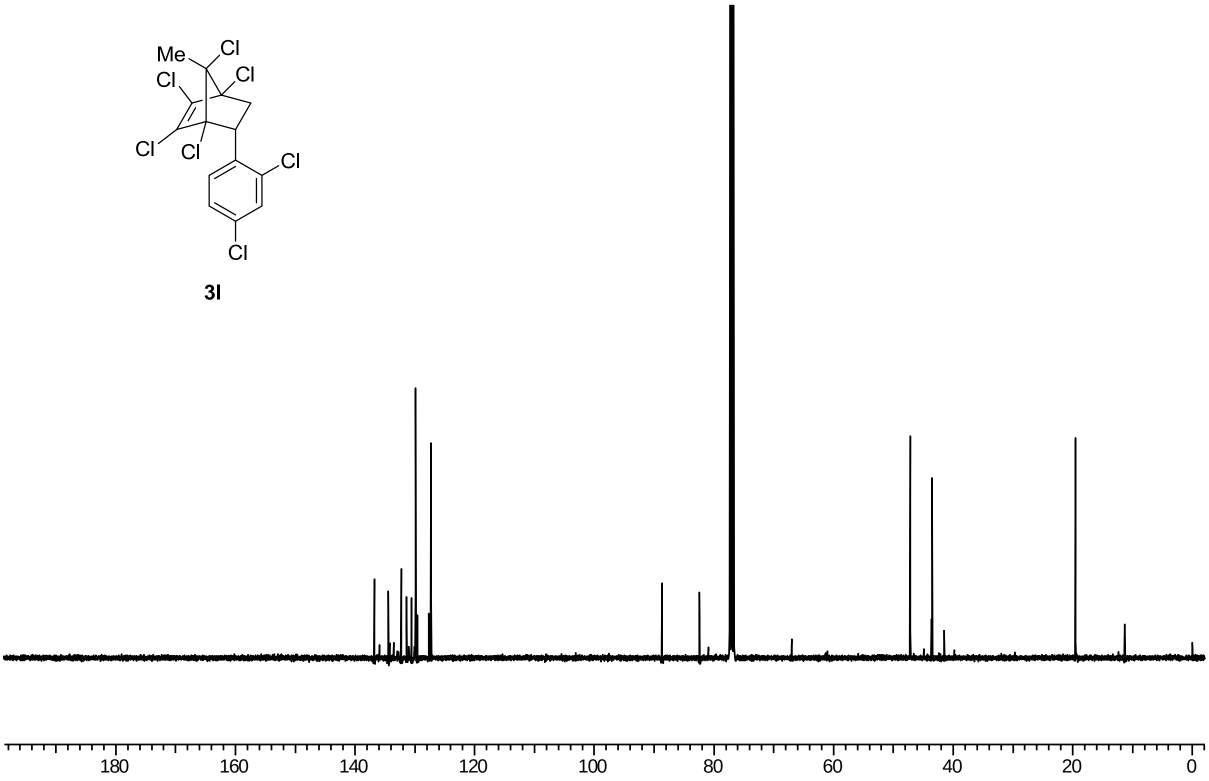

^{1}H NMR (400 MHz) and ^{13}C NMR (125 MHz) of 3m in $CDCl_3$:

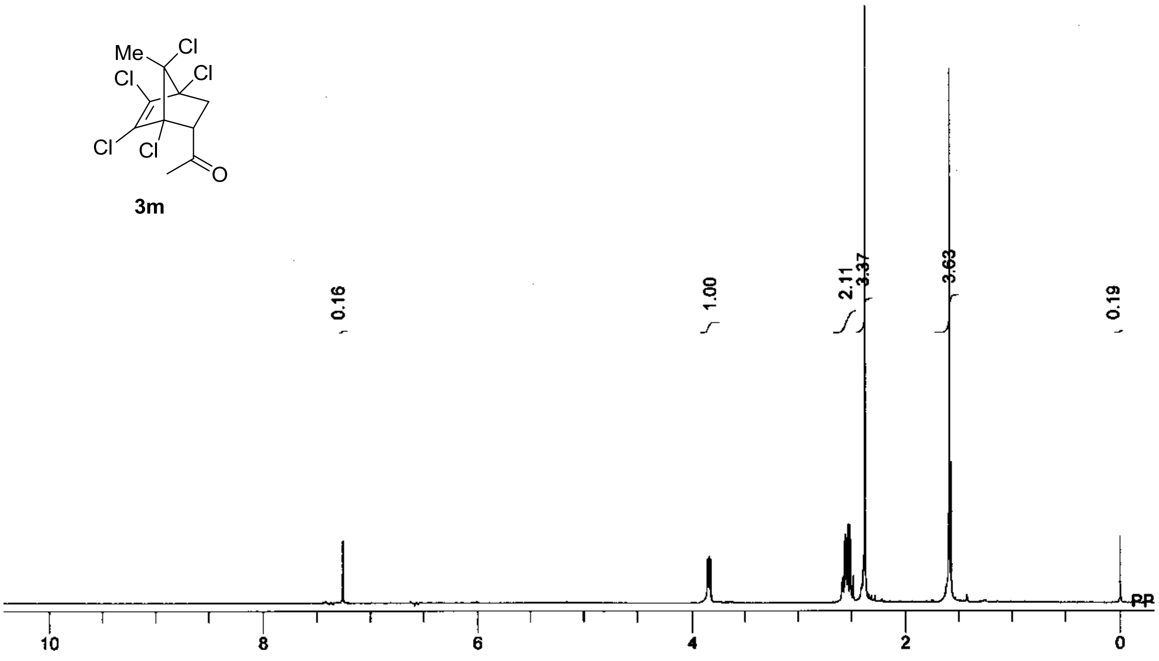

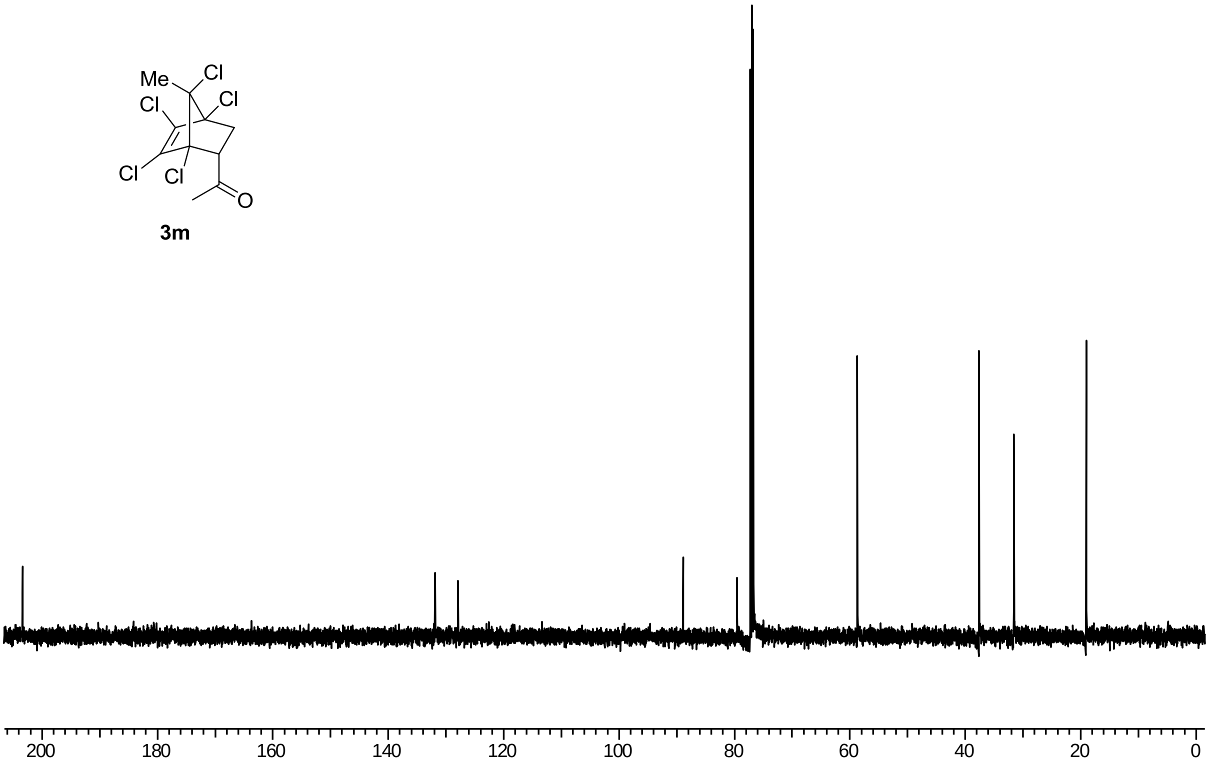

^{1}H NMR (400 MHz) and ^{13}C NMR (125 MHz) of 4m in $CDCl_3$:

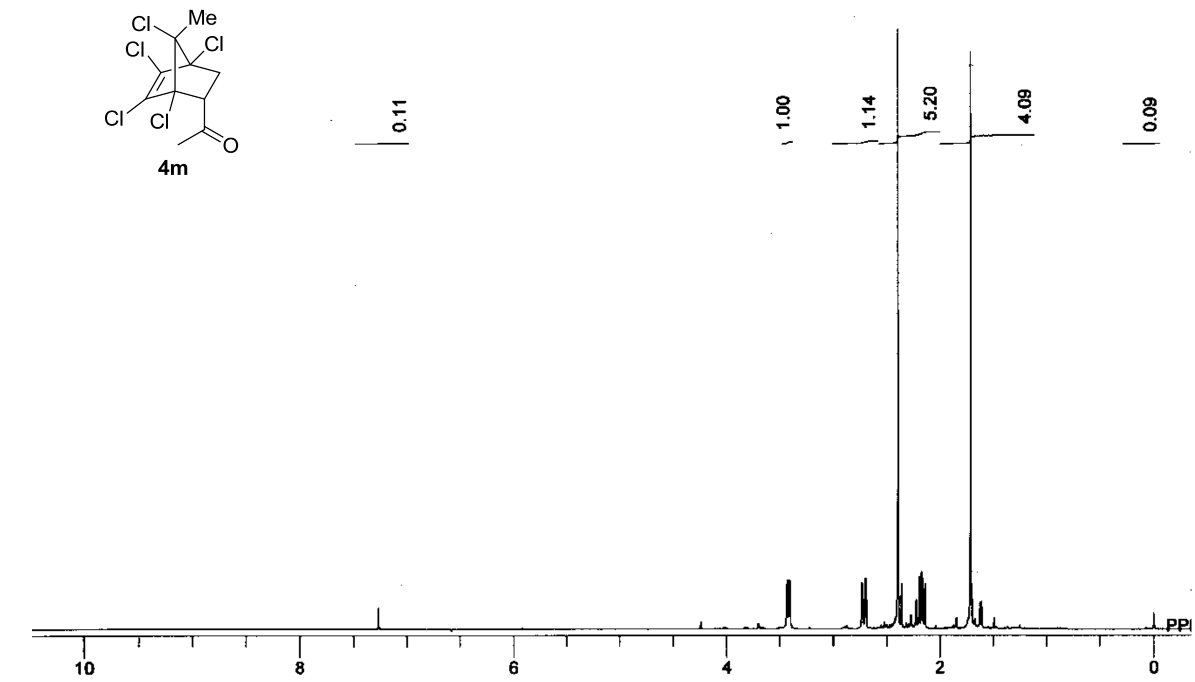

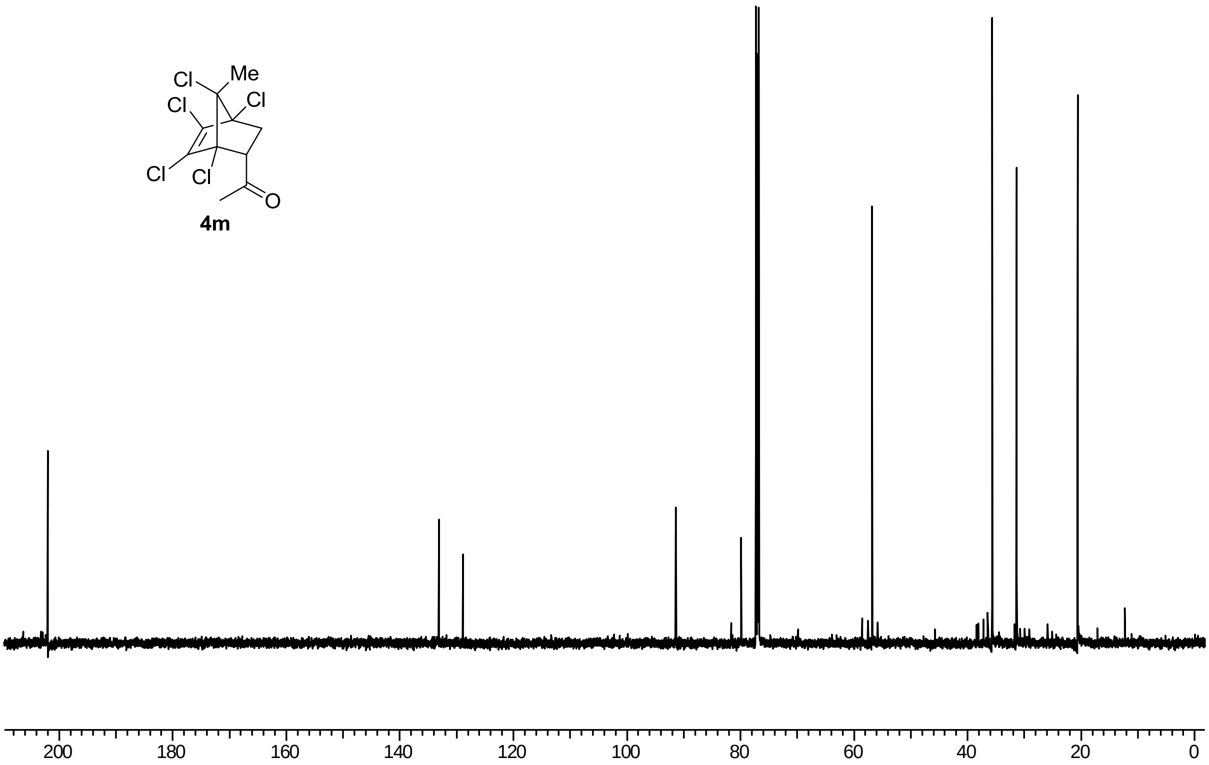

^{1}H NMR (400 MHz) and ^{13}C NMR (125 MHz) of 3n in $CDCl_3$:

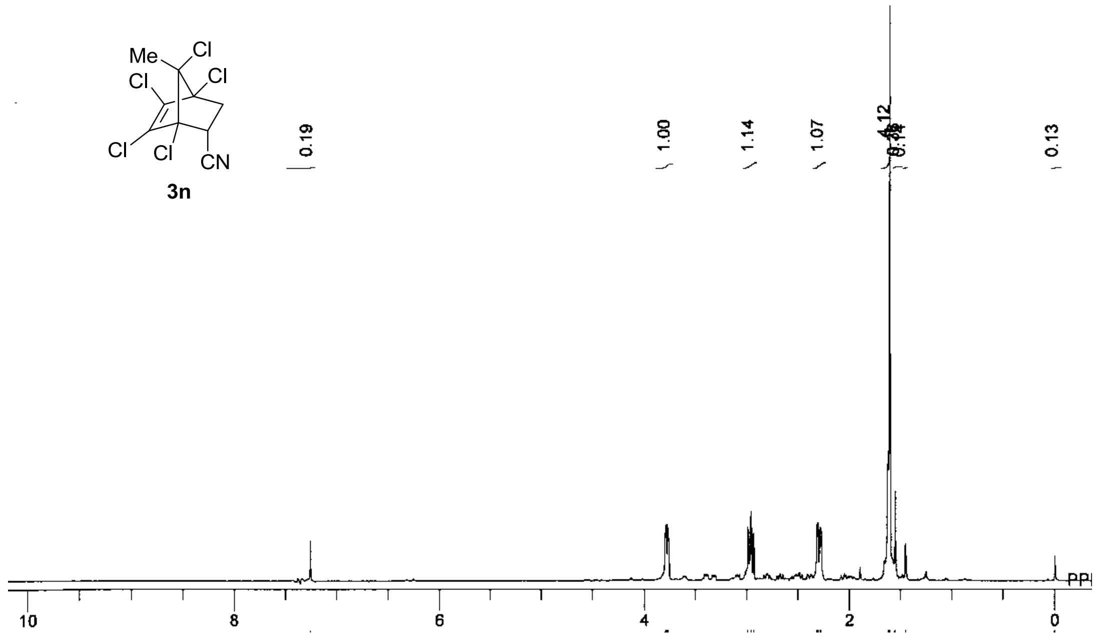

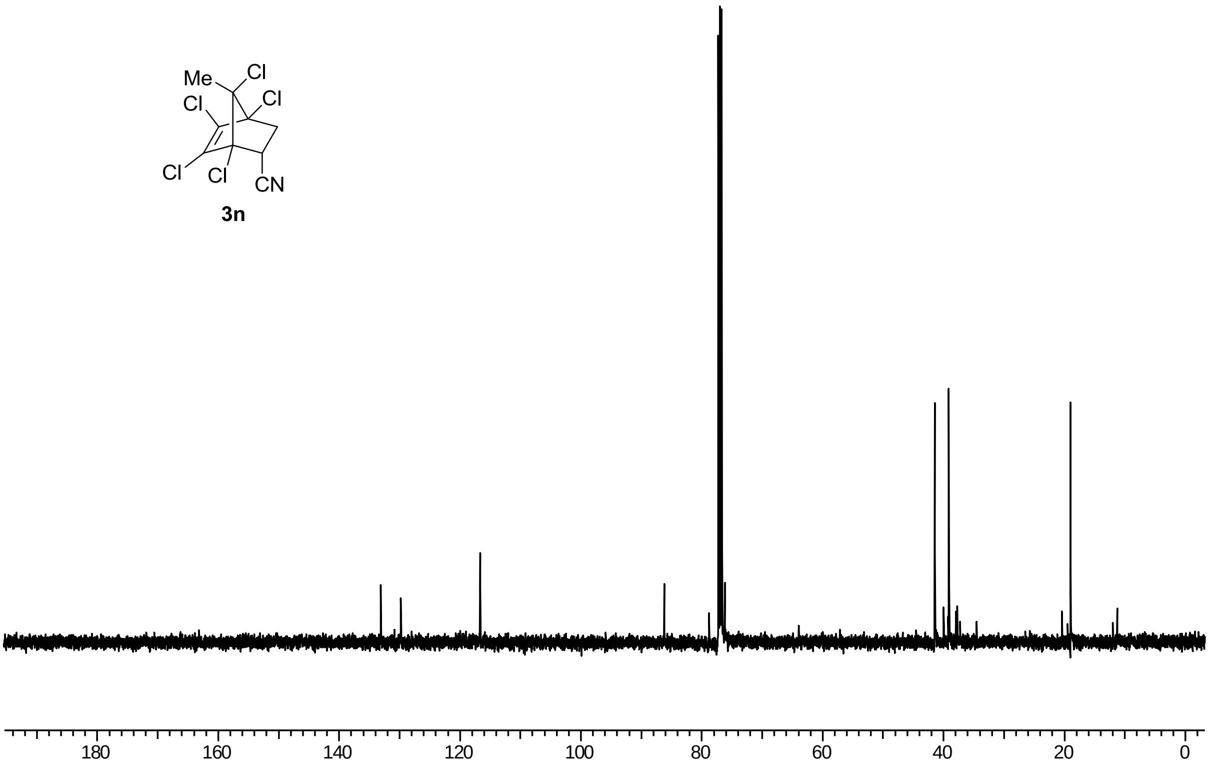

^{1}H NMR (400 MHz) and ^{13}C NMR (125 MHz) of 3o in $CDCl_3$:

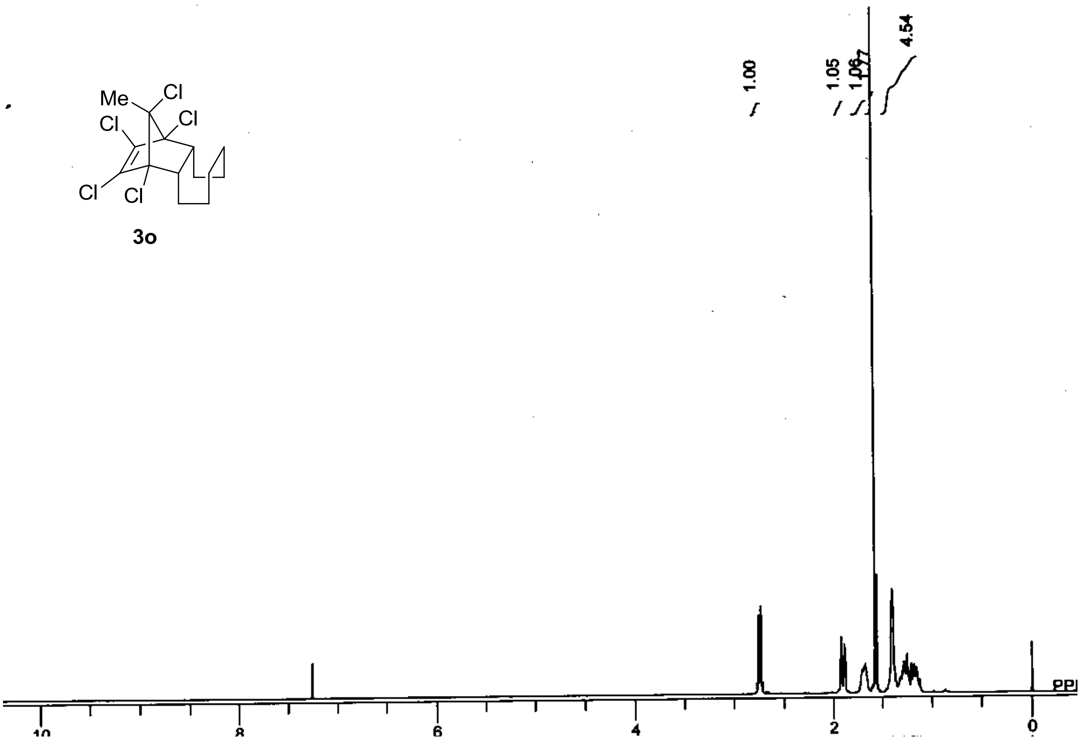

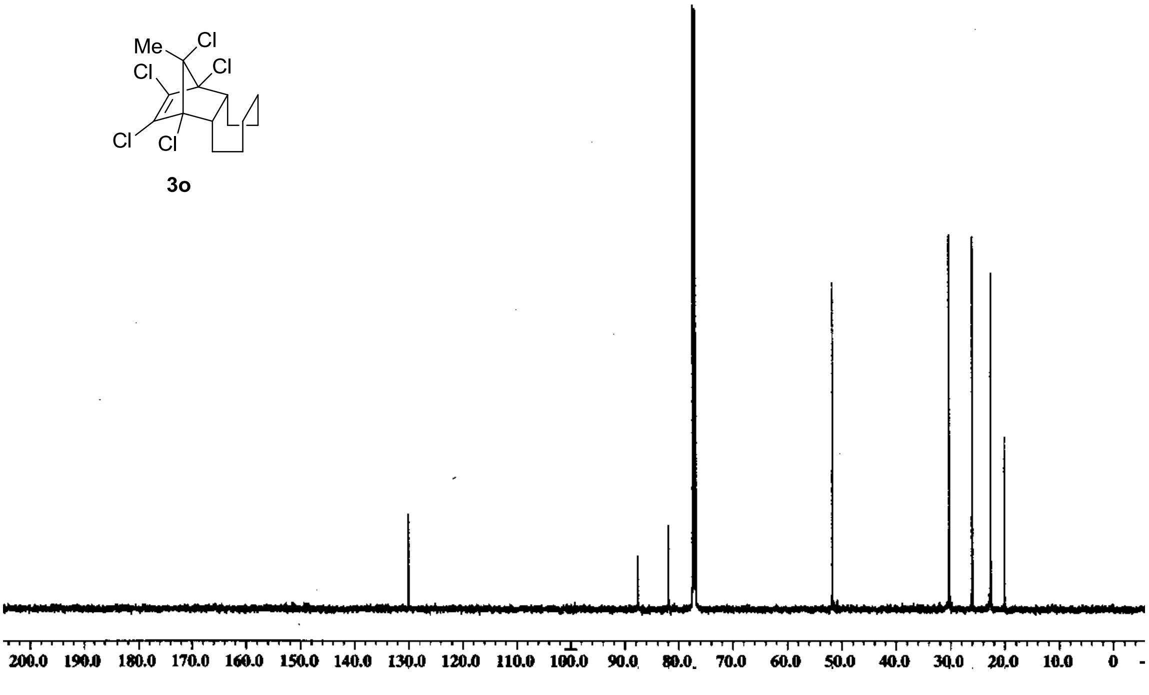

^{1}H NMR (400 MHz) and ^{13}C NMR (125 MHz) of 4o in $CDCl_3$:

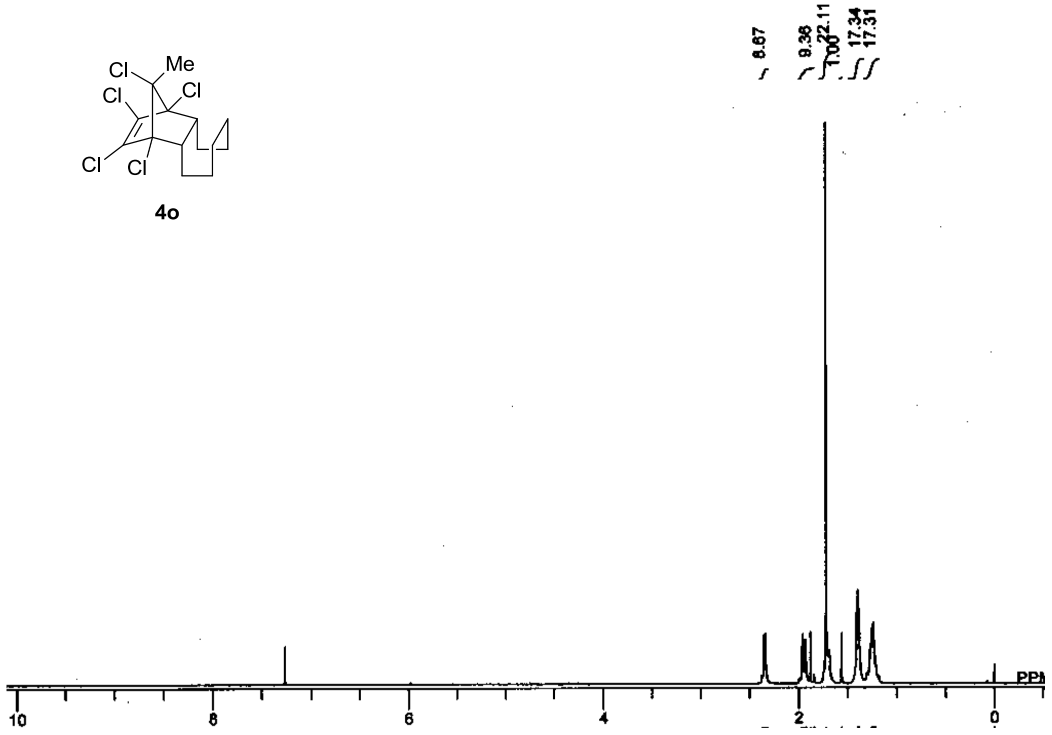

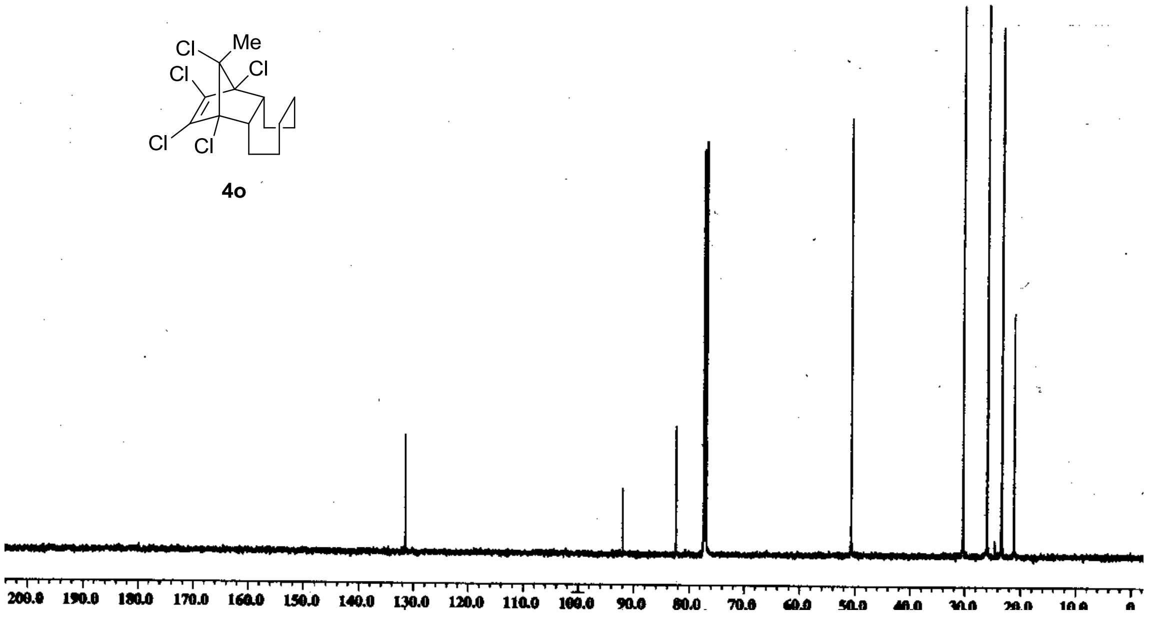

^{1}H NMR (400 MHz) and ^{13}C NMR (100 MHz) of 3p in $CDCl_3$:

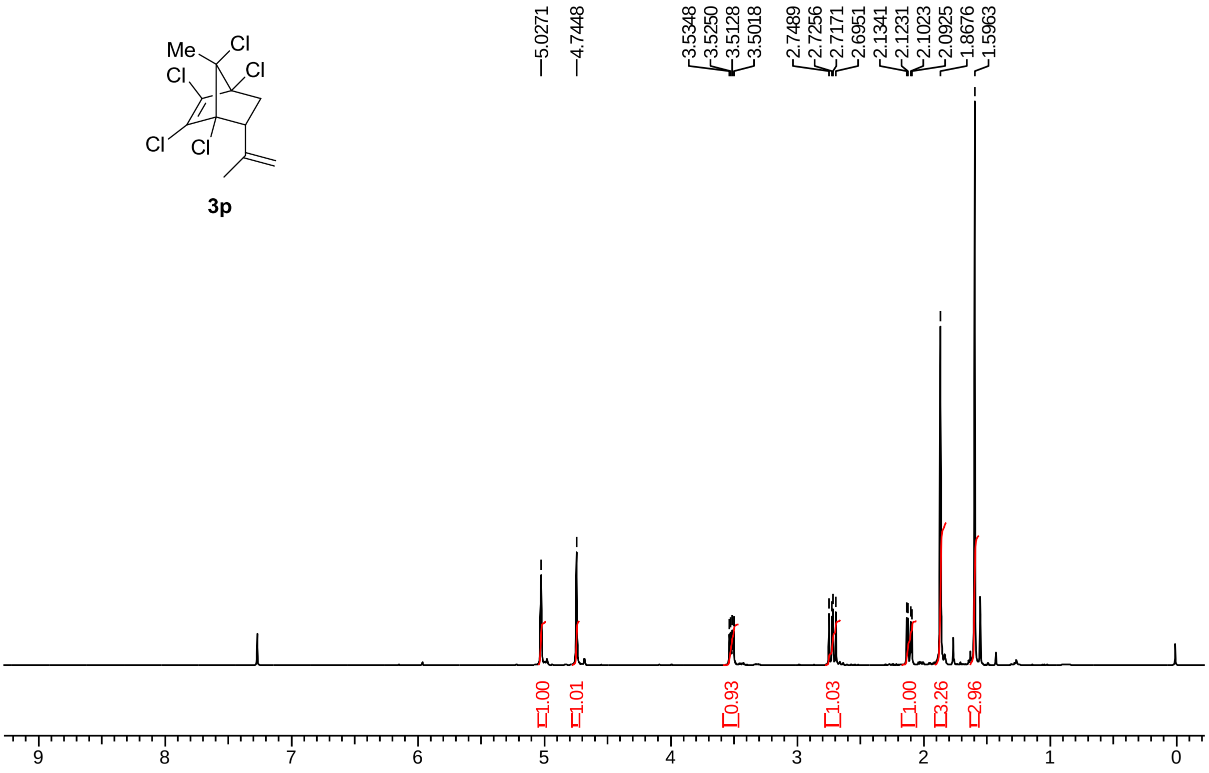

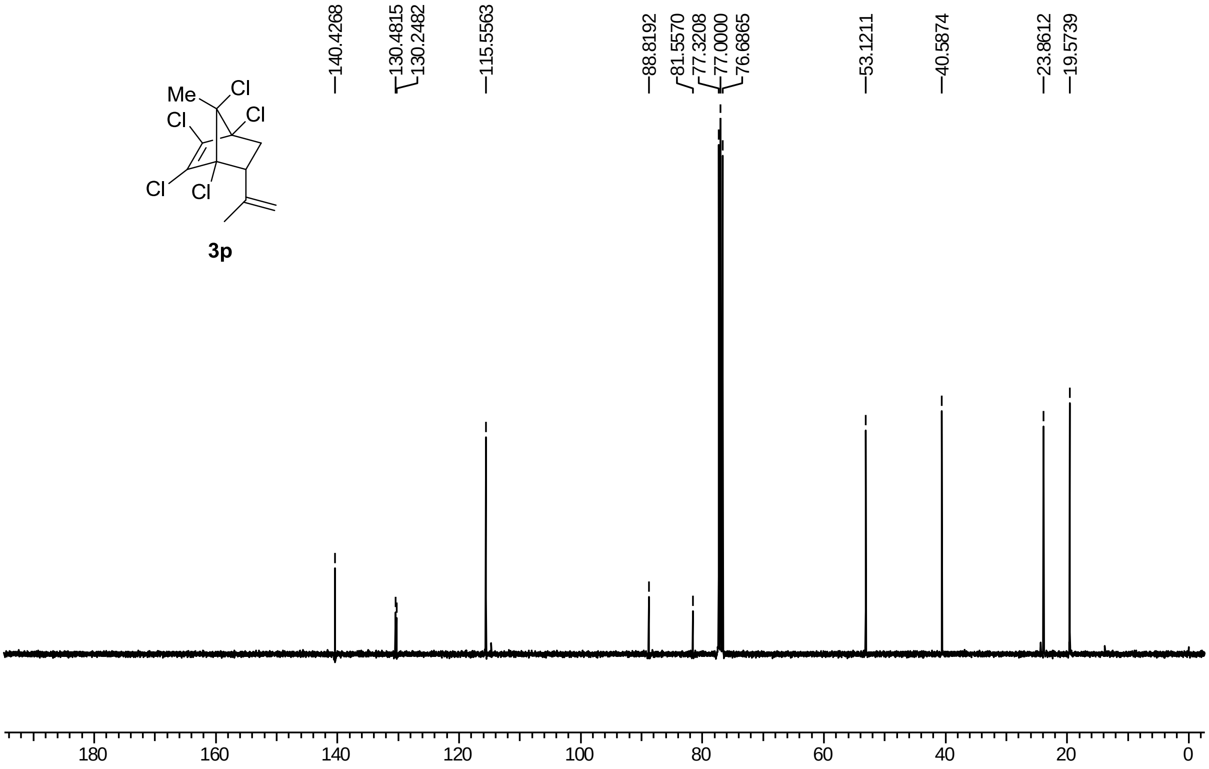

Copies of ^{1}H NMR and ^{13}C NMR spectra of nortricyclenes 5:

^{1}H NMR (400 MHz) and ^{13}C NMR (125 MHz) of 5a in $CDCl_3$:

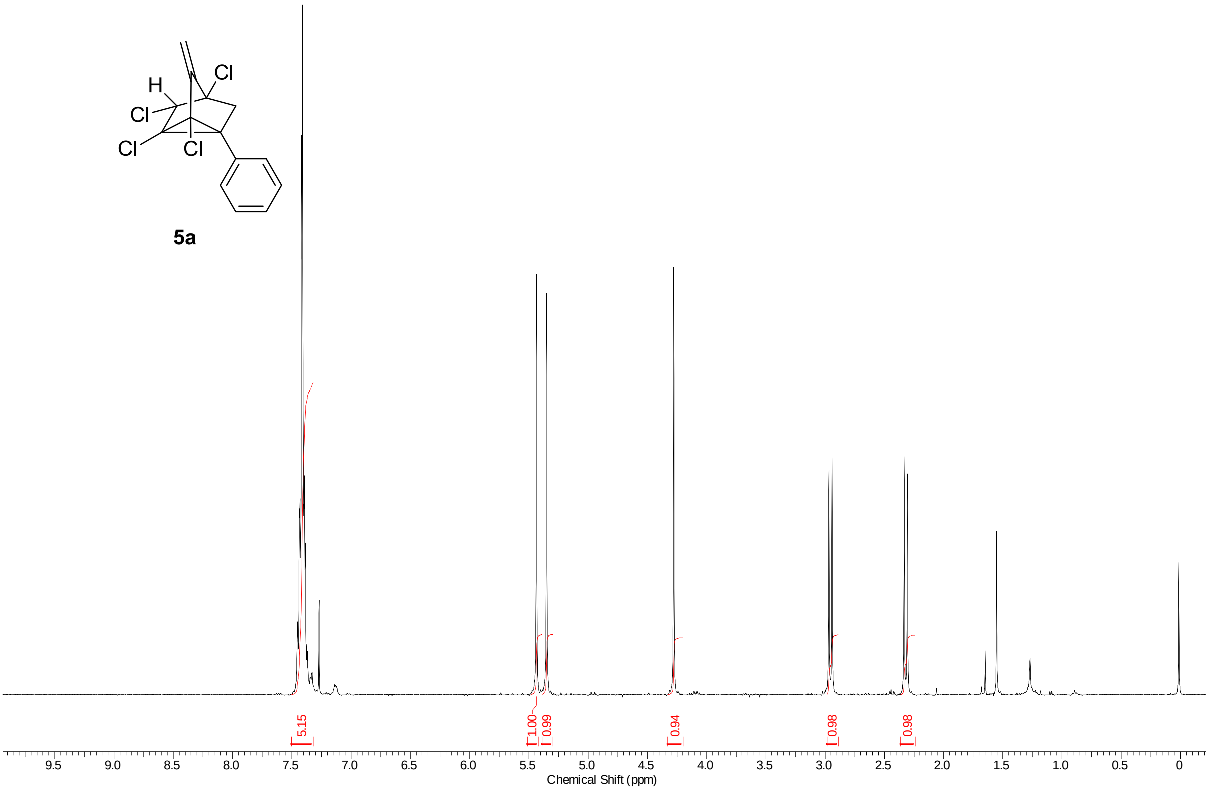

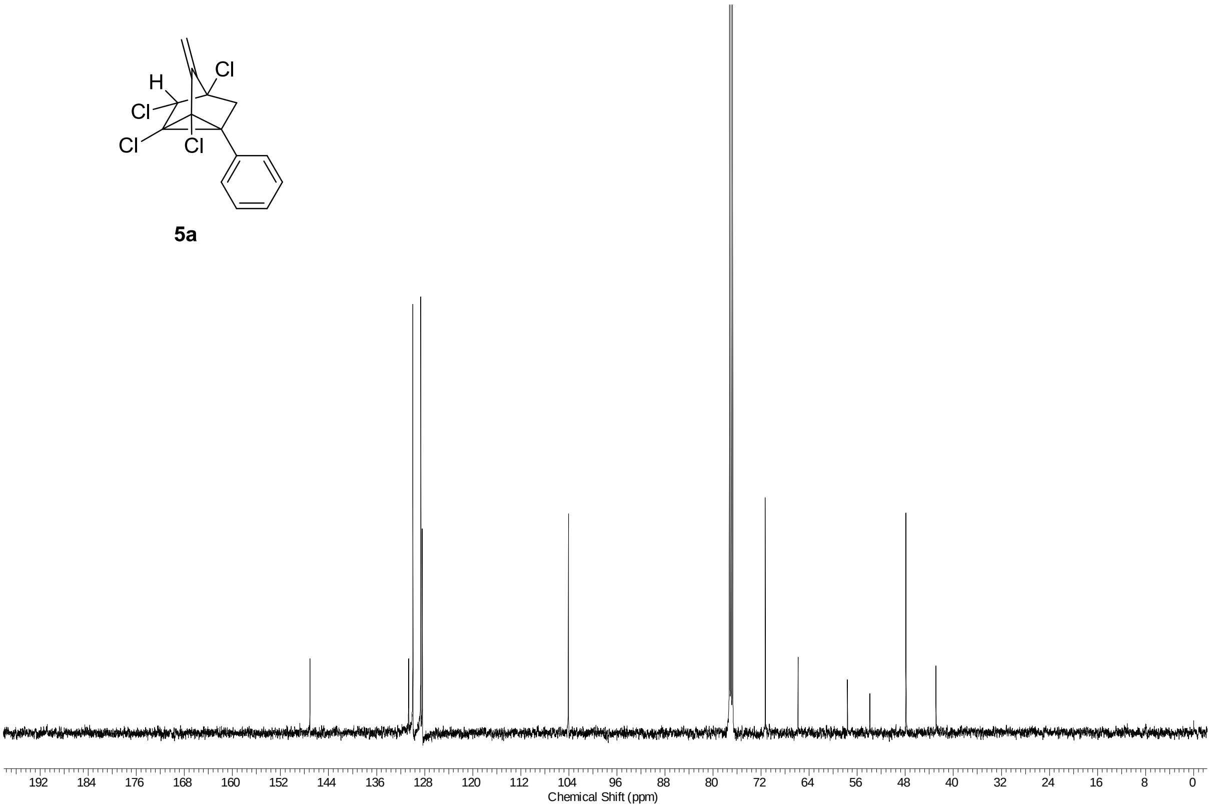

^{1}H NMR (400 MHz) and ^{13}C NMR (125 MHz) of 5b in $CDCl_3$:

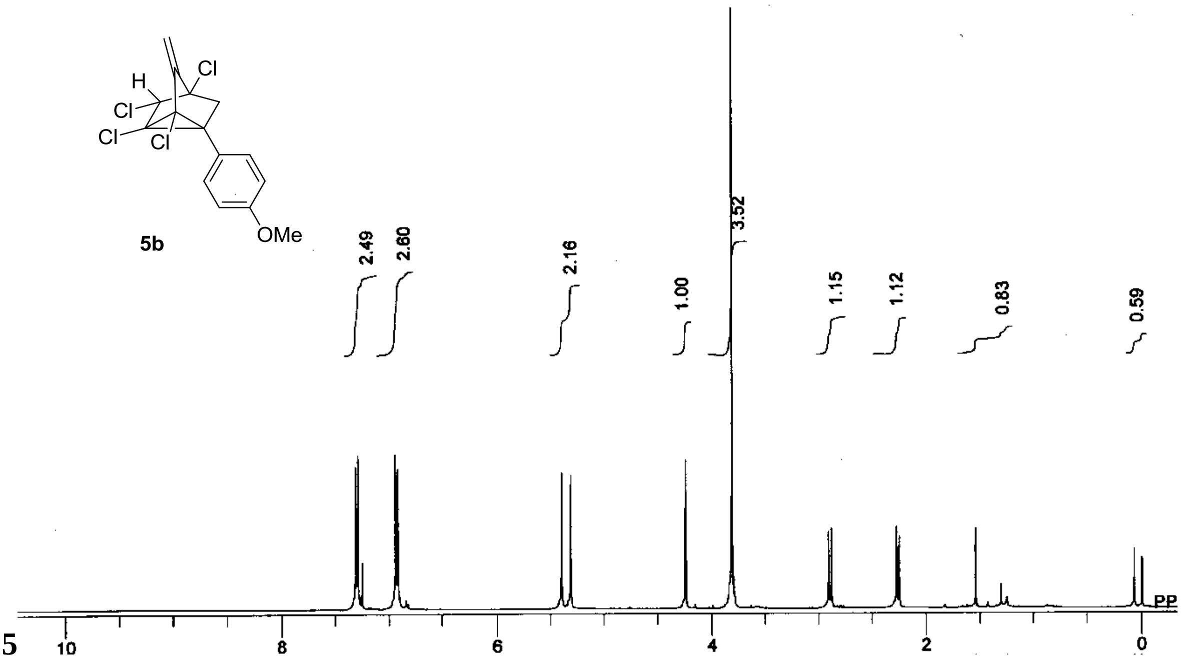

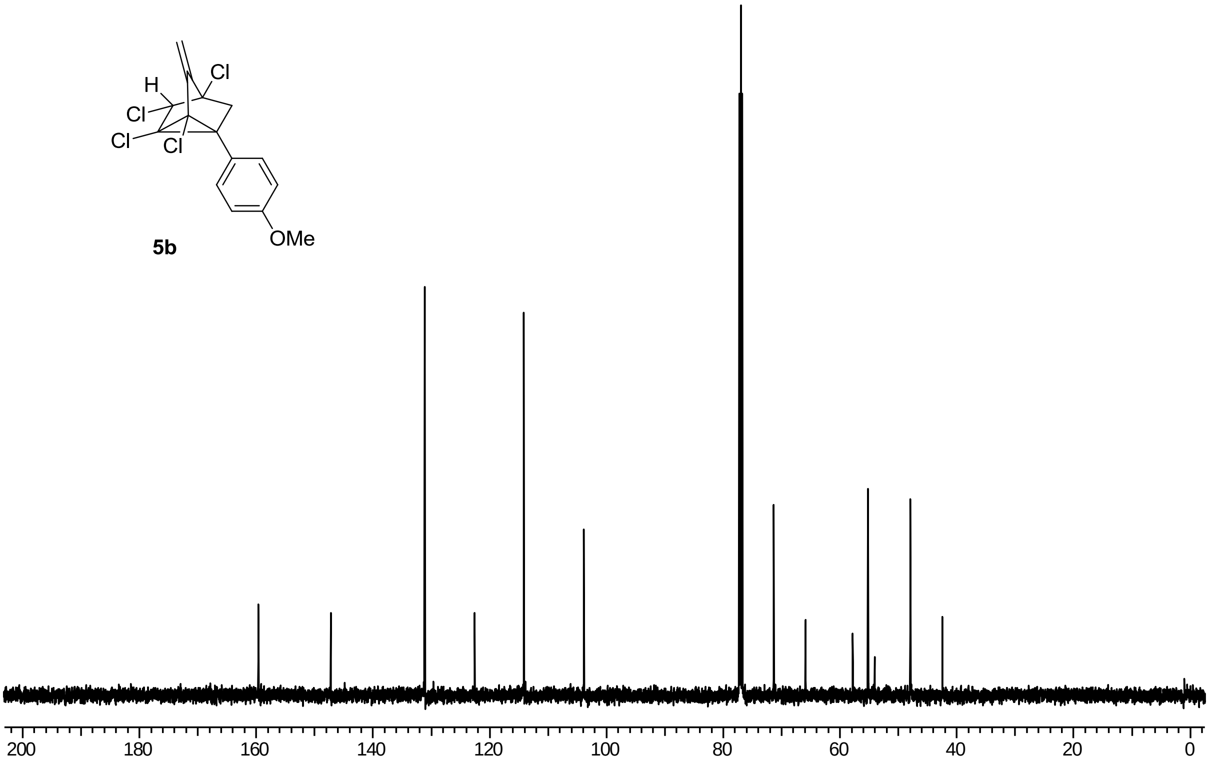

^{1}H NMR (400 MHz) and ^{13}C NMR (125 MHz) of 5b and 6a in $CDCl_3$:

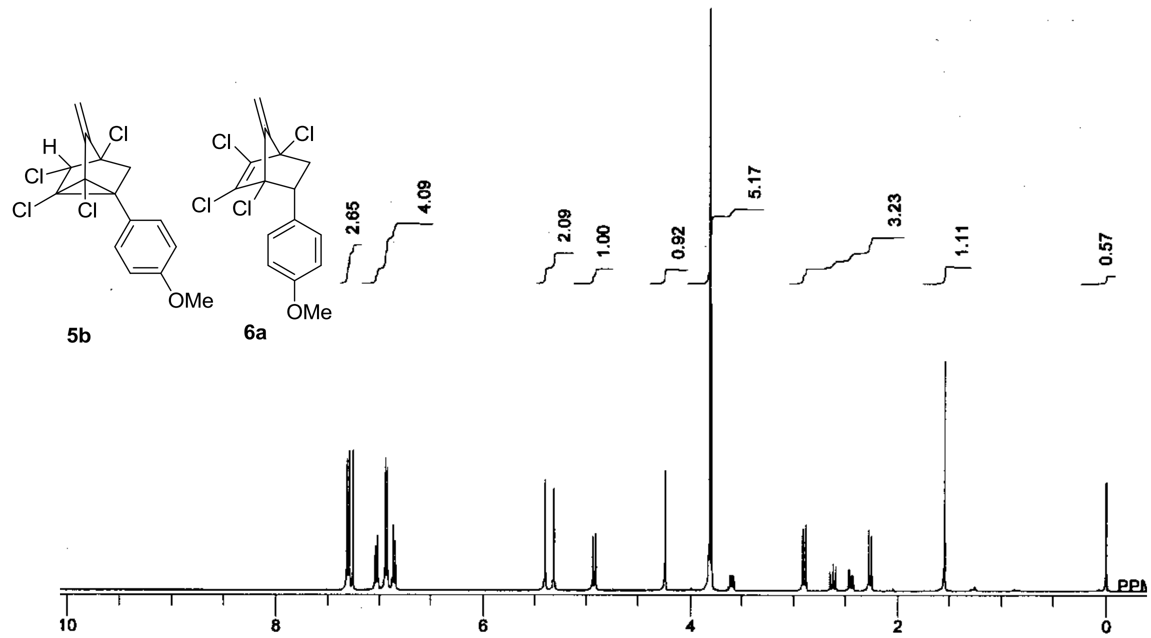

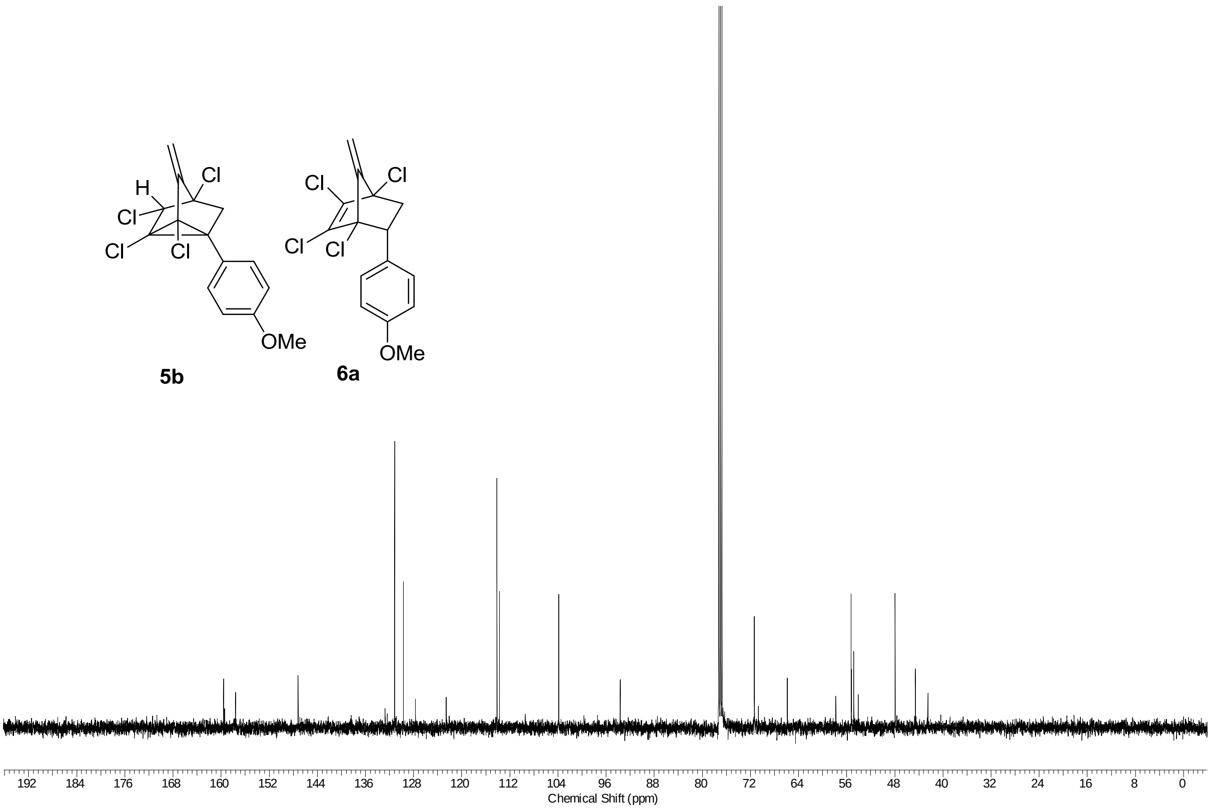

^{1}H NMR (400 MHz) and ^{13}C NMR (100 MHz) of 5c in $CDCl_3$:

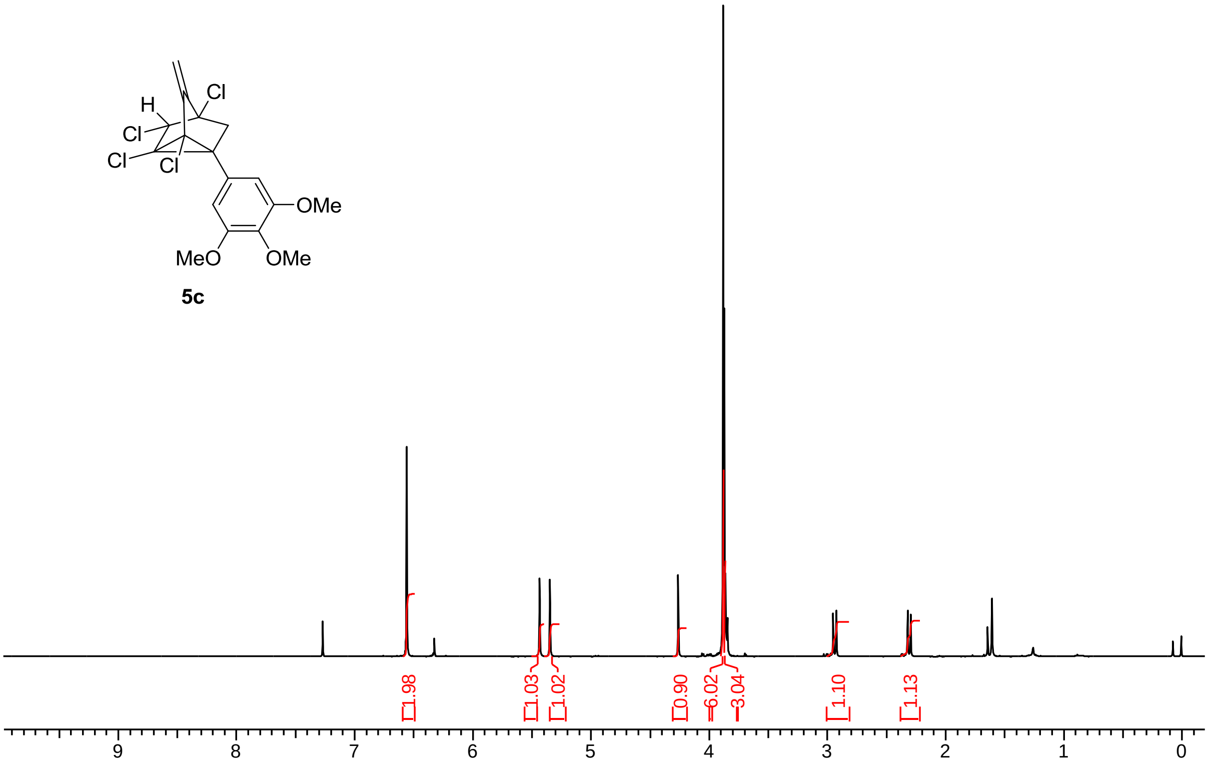

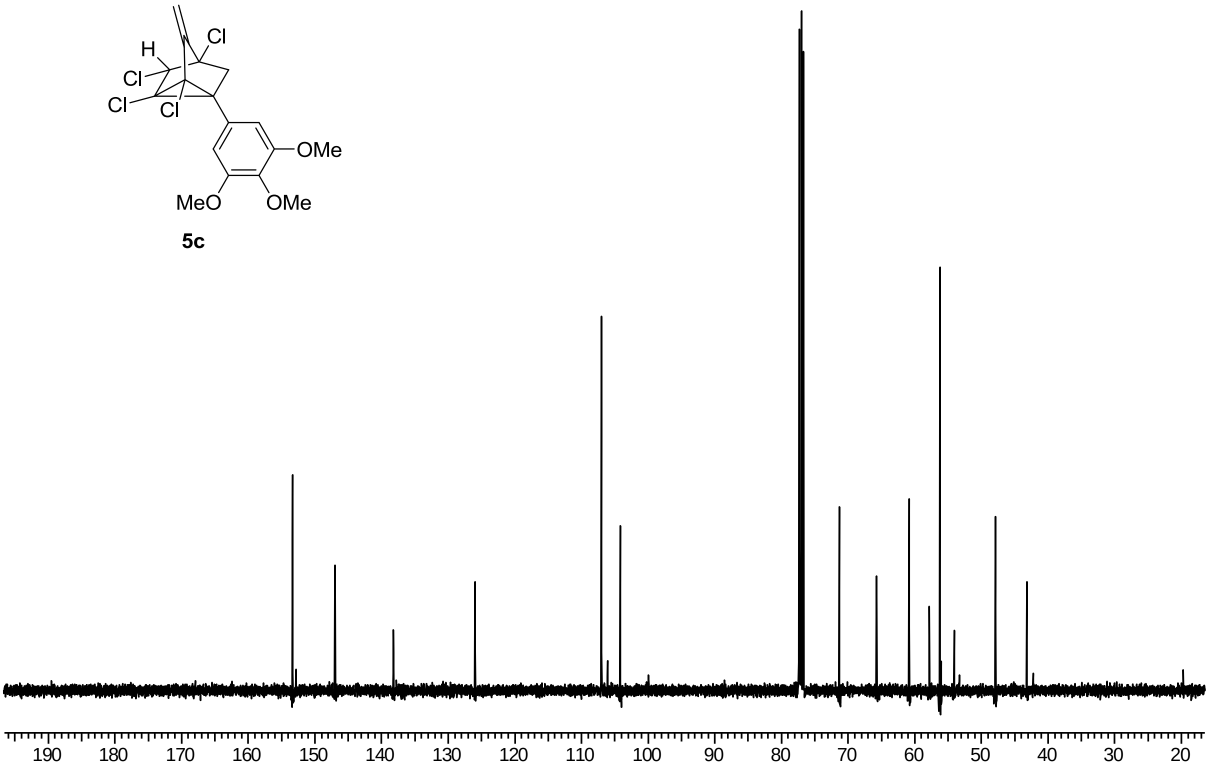

^{1}H NMR (400 MHz) and ^{13}C NMR (100 MHz) of 5d in $CDCl_3$:

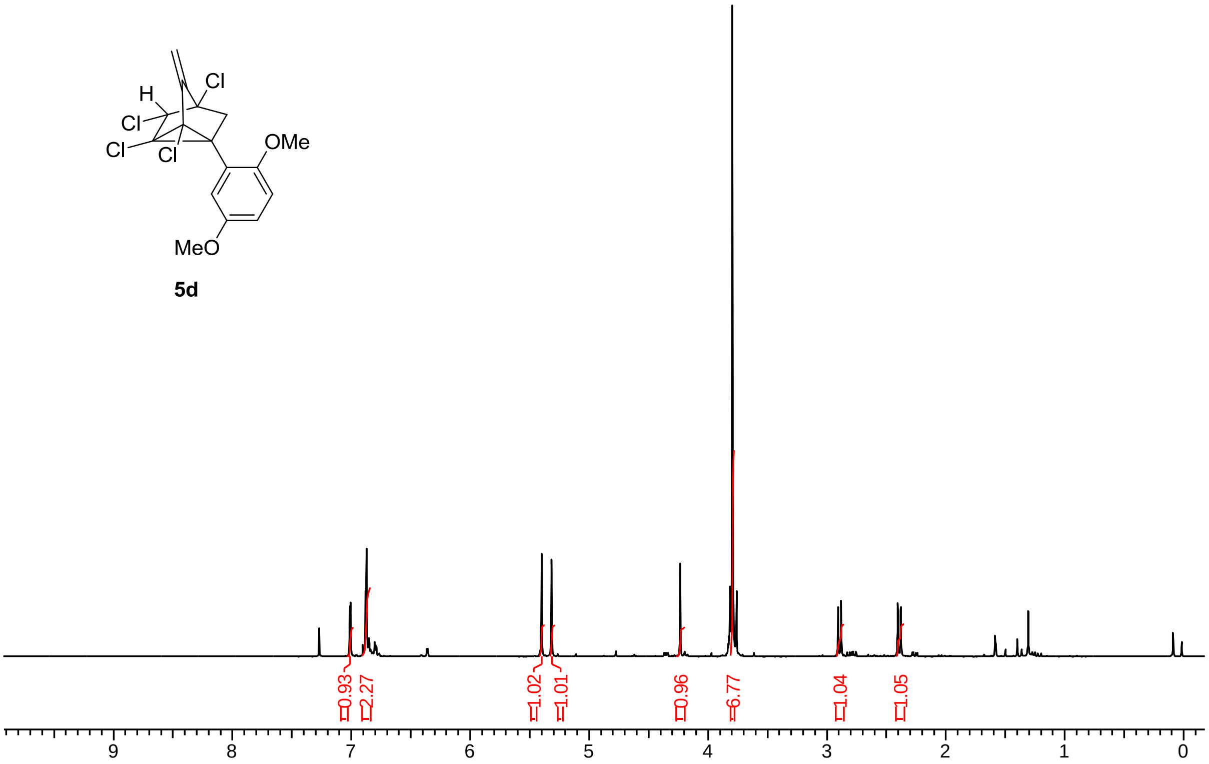

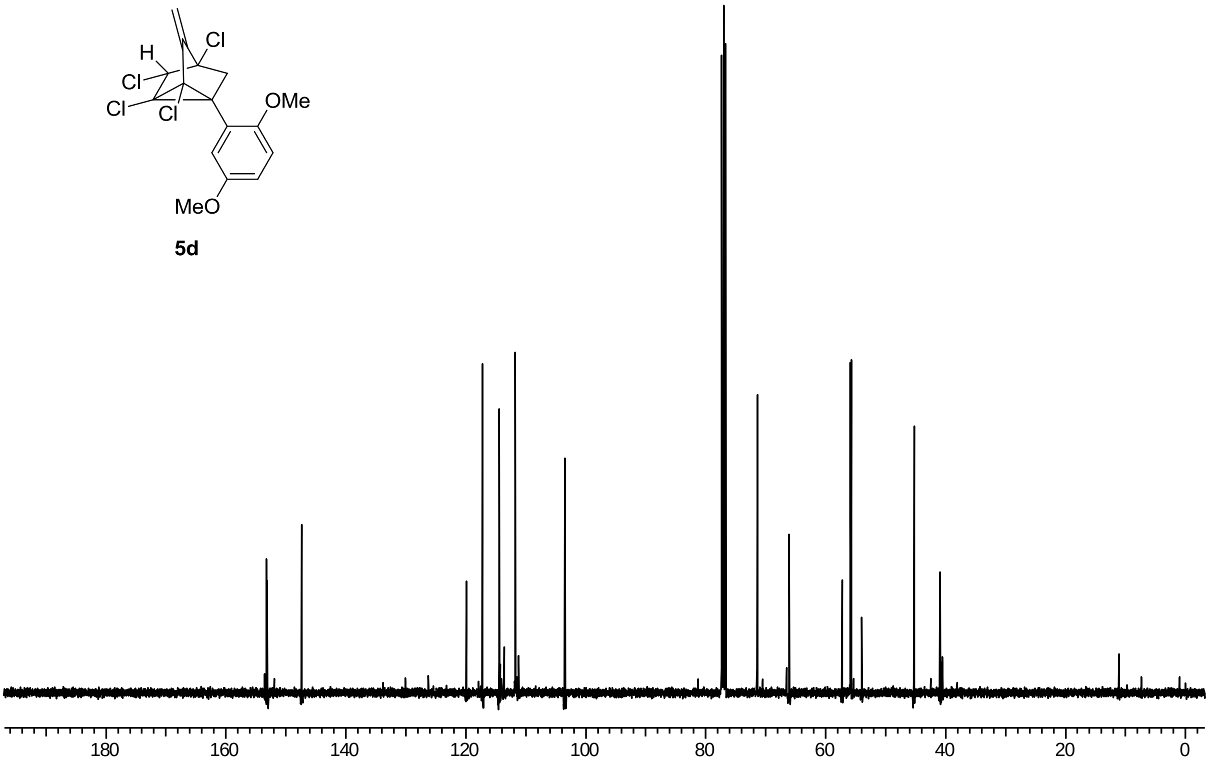

^{1}H NMR (500 MHz) and ^{13}C NMR (125 MHz) of 5g in $CDCl_3$:

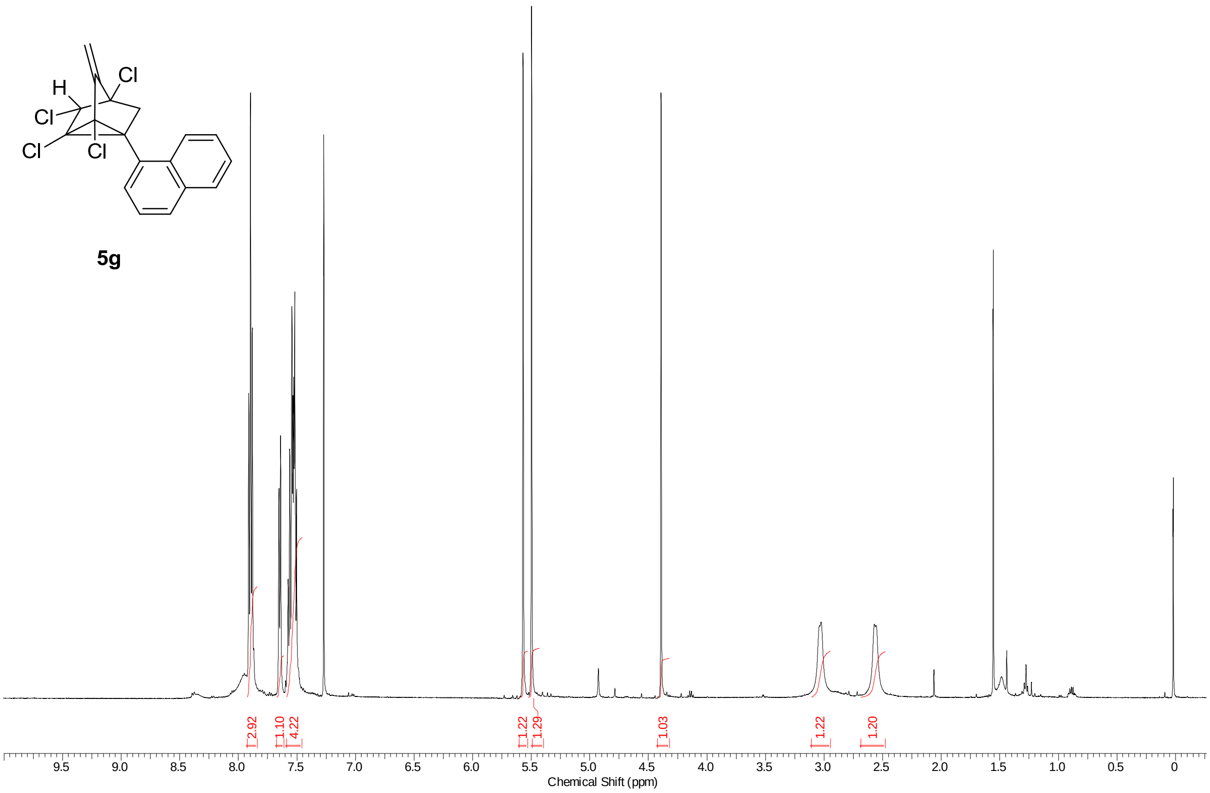

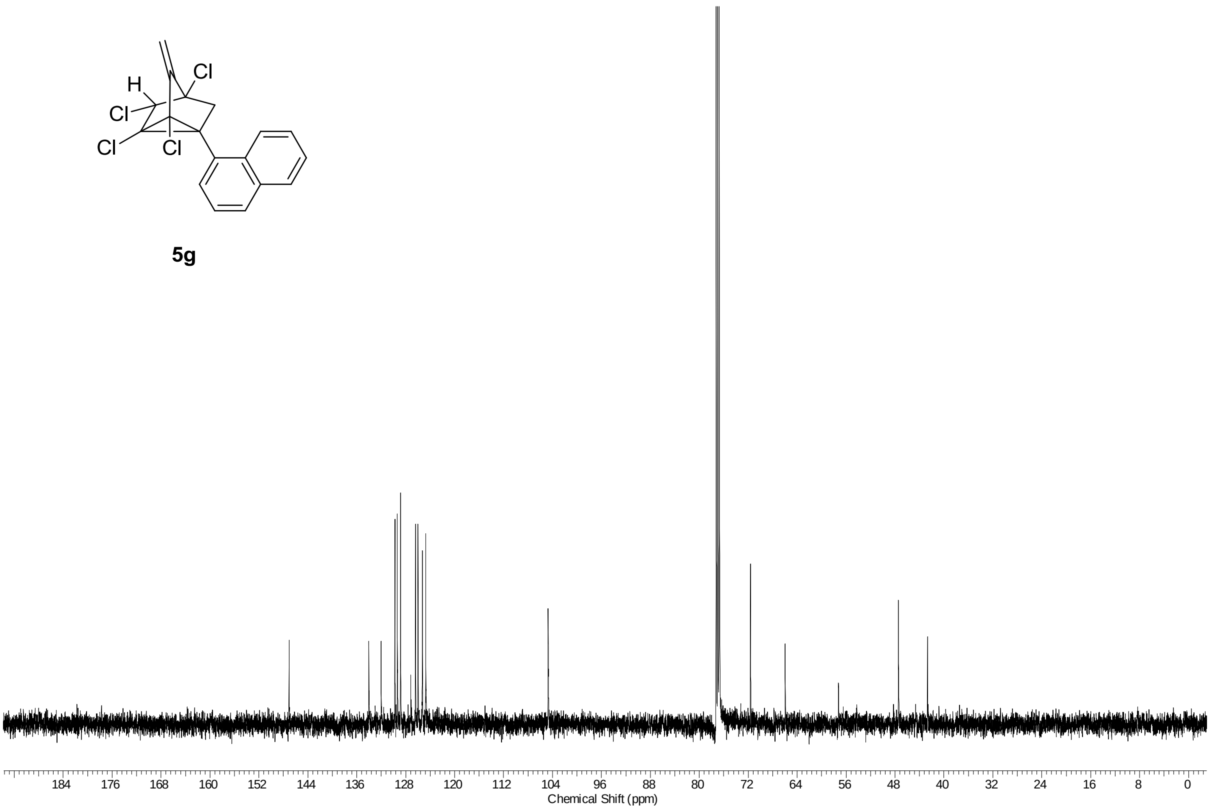

^{1}H NMR (400 MHz) and ^{13}C NMR (100 MHz) of 5f in $CDCl_3$:

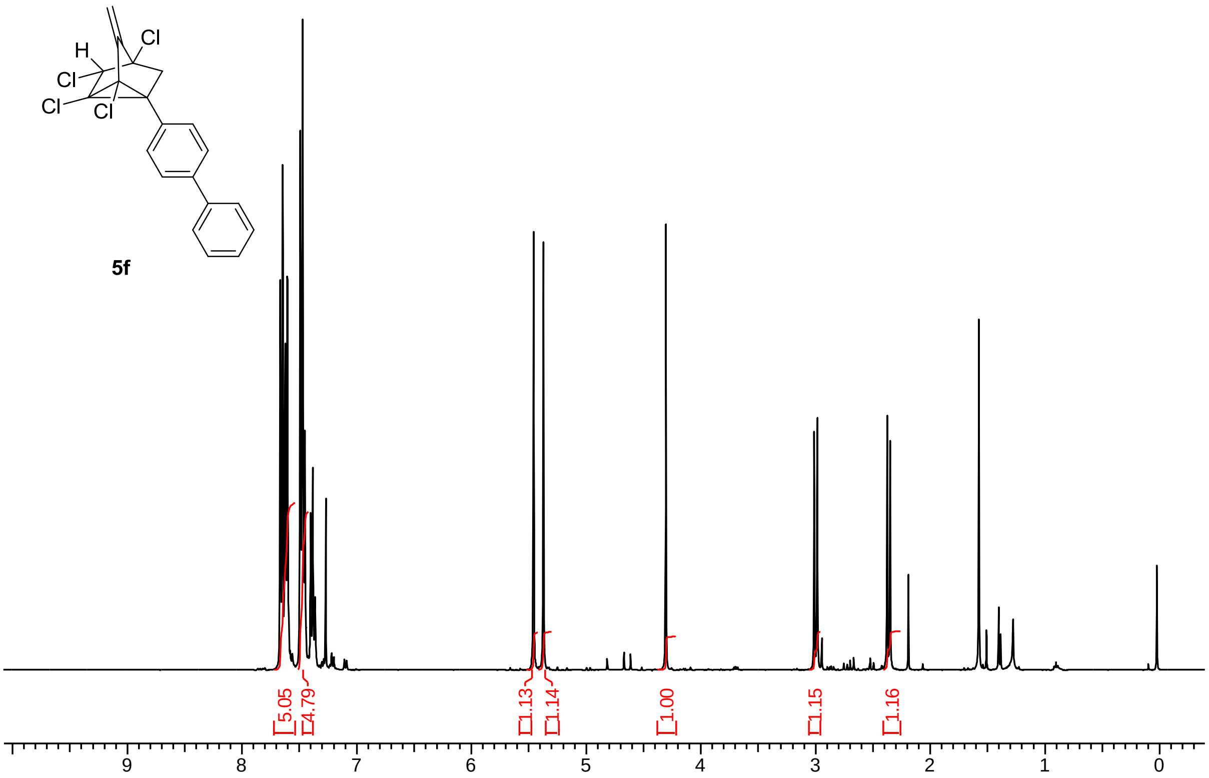

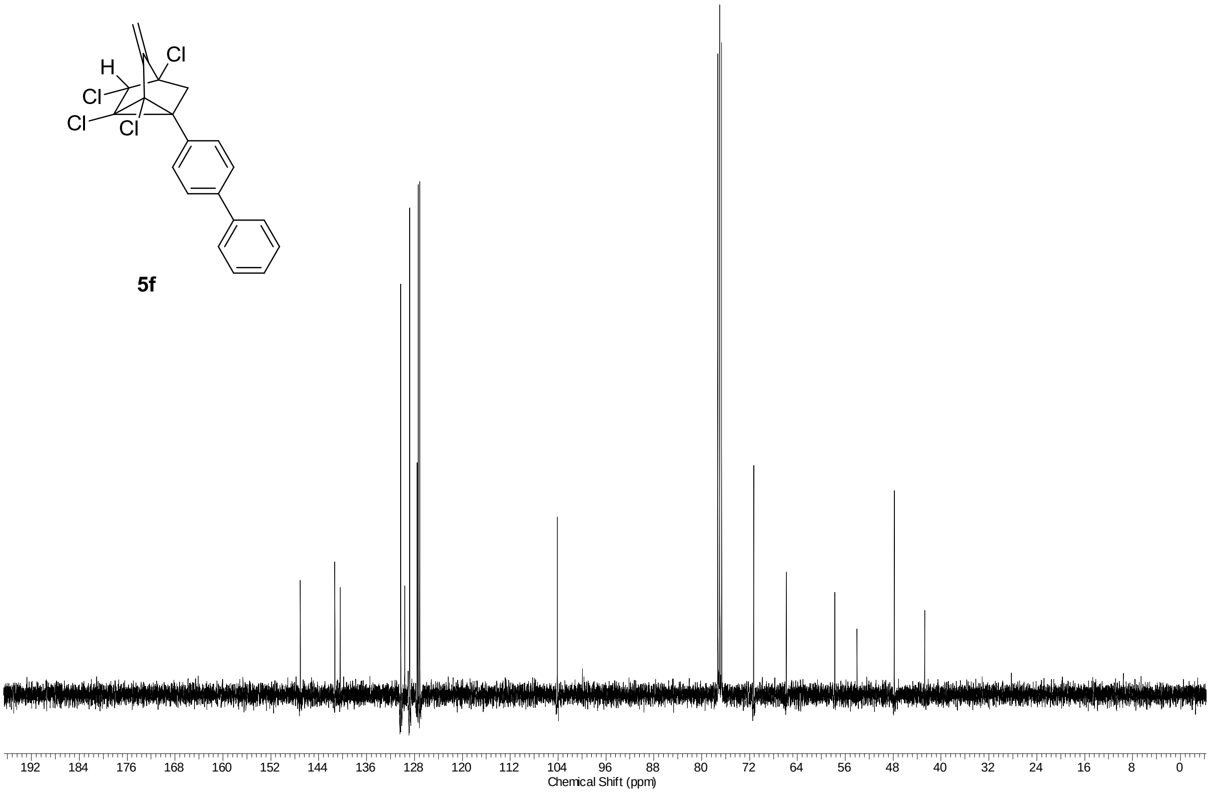

^{1}H NMR (400 MHz) and ^{13}C NMR (100 MHz) of 5h in $CDCl_3$:

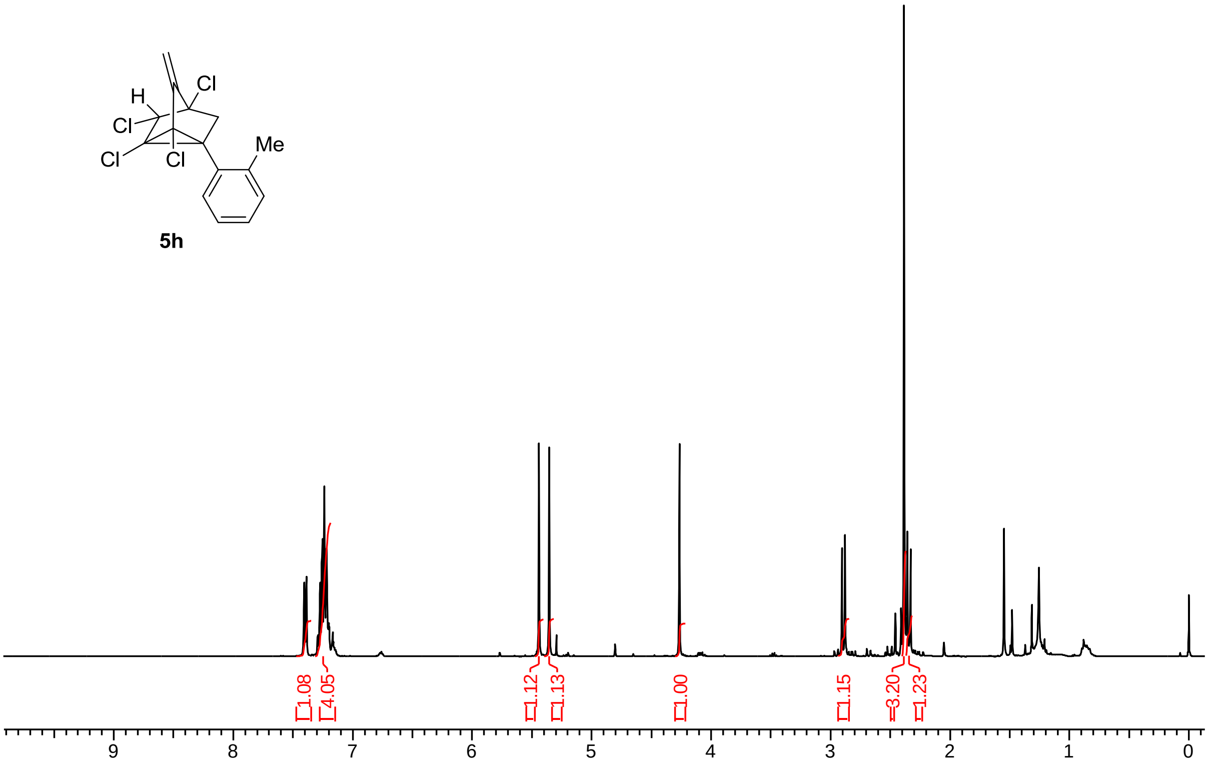

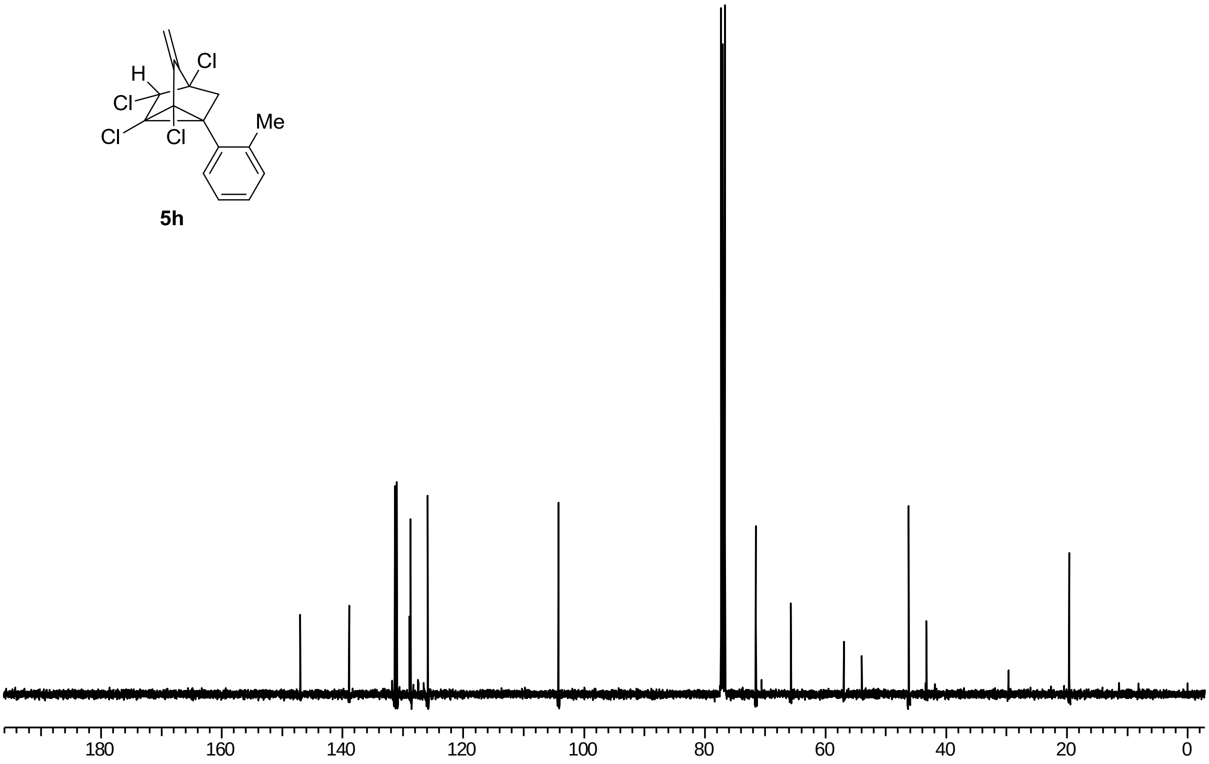

^{1}H NMR (400 MHz) and ^{13}C NMR (100 MHz) of 5h and 6b in $CDCl_3$:

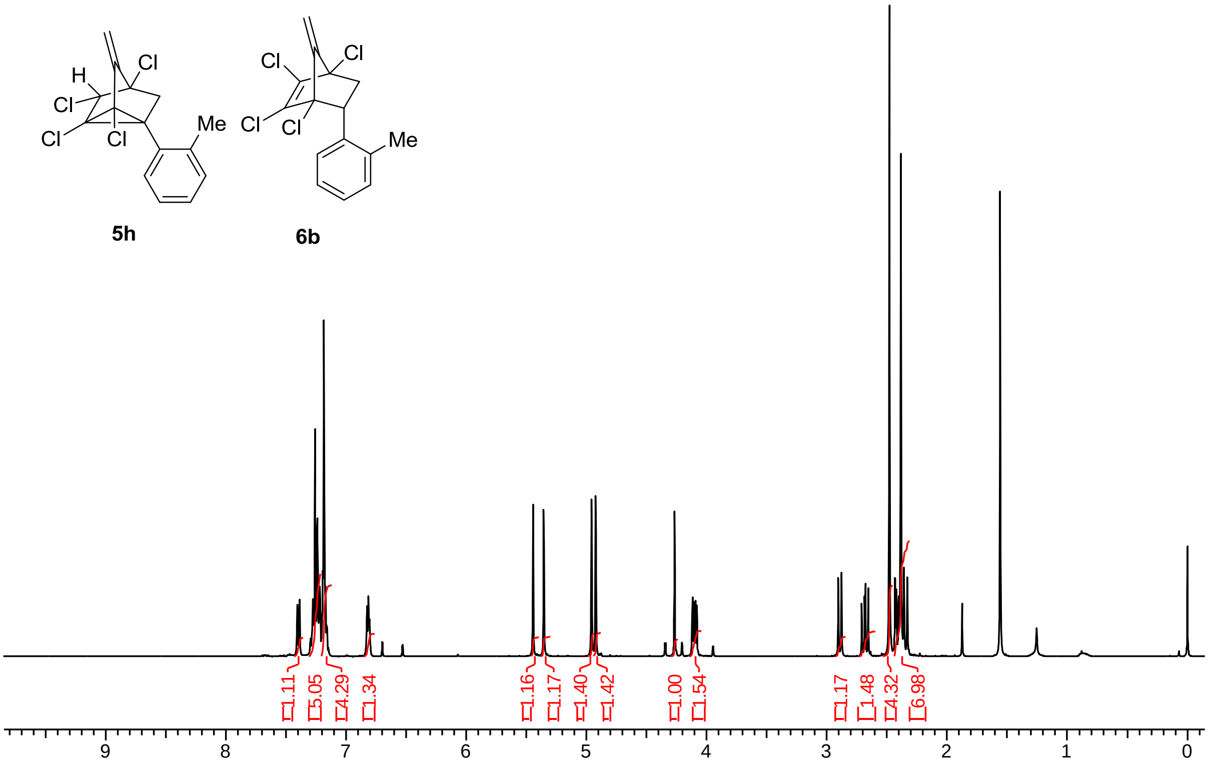

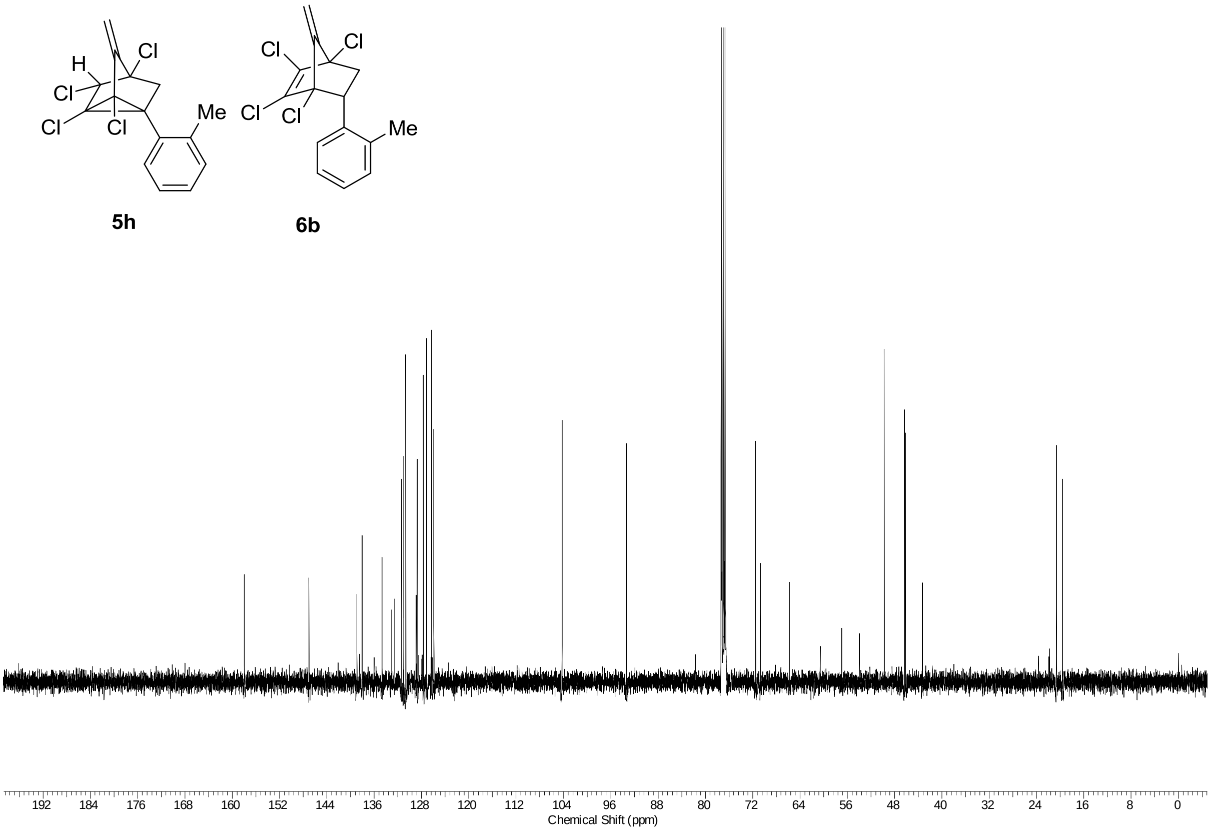

^{1}H NMR (400 MHz) and ^{13}C NMR (100 MHz) of 5i in $CDCl_3$:

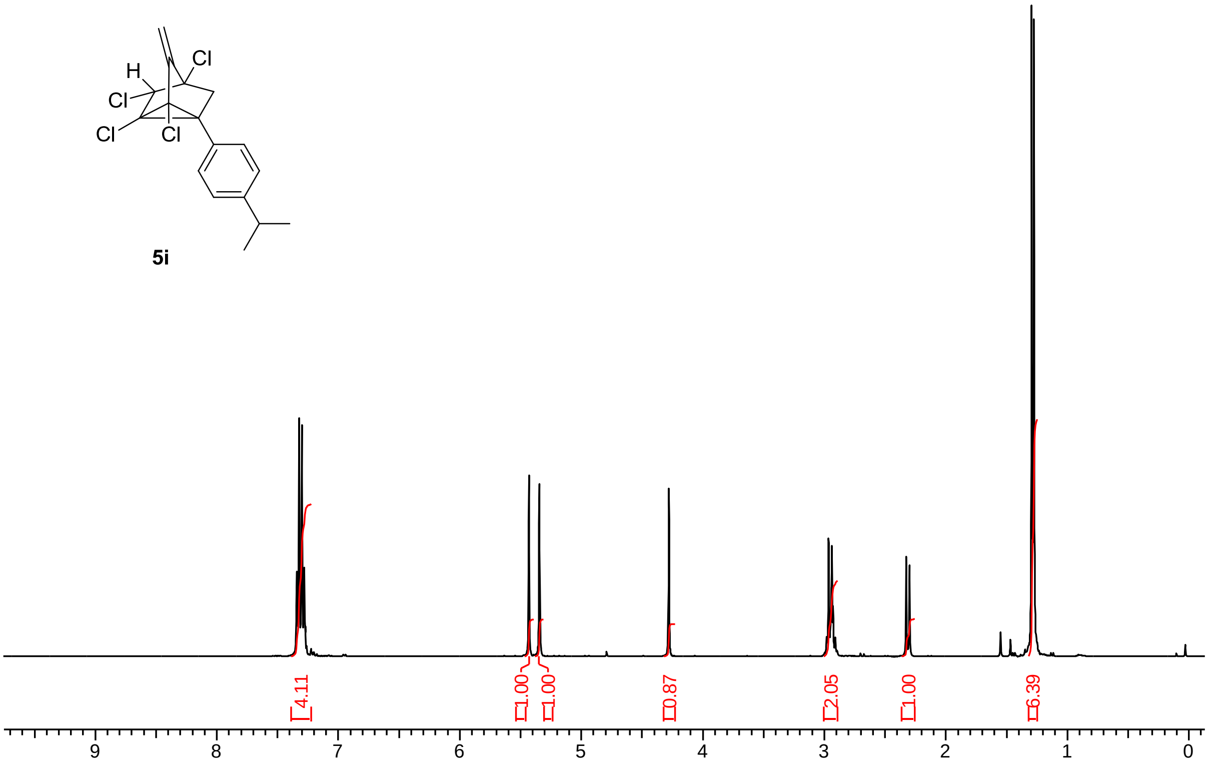

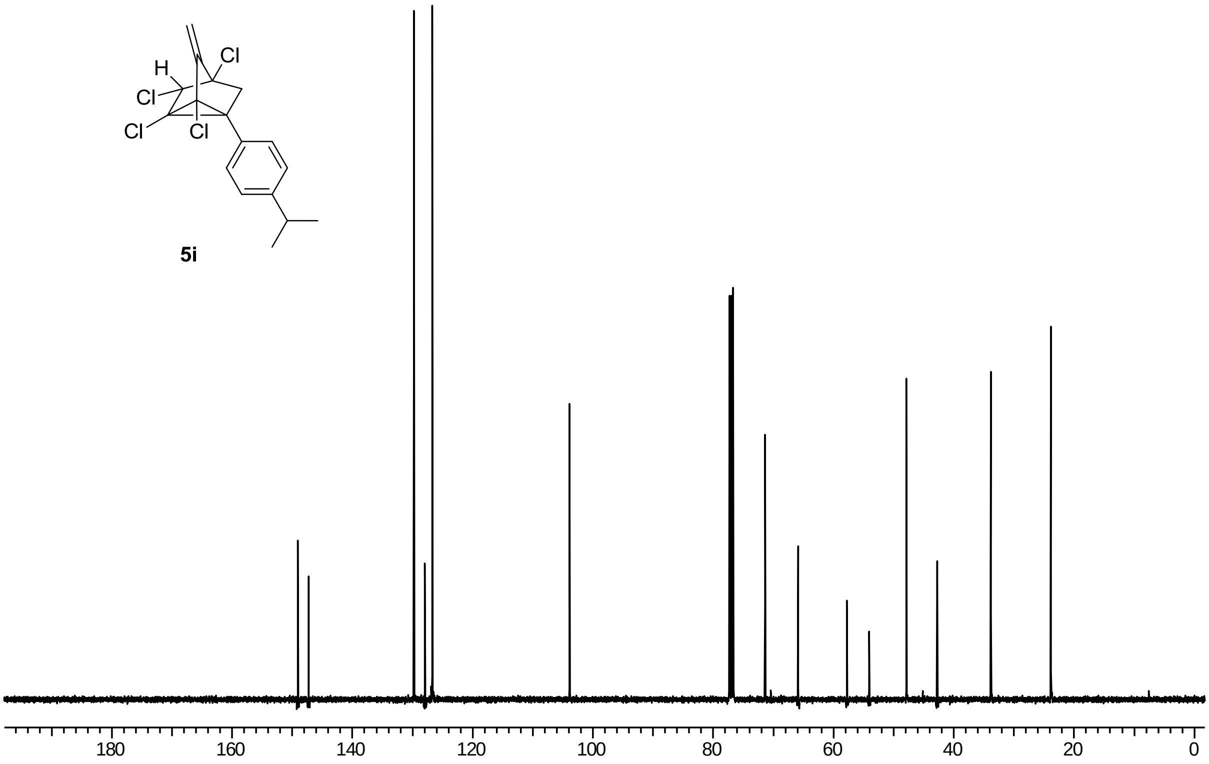

^{1}H NMR (400 MHz) and ^{13}C NMR (100 MHz) of 5j in $CDCl_3$:

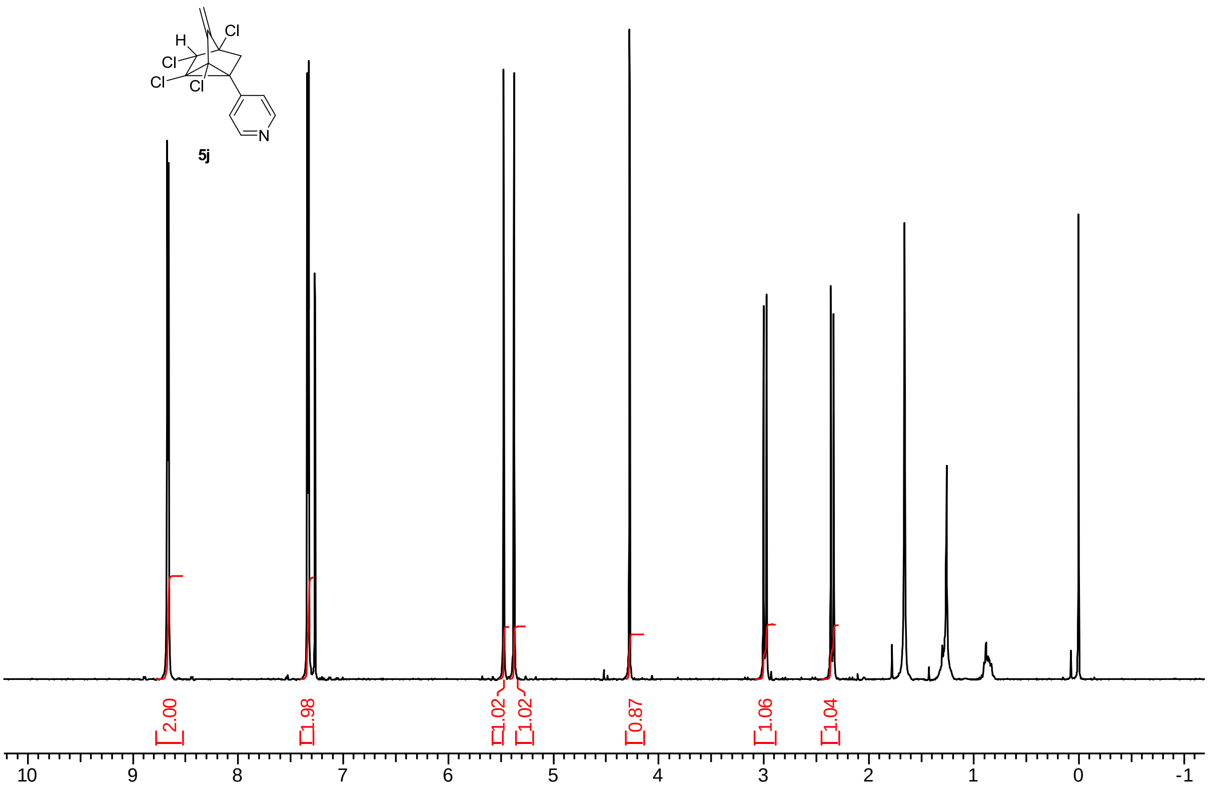

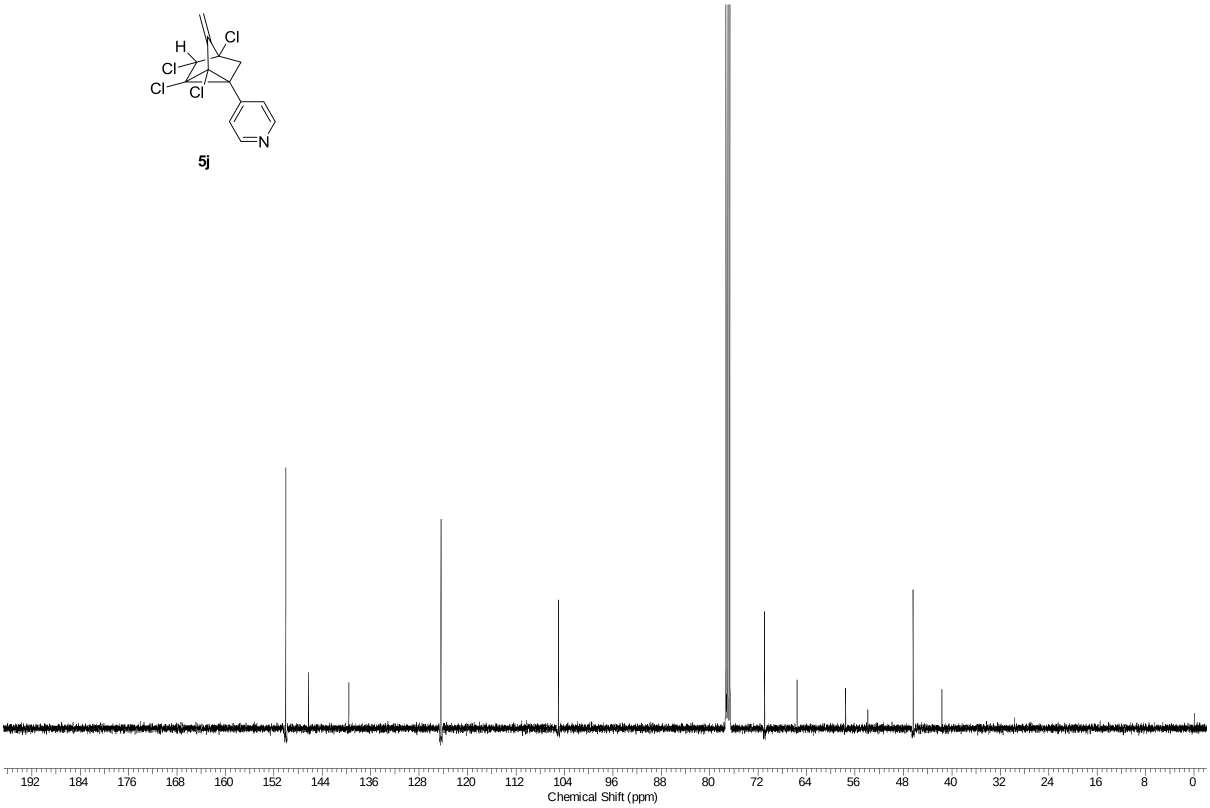

^{1}H NMR (400 MHz) and ^{13}C NMR (100 MHz) of 7 in $CDCl_3$:

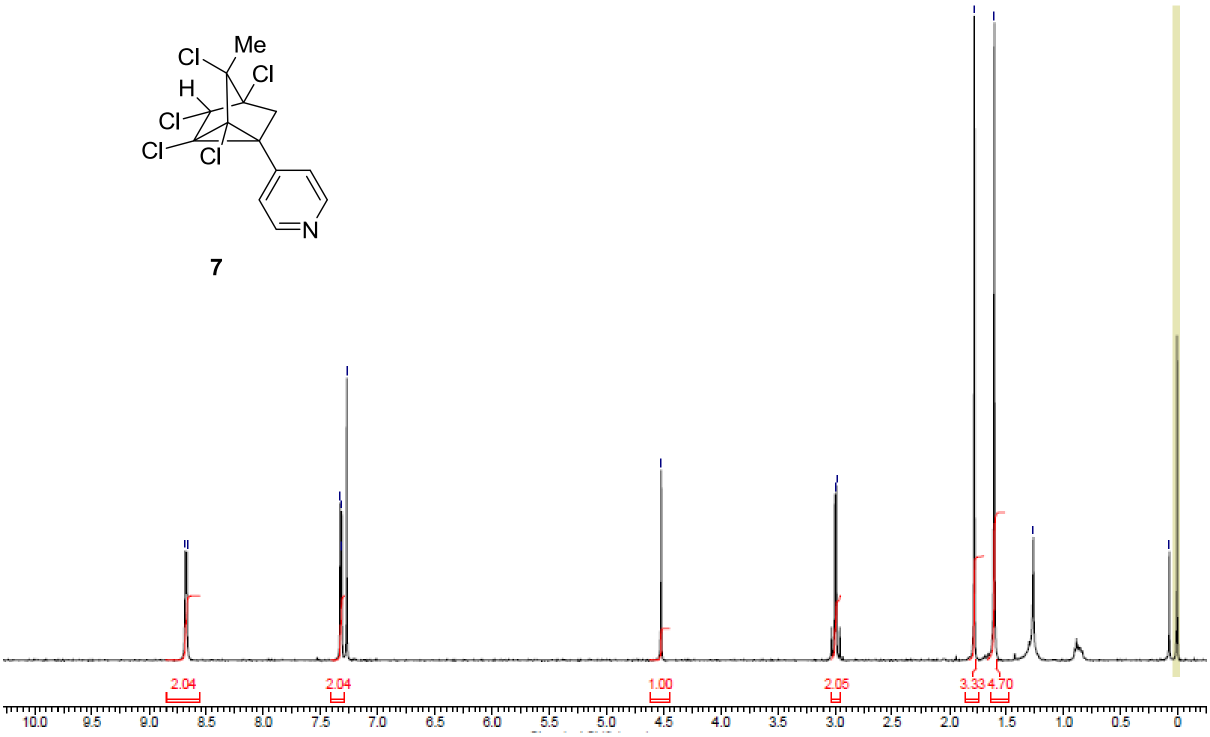

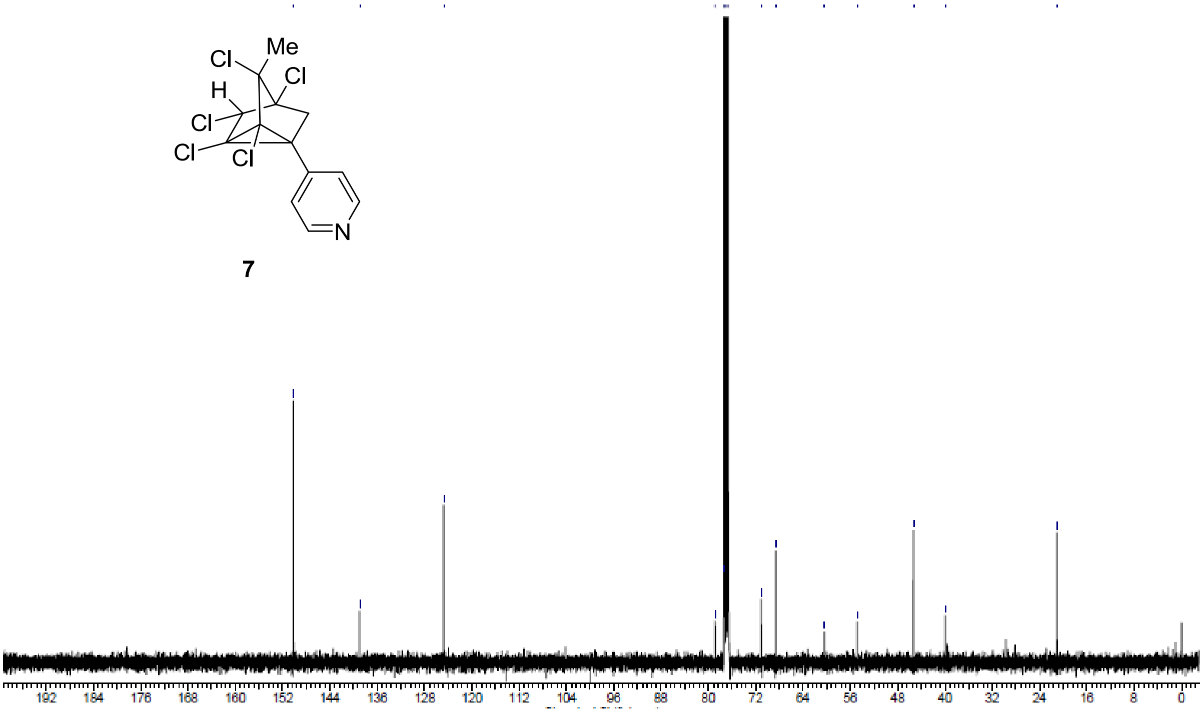

^{1}H NMR (400 MHz) and ^{13}C NMR (100 MHz) of 5k in $CDCl_3$:

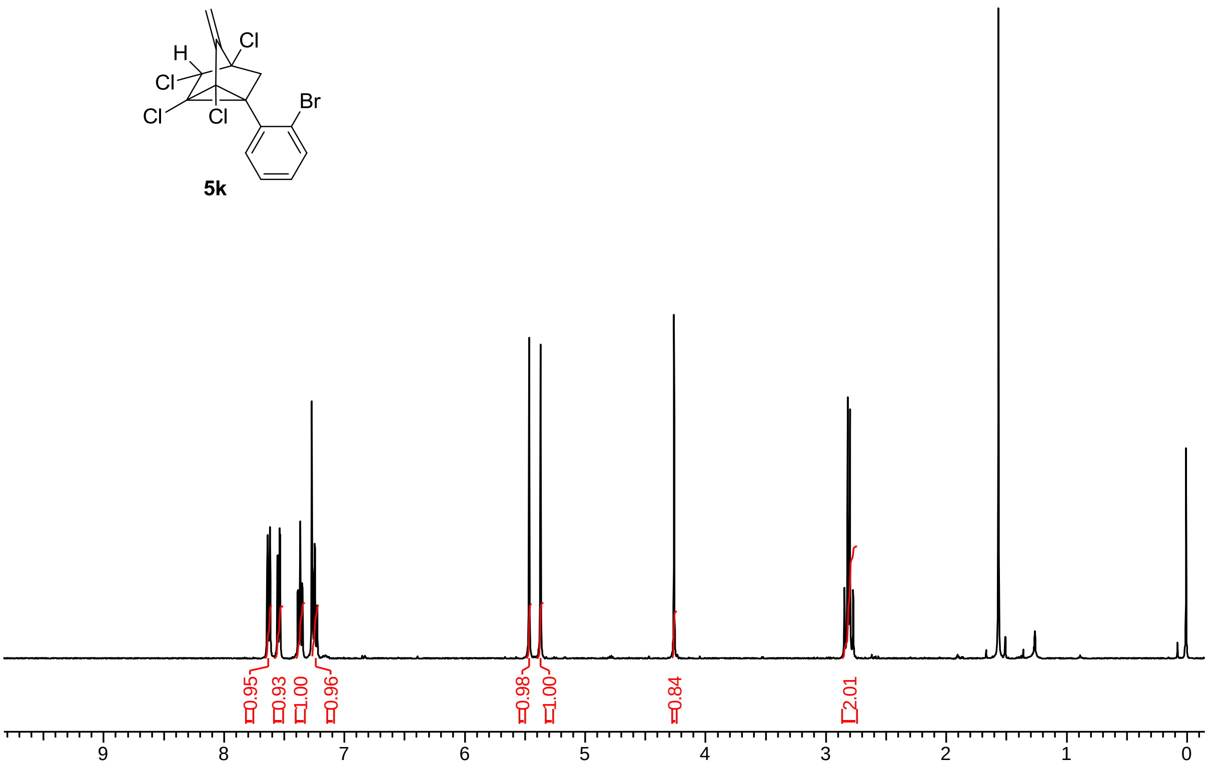

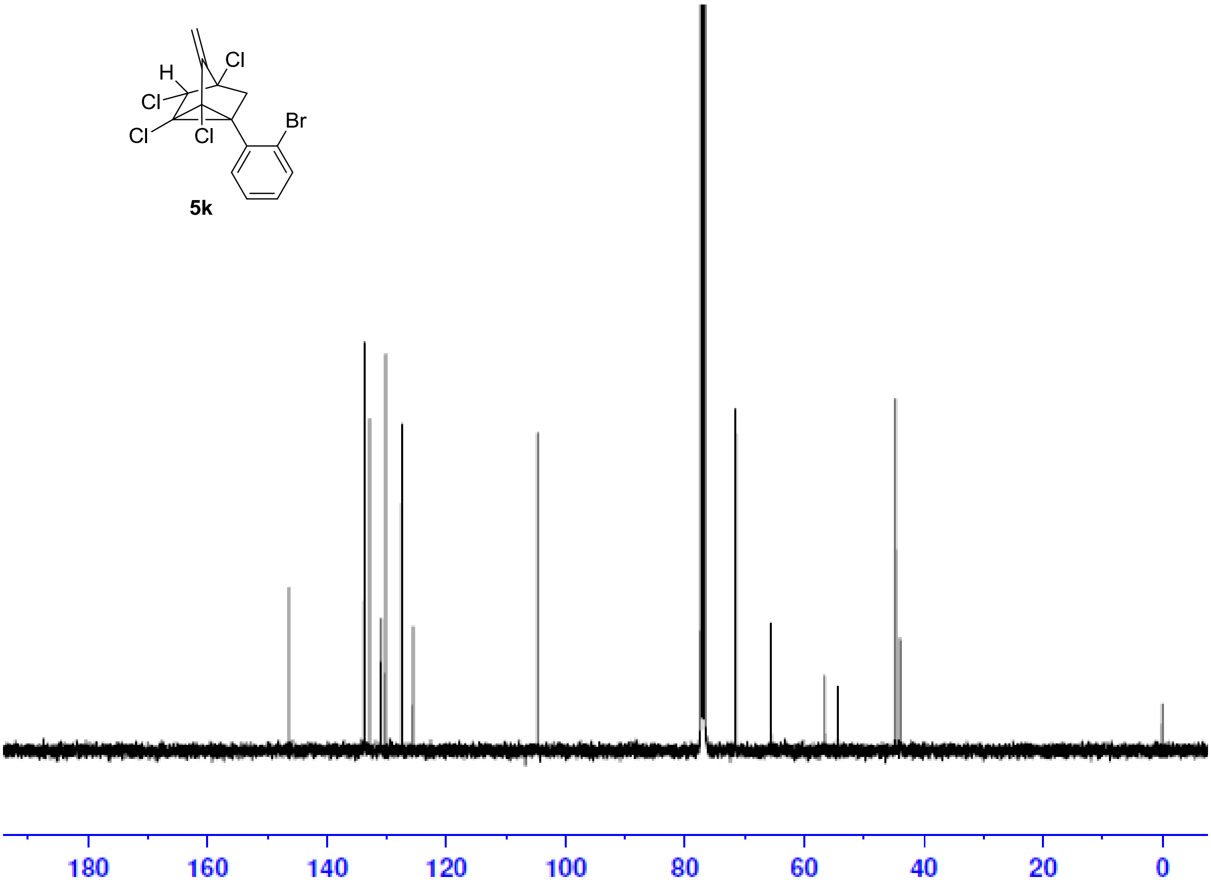

^{1}H NMR (400 MHz) and ^{13}C NMR (100 MHz) of 5l in CDCl$_3$:

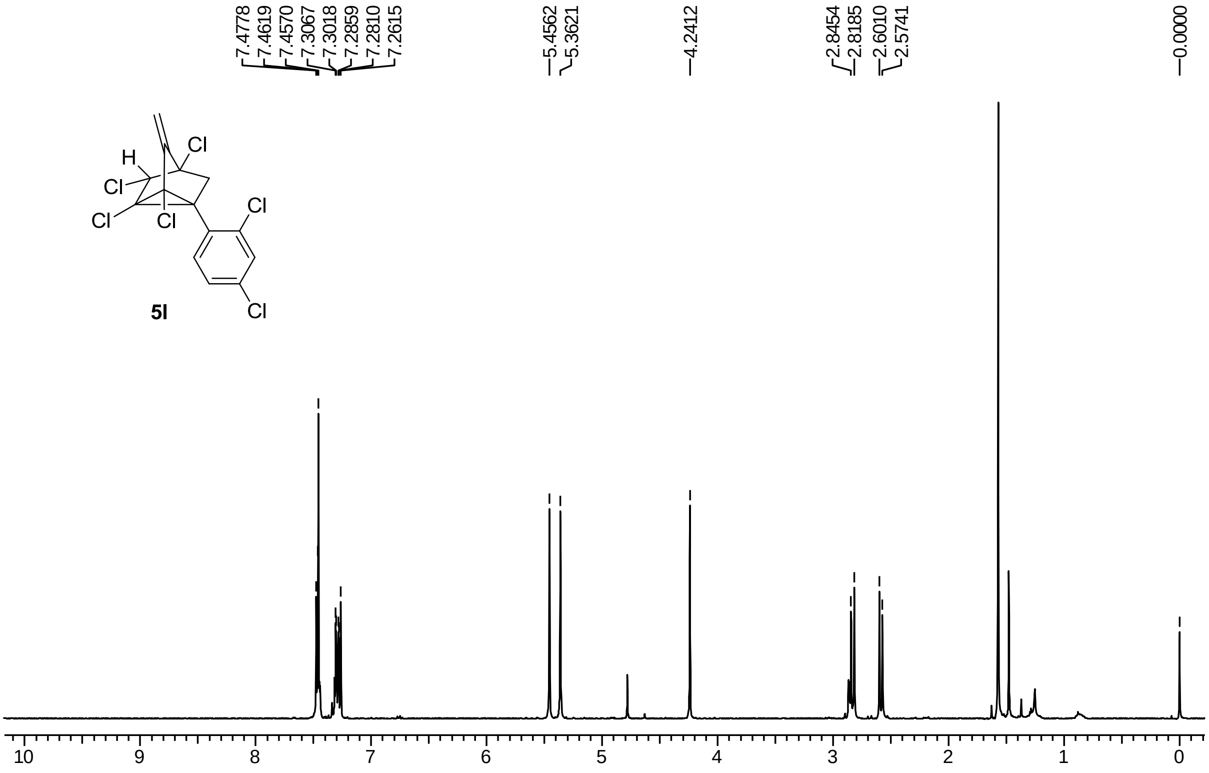

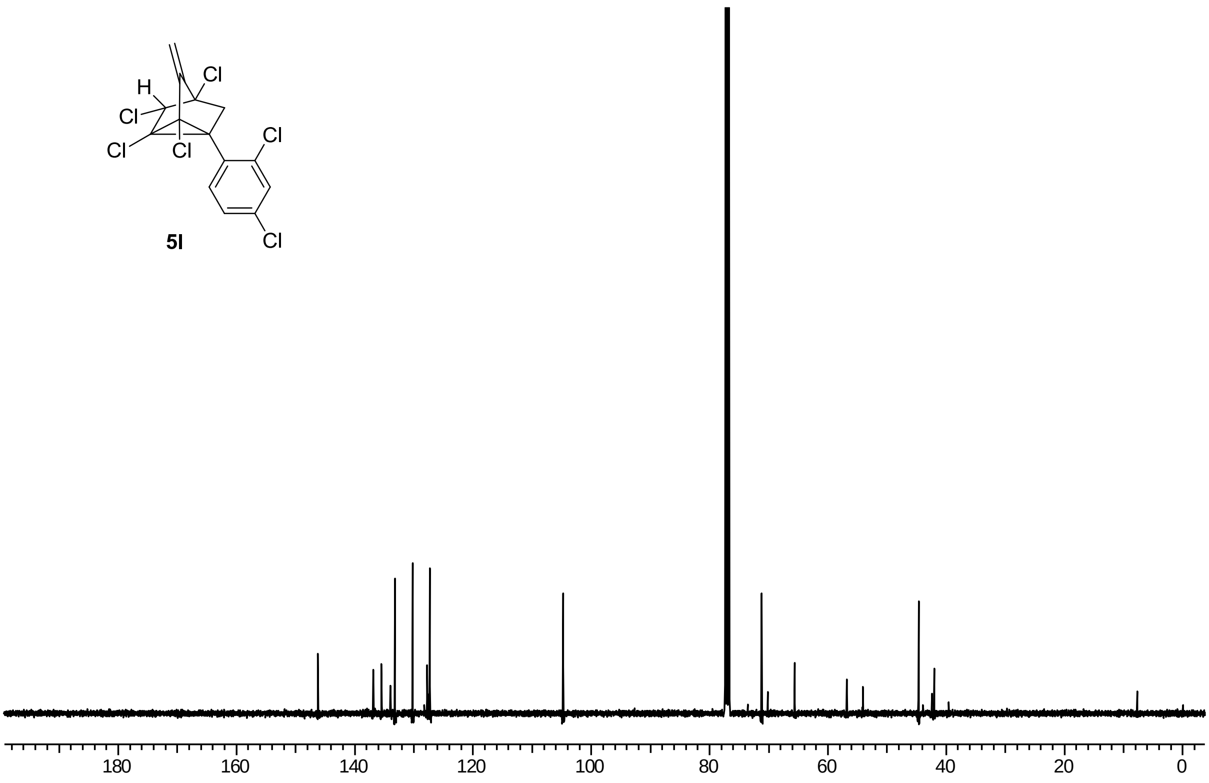

^{1}H NMR (400 MHz) and ^{13}C NMR (100 MHz) of 6c in $CDCl_3$:

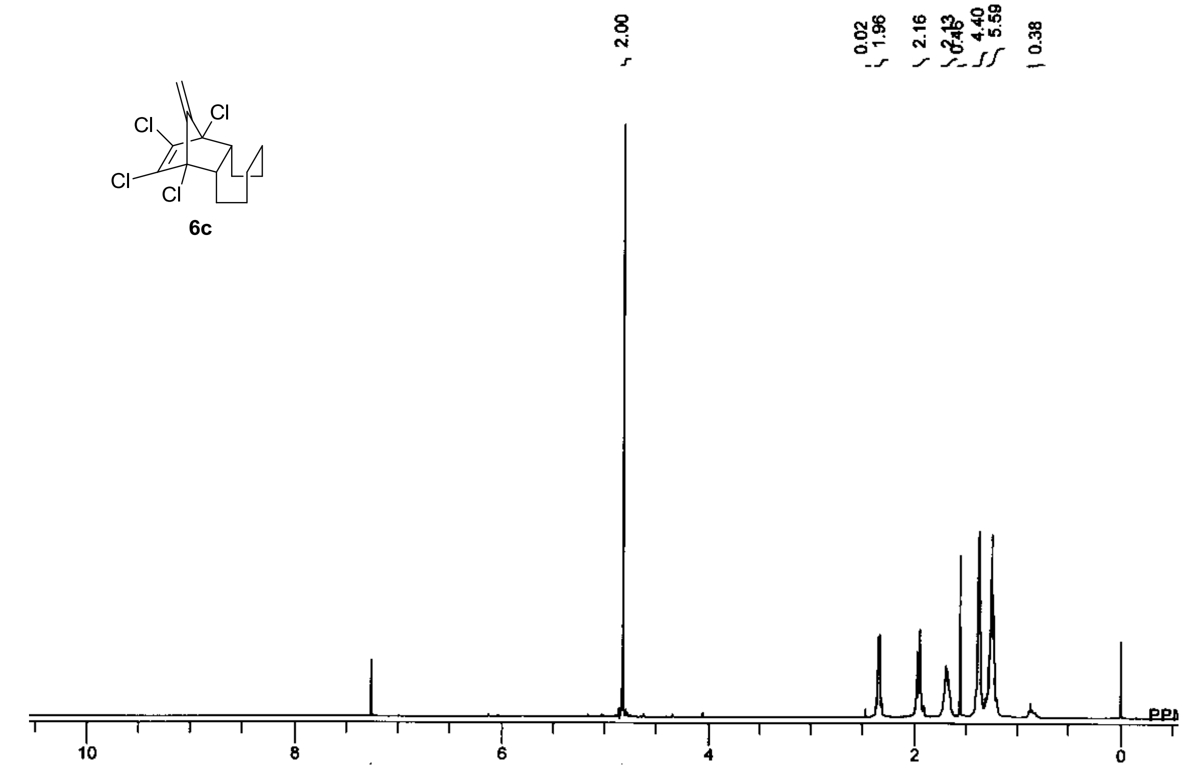

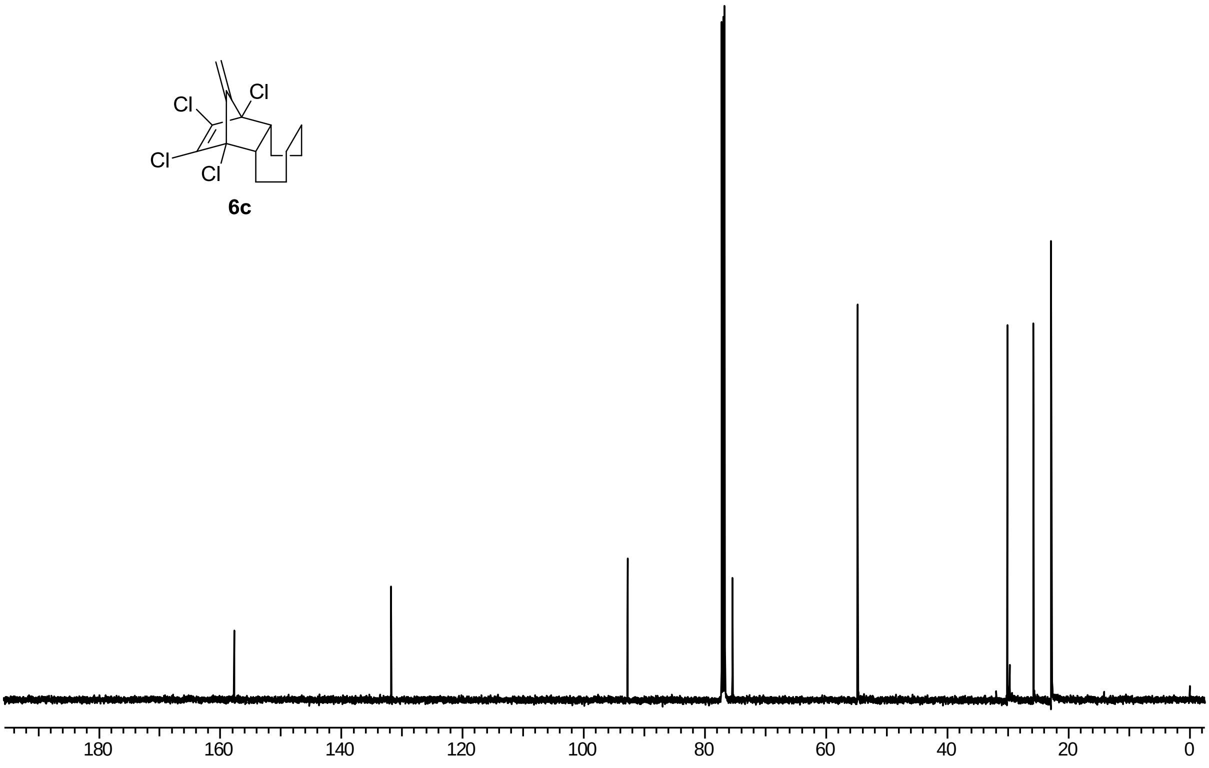

^{1}H NMR (400 MHz) and ^{13}C NMR (100 MHz) of 5m in $CDCl_3$:

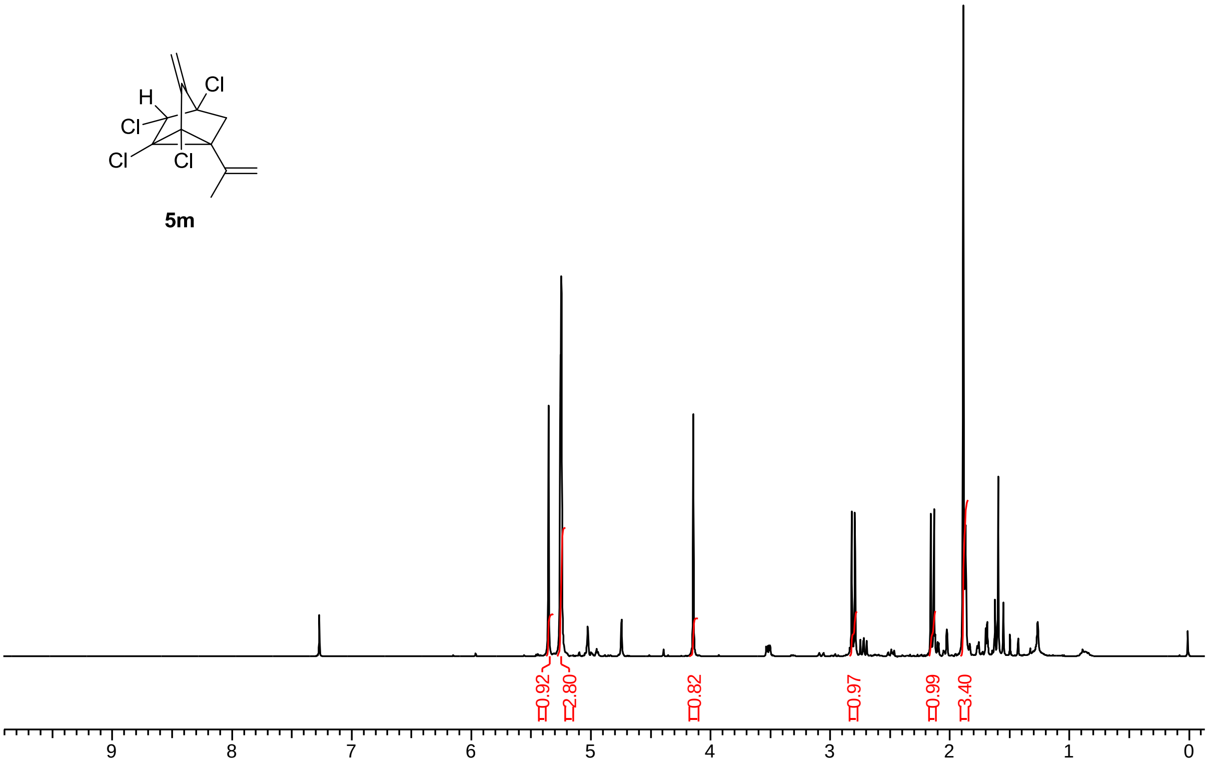

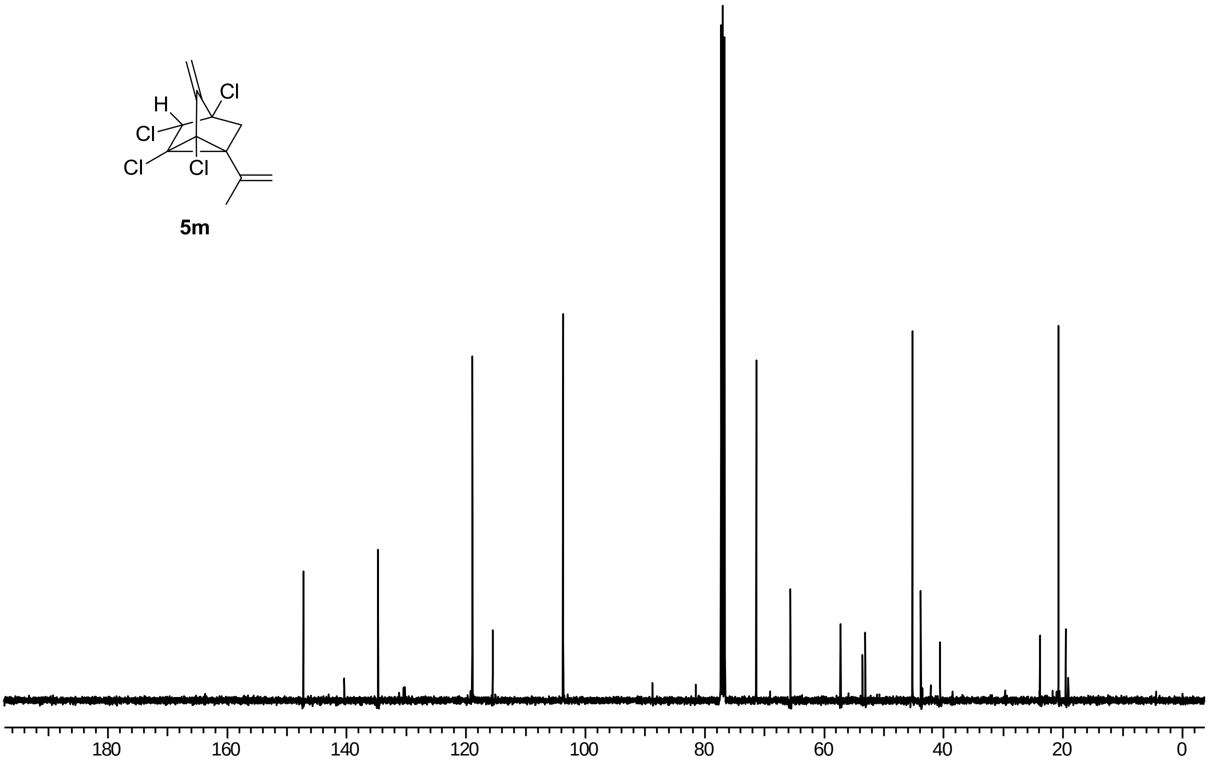

^{1}H NMR (400 MHz) and ^{13}C NMR (100 MHz) of 5n in $CDCl_3$:

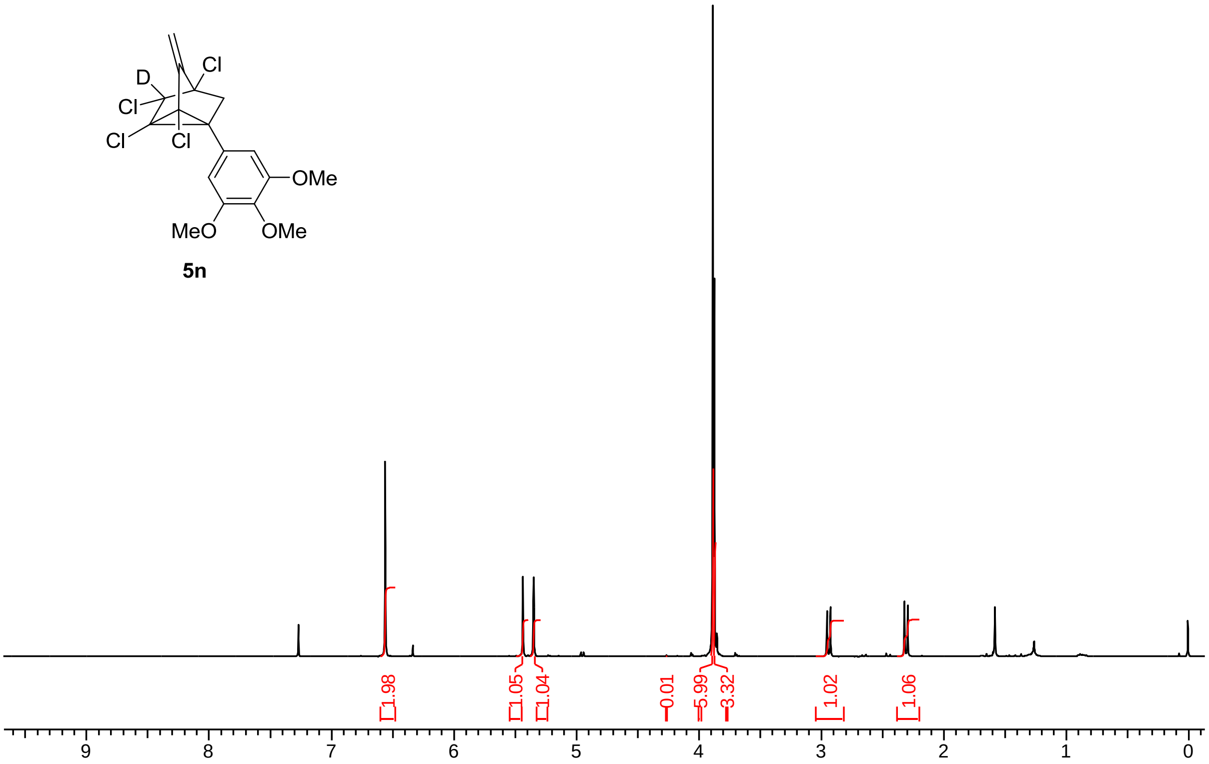

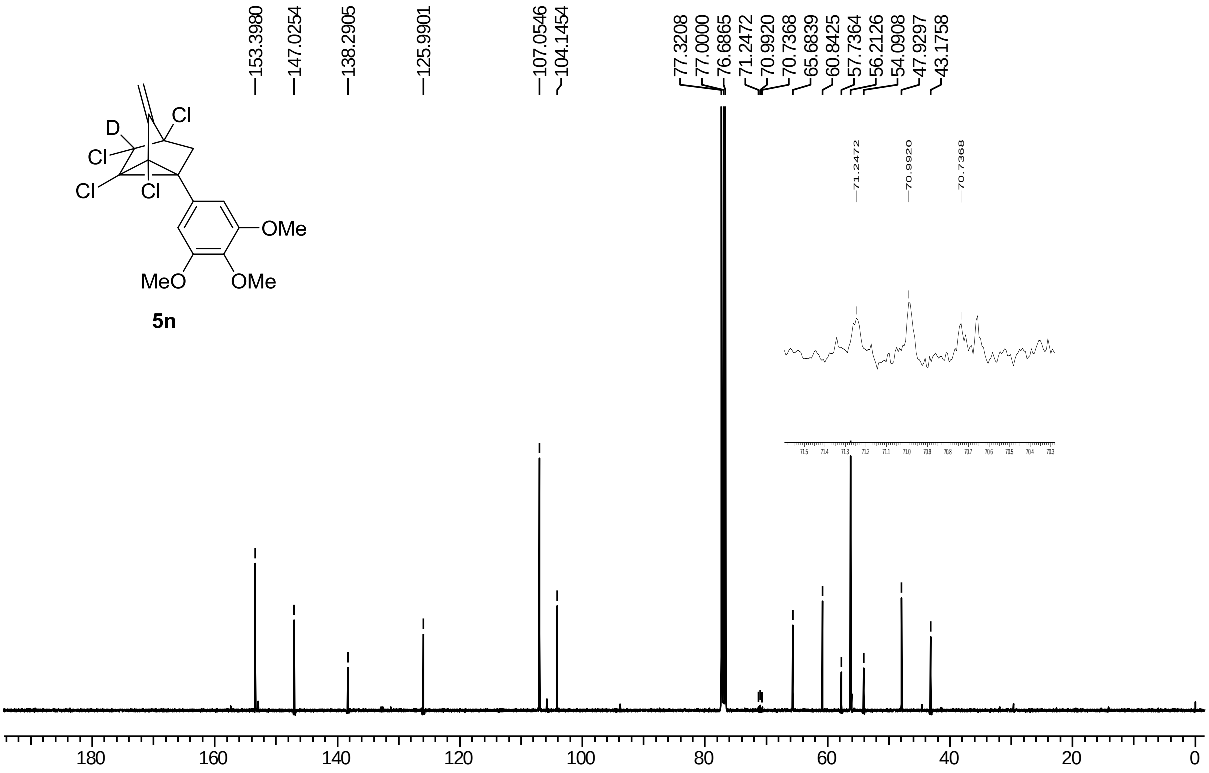

Copies of ^{1}H NMR, ^{13}C NMR spectra of products of acylation reaction of nortricyclens:

^{1}H NMR (400 MHz) and ^{13}C NMR (100 MHz) of 8a in $CDCl_3$:

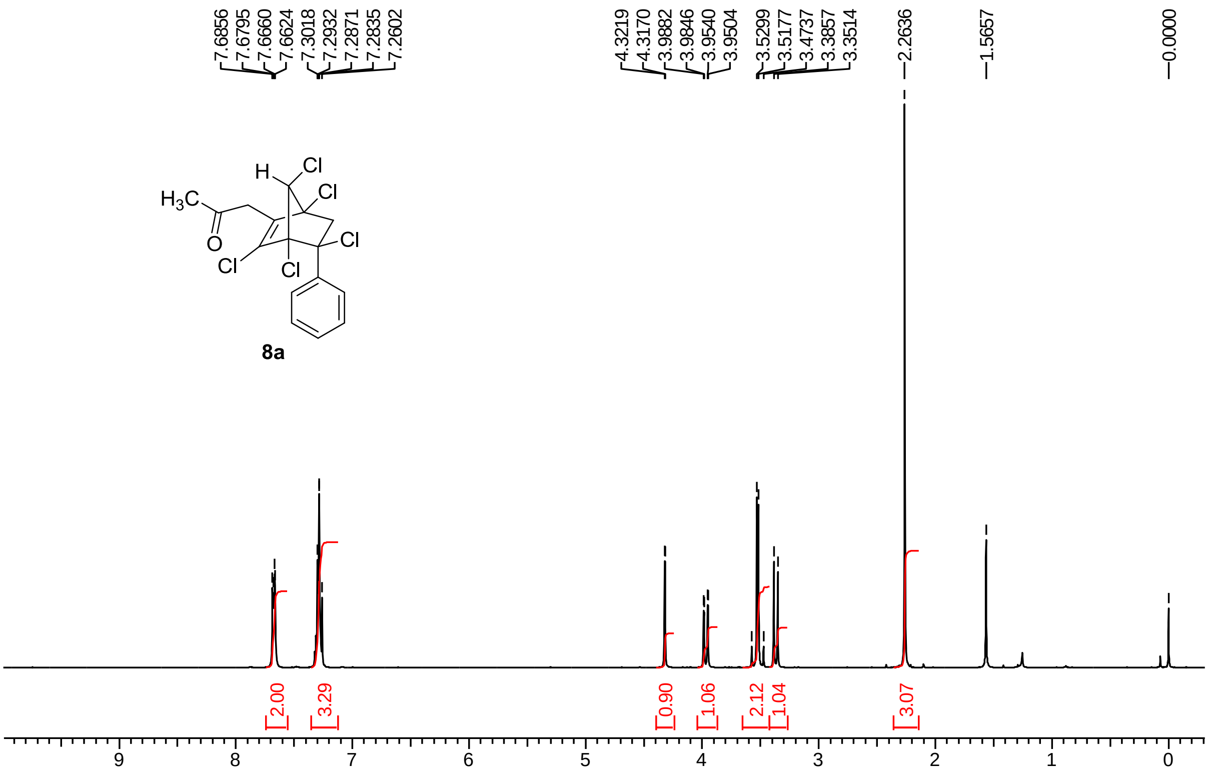

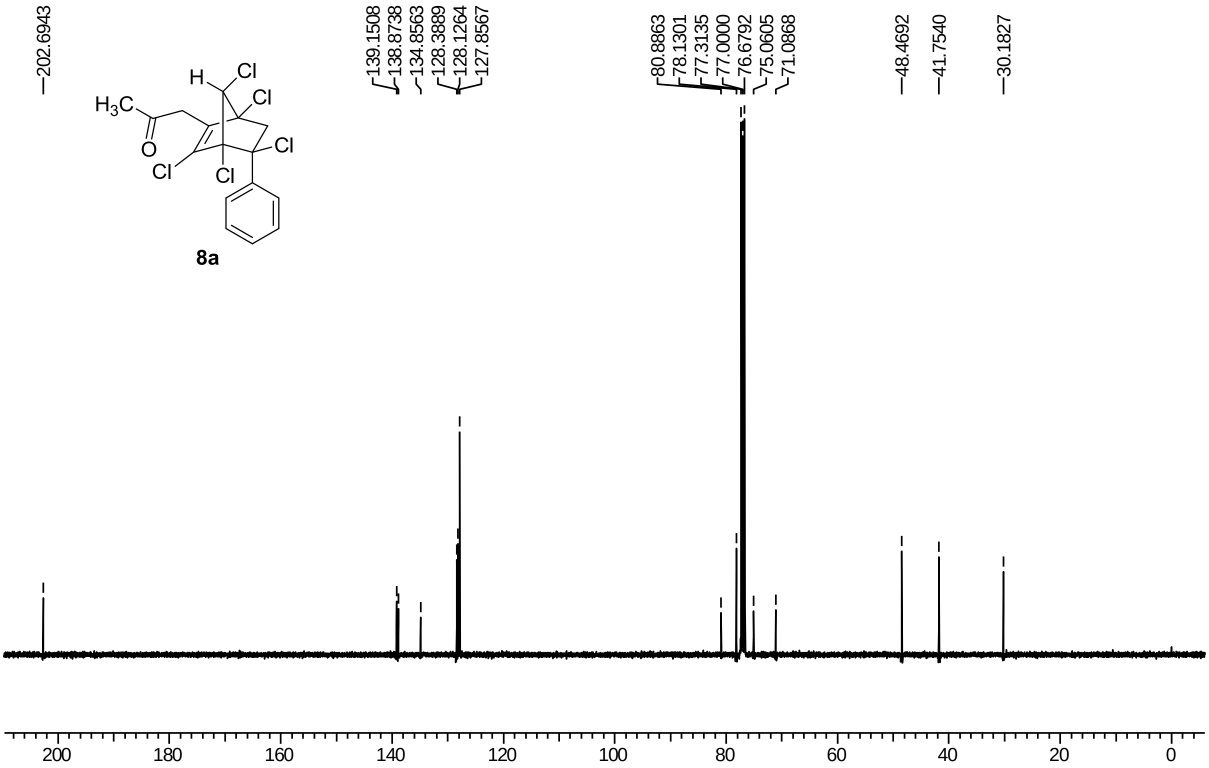

^{1}H NMR (400 MHz) and ^{13}C NMR (100 MHz) of 8b in $CDCl_3$:

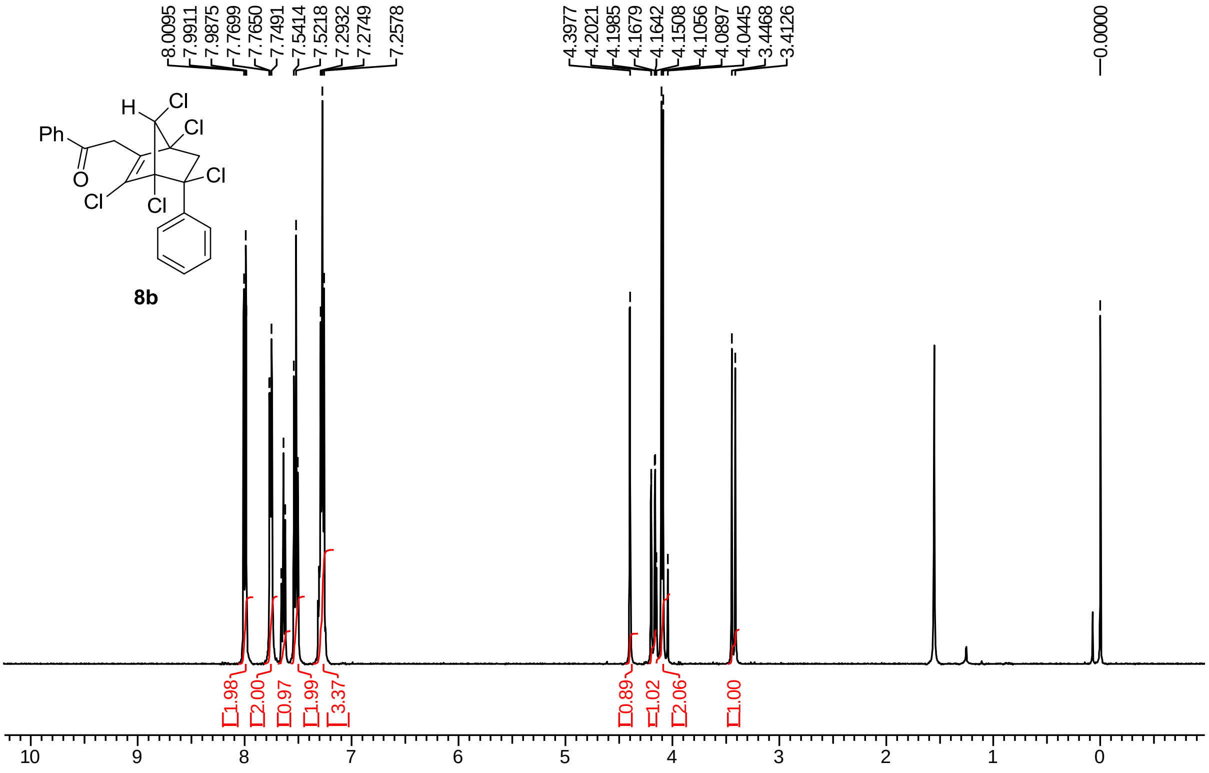

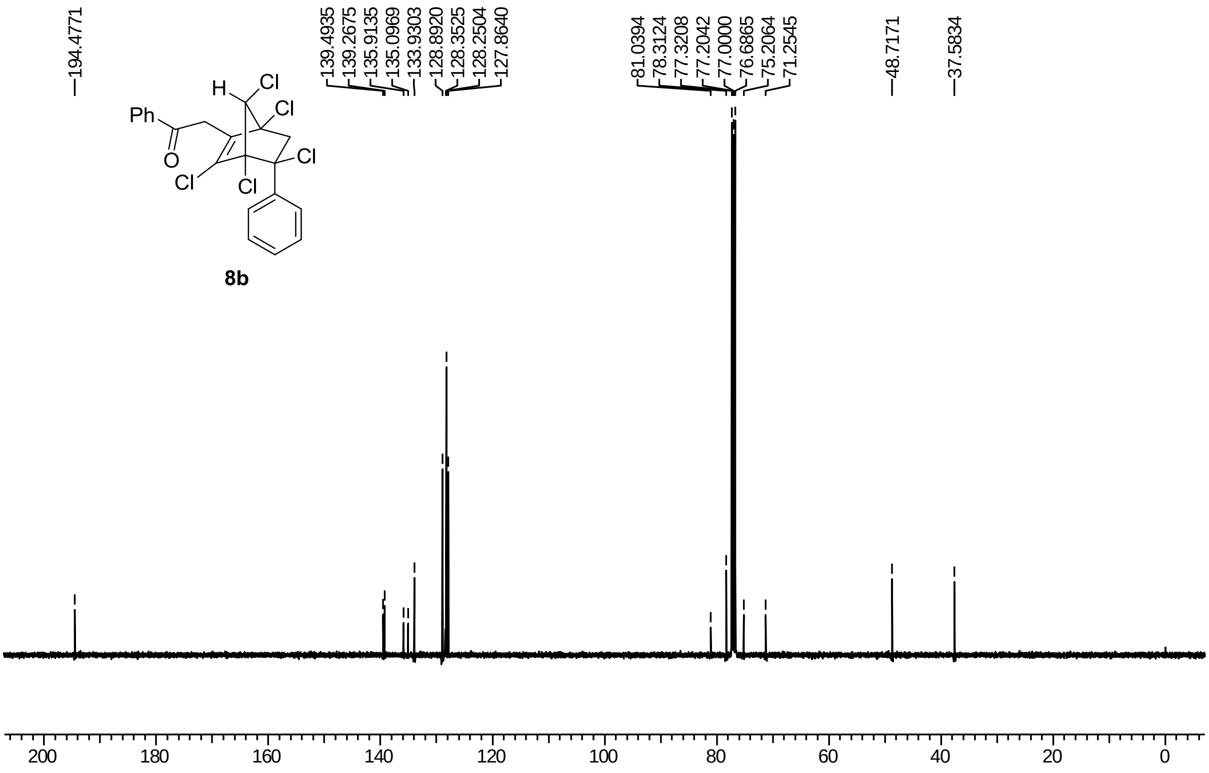

^{1}H NMR (400 MHz) and ^{13}C NMR (100 MHz) of 8c in $CDCl_3$:

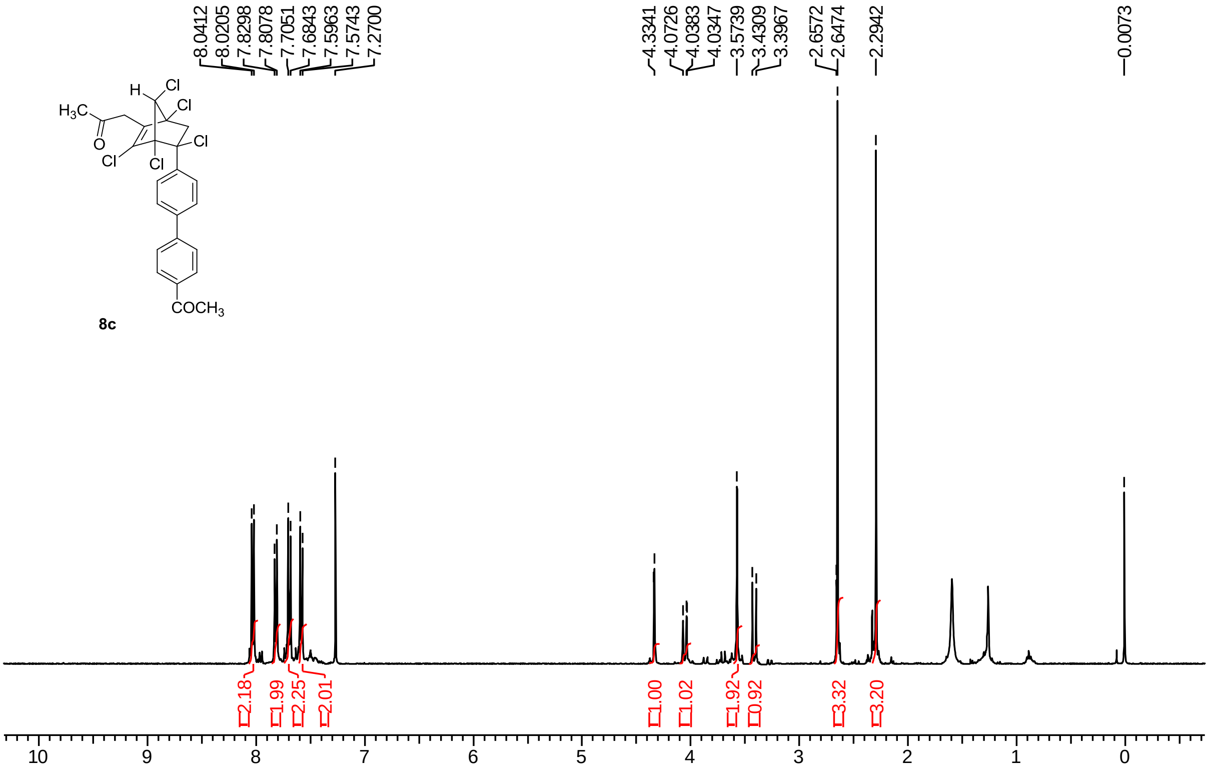

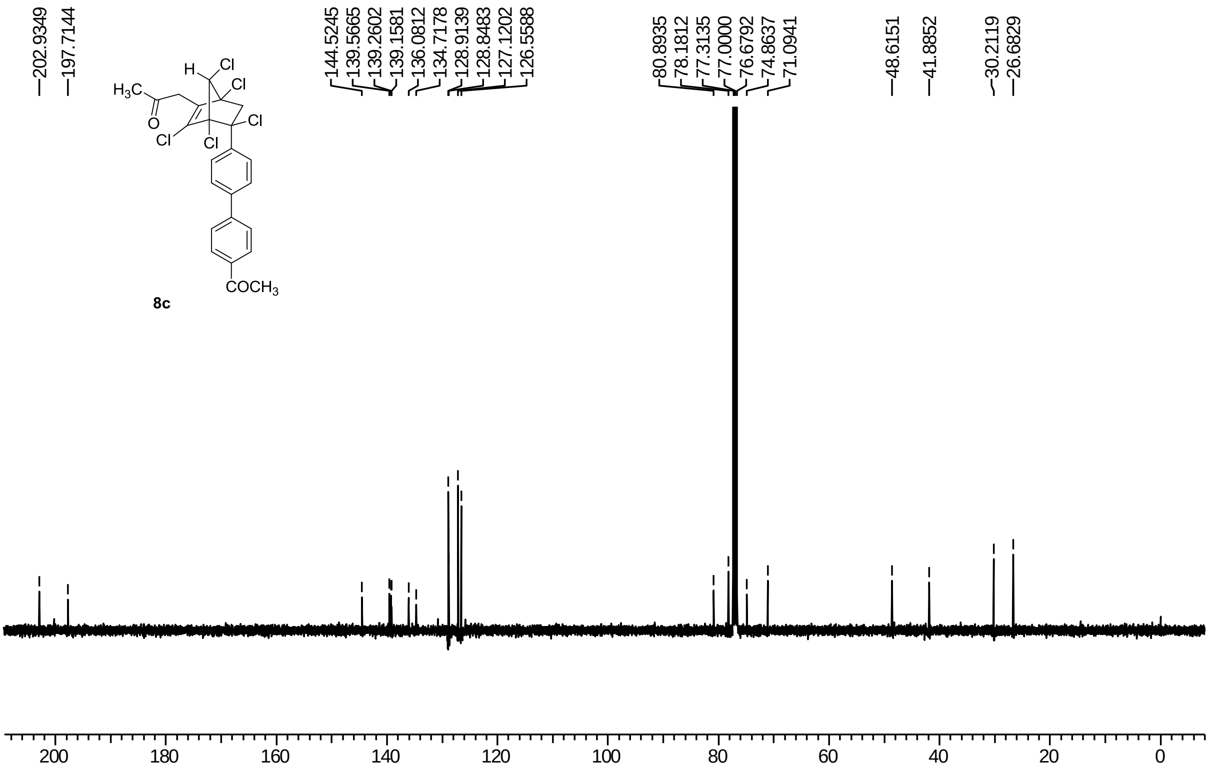

Crystal data and structure refinement of compound 5a.

Empirical formula	$C_{14}H_{10}Cl_4$
Formula weight	320.02
Temperature/K	566(2)
Crystal system	orthorhombic
Space group	Pbca
a/Å	10.7170(4)
b/Å	7.9683(2)
c/Å	32.4915(10)
α/°	90
β/°	90
γ/°	90
Volume/Å^3	2774.65(15)
Z	8
ρ_{calc}mg/mm^3	1.532
m/mm^{-1}	7.561
F(000)	1296.0
Crystal size/mm^3	0.20 × 0.18 × 0.16
2θ range for data collection	9.888 to 141.98°
Index ranges	$-7 \leq h \leq 12$, $-5 \leq k \leq 9$, $-39 \leq l \leq 30$
Reflections collected	5788
Independent reflections	2638[R(int) = 0.0340]
Data/restraints/parameters	2638/0/172
Goodness-of-fit on F^2	1.064
Final R indexes [I>=2σ (I)]	R_1 = 0.0490, wR_2 = 0.1347
Final R indexes [all data]	R_1 = 0.0538, wR_2 = 0.1414
Largest diff. peak/hole / e Å^{-3}	0.38/-0.58
CCDC No.	1021351

Crystal data and structure refinement of compound 8a

Empirical formula	$C_{16}H_{13}Cl_5O$
Formula weight	398.51
Temperature/K	566(2)
Crystal system	triclinic
Space group	P-1
a/Å	7.9750(9)
b/Å	8.5685(11)
c/Å	13.0543(18)
α/°	94.129(11)
β/°	102.281(11)
γ/°	99.211(10)
Volume/Å^3	855.18(19)
Z	2
ρ_{calc}mg/mm^3	1.548
m/mm^{-1}	7.706
F(000)	404.0
Crystal size/mm^3	0.17 × 0.15 × 0.14
2θ range for data collection	6.972 to 141.328°
Index ranges	$-6 \leq h \leq 9$, $-10 \leq k \leq 9$, $-15 \leq l \leq 15$
Reflections collected	5731
Independent reflections	3215[R(int) = 0.0319]
Data/restraints/parameters	3215/0/200
Goodness-of-fit on F^2	1.033
Final R indexes [I>=2σ (I)]	R_1 = 0.0423, wR_2 = 0.1155
Final R indexes [all data]	R_1 = 0.0456, wR_2 = 0.1199
Largest diff. peak/hole / e Å^{-3}	0.33/-0.45
CCDC No.	1021303

Reference

1. Wellman, M. A.; Burry, L. C.; Letourneau, J. E.; Bridson, J. N.; Miller, D. O.; Burnell, D. J. *J. Org. Chem.* **1997**, *62*, 939-946.
